# A Systematic Analysis Revealed the Potential Gene Regulatory Processes of ATRA-Triggered Neuroblastoma Differentiation and Identified a Novel RA Response Sequence in the *NTRK2* Gene

**DOI:** 10.1155/2020/6734048

**Published:** 2020-02-19

**Authors:** Liyuan Guo, Wei Lin, Yidan Zhang, Jing Wang

**Affiliations:** ^1^CAS Key Laboratory of Mental Health, Institute of Psychology, Chinese Academy of Sciences, Beijing 100101, China; ^2^Department of Psychology, University of Chinese Academy of Sciences, Beijing 100049, China

## Abstract

Retinoic acid- (RA-) triggered neuroblastoma cell lines are widely used cell modules of neuronal differentiation in neurodegenerative disease studies, but the gene regulatory mechanism underlying differentiation is unclear now. In this study, system biological analysis was performed on public microarray data from three neuroblastoma cell lines (SK-N-SH, SH-SY5Y-A, and SH-SY5Y-E) to explore the potential molecular processes of all-trans retinoic acid- (ATRA-) triggered differentiation. RT-qPCR, functional genomics analysis, western blotting, chromatin immunoprecipitation (ChIP), and homologous sequence analysis were further performed to validate the gene regulation processes and identify the RA response element in a specific gene. The potential disturbed biological pathways (111 functional GO terms in 14 interactive functional groups) and gene regulatory network (10 regulators and 71 regulated genes) in neuroblastoma differentiation were obtained. 15 of the 71 regulated genes are neuronal projection-related. Among them, *NTRK2* is the only one that was dramatically upregulated in the RT-qPCR test that we performed on ATRA-treated SH-SY5Y-A cells. We further found that the overexpression of the *NTRK2* gene can trigger differentiation-like changes in SH-SY5Y-A cells. Functional genomic analysis and western blotting assay suggested that, in neuroblastoma cells, ATRA may directly regulate the *NTRK2* gene by activating the RA receptor (RAR) that binds in its promoter region. A novel RA response DNA element in the *NTRK2* gene was then identified by bioinformatics analysis and chromatin immunoprecipitation (ChIP) assay. The novel element is sequence conservation and position variation among different species. Our study systematically provided the potential regulatory information of ATRA-triggered neuroblastoma differentiation, and in the *NTRK2* gene, we identified a novel RA response DNA element, which may contribute to the differentiation in a human-specific manner.

## 1. Introduction

The molecular mechanism of neuronal differentiation is not well understood. A well-known regulator in the induction of neural differentiation is retinoic acid (RA), the metabolic product of vitamin A. RA plays important roles in the development, regeneration, and maintenance of the nervous system [1–3]. It is a crucial contributor to the morphogenetic differentiation of pluripotent stem cells and neural progenitor cells [2,4], and the RA signalling regulates the differentiation of primary neurons during embryonic development [5]. RA also plays roles in adult neurogenesis and survival [6], and it regulates the processes of neural plasticity in the adult hippocampus, which is important in learning and memory [7]. It was reported that RA-induced impairments in hippocampal neurogenesis correlate with depression-like symptoms [8]. RA has also been reported to be related to neuropsychiatric disorders such as AD, PD, and MDD and has been considered a potential therapeutic material in the prevention of pathology [9].

RA may enhance neuronal differentiation through a series of changes in epigenetic modification [3,10–14]. The best-studied regulatory mechanism of RA is its ability to bind with and activate nuclear receptors, such as RA receptors (RARs). A protein complex will be recruited after the RAR activation and possess histone deacetylase (HDAC) activity to remodel chromatin nearby and regulate gene expression [15]. The histone acetyltransferase p300, which is required for astrocyte development and axonogenesis [16–18], is a core component in the recruited complex and plays an important role in RA-induced regulation [19].

Several neuroblastoma cell lines are positive for some enzymatic activities of catecholaminergic neurons and can be differentiated to a more mature neuron-like phenotype [20]. Thus, such kinds of cell lines are suitable cell modules for the study of neuronal differentiation and have been widely used in neurodegenerative disease mechanism research [21], detection of neuron-virus interactions [22], and drug effect test [23]. RA can induce the differentiation of neuroblastoma [20]. As described by Nishida et al., all-trans retinoic acid (ATRA) treatment triggered differentiation in all three types of neuroblastoma cell lines (SK-N-SH and two SH-SY5Y subclones, SH-SY5Y-A and SH-SY5Y-E, which were obtained from ATCC and ECACC, respectively) it tested [24].

In this study, we systematically analysed the potential regulatory process of ATRA-triggered neuroblastoma differentiation to provide clues for the molecular mechanism of RA-related neuron differentiation. After revalidating the differentiation of SH-SY5Y-A cells induced by sequential treatment of all-trans retinoic acid (ATRA) and brain-derived neurotrophic factor (BDNF), we reanalysed the public microarray data from three neuroblastoma cell lines (SK-N-SH, SH-SY5Y-A, and SH-SY5Y-E) at their undifferentiated and differentiated stages to get the signature genes of neuroblastoma differentiation. Further bioinformatics analysis was performed to obtain the interacted biological pathways that be may impacted by signature genes and to construct a potential gene regulatory network of neuroblastoma differentiation. We also compared the influence of ATRA and serum starvation (another neuroblastoma differentiation trigger factor) on the expression of neuron projection-related genes identified from the differentiation-related gene regulatory network. The results suggested that the *NTRK2* gene may directly respond to ATRA treatment and the overexpression of *NTRK2* can trigger neuroblastoma differentiation. So RA response-related analyses were further performed on this gene to provide more detailed information.

## 2. Materials and Methods

### 2.1. Cell Culture and Induction

The cell line SH-SY5Y-A was purchased from ATCC (cat. no. CRL-2266^TM^). The cells were grown in a 1 : 1 mixture of DMEM (cat. no. 11885092, GIBCO, USA) and F12 medium (cat. no. 11765062, GIBCO, USA), supplemented with 10% (v/v) foetal bovine serum (FBS) (cat. no. 10091148, GIBCO, USA) as described by Nishida et al. For the observation of cell differentiation, ATRA (cat. no. 223018, Sigma-Aldrich, USA) was added at a final concentration of 10 *μ*M in the mixture medium with 10% FBS. After 5 days in the presence of ATRA, the cells were incubated with 50 ng/ml BDNF (cat. no. B-250, Alomone Laboratories, Israel) in the serum-free medium for 3 days. For the validation of ATRA response differentially expressed genes (DEGs), the cells were cultured in the serum-free medium for 24 hours before ATRA treatment, the ATRA concentration was the same as described above, but the treatment times were 48 hours/6 days, and there was no additional BDNF treatment. For the RA response analysis, the cells were seeded as above and the pan-RAR antagonist AGN193109 (cat. no.sc-210768, Santa Cruz, USA) was added 3 hours before ATRA at a final concentration of 2 *μ*M. Cell lysis for subsequent experiments was performed 2 days after ATRA and/or AGN193109 treatment. For the effects of *NTRK2* overexpression, the cells were seeded in a 12-well plate at an initial density of 2 × 10^5^ cells per well 24 hours before transfection. The full-length *NTRK2* cDNA expression plasmid (cloned in the pCMV6 plasmid) was purchased from Origene, USA (cat. no. RC221794). Transient transfection was performed by using the FuGene HD transfection reagent (cat. no. E2311, Promega, USA). Forty-eight hours later, the cells were incubated with 50 ng/ml BDNF (cat. no. B-250, Alomone Laboratories, Israel) in the serum-free medium for 3 days.

### 2.2. Immunofluorescence (IF)

**Figure 1:**
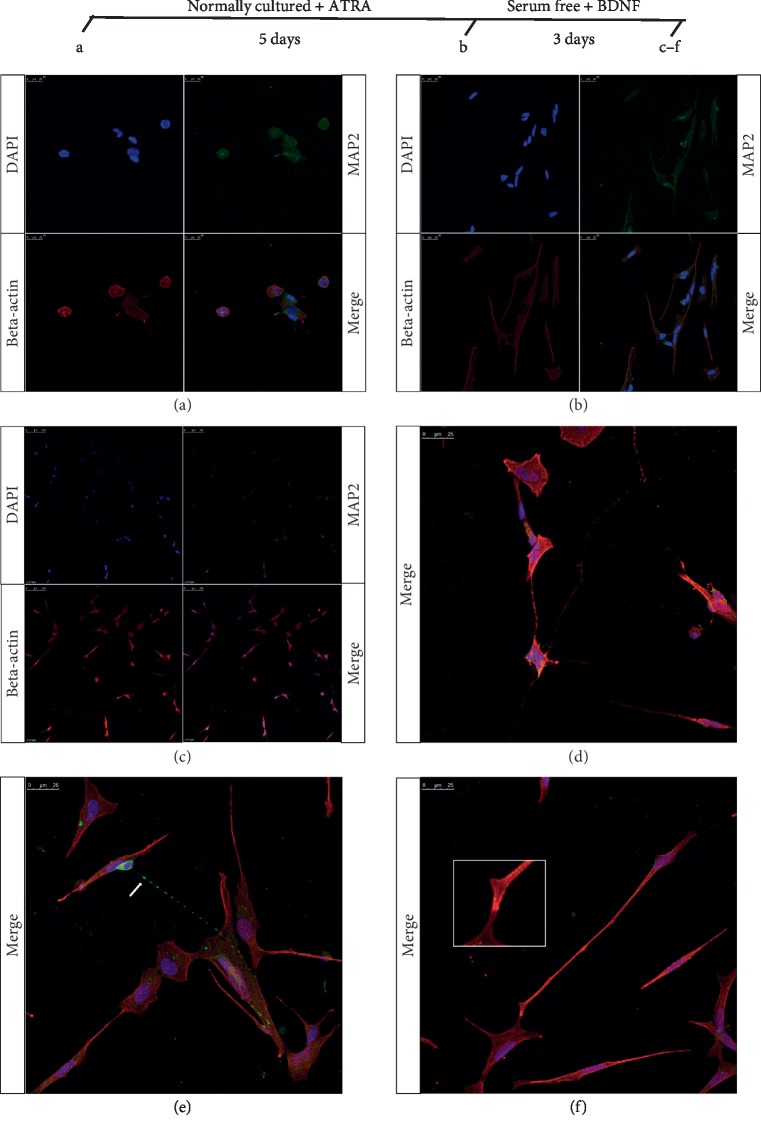
Effect of sequential treatment of ATRA and BDNF on SH-SY5Y-A differentiation. The differentiation of the neuroblastoma cell line SH-SY5Y-A was performed as described by Encinas et al. [25]. Cell morphology (displayed by the cytoskeletal protein beta-actin) and neuron marker (MAP2) localization were observed at several time points during the differentiation. Phase contrast microscopy ×100. Nuclei were stained with DAPI (blue). The cytoskeleton protein beta-actin was labeled with Fluor-594 (red), and neuronal marker protein MAP2 was labeled with Fluor-488 (green). Scale bar represents 100 *μ*m (a–c) and 25 *μ*m (d–f). (a) Undifferentiated SH-SY5Y-A cells. Both substrate-adherent (S-type) and neuroblastic (N-type) cells were observed. The MAP2 proteins were diffused in the cytoplasm. (b) Differentiated SH-SY5Y-A cells that were treated by 10 *μ*M ATRA for 5 days. S-type cells disappeared. In N-type cells, the length of the axon-like structure increased. Intercellular junction structures were observed. (c–f) Differentiated SH-SY5Y-A cells after sequential ATRA and BDNF treatment (ATRA 10 *μ*M for 5days and BDNF 50 ng/ml in the serum-free culture condition for 3days). In (c), the length of fiber-like structures was expended to ten times longer than the cell body. A complex cell network was constructed by multiple axon-like structures. In (d), axon-like structures extended from the cell body and connected to other cells. MAP2 proteins were enriched in the cytoplasm and axon-like structures. In (e), MAP2 proteins in axon-like structures were located in vesicle-like structures (labeled by the white arrow). In (f), the joint parts of cell membrane were thickened. Data are taken from three independent cell preparations (*n* = 3 samples per group).

Immunofluorescence assays were performed to observe the morphological changes of ATRA-treated SH-SY5Y-A cells. Sterilized glass coverslips were placed in 6-Well Clear TC-Treated Multiple Well Plates (Corning, USA), and the cells were plated and treated as described above. At each evaluation time point (as shown in Figure 1), the culture medium was removed from the well and the cells were gently rinsed twice in PBS at room temperature and incubated in 4% paraformaldehyde in PBS for 10 min. After rinsing, the cells were treated with 0.1% Triton X-100 for 10 min and incubated in 0.1% BSA in PBS for 1 hour. Coverslips with blocked cells were removed from the plates and incubated with antibodies targeting MAP2 (cat. no. ab11267, Abcam, USA) and beta-actin (cat. no. A10938R, Solarbio, China) overnight at 4°C. After washing, the coverslips were incubated with Alexa Fluor 488- and 594-conjugated secondary antibodies (cat. nos. ZF-0512 and ZF-0513, ZSGB-BIO, China) for 1 h at room temperature. The nuclei were stained using 1 *μ*g/ml DAPI (cat. no. D9542, Sigma-Aldrich, USA) for 10 min. The slips were observed using a Leica TCS SP8 confocal laser scanning microscope with three lasers at 358, 488, and 594 nm.

### 2.3. DEG Analysis and Gene Ontology (GO) Term Network Analysis

**Figure 2:**
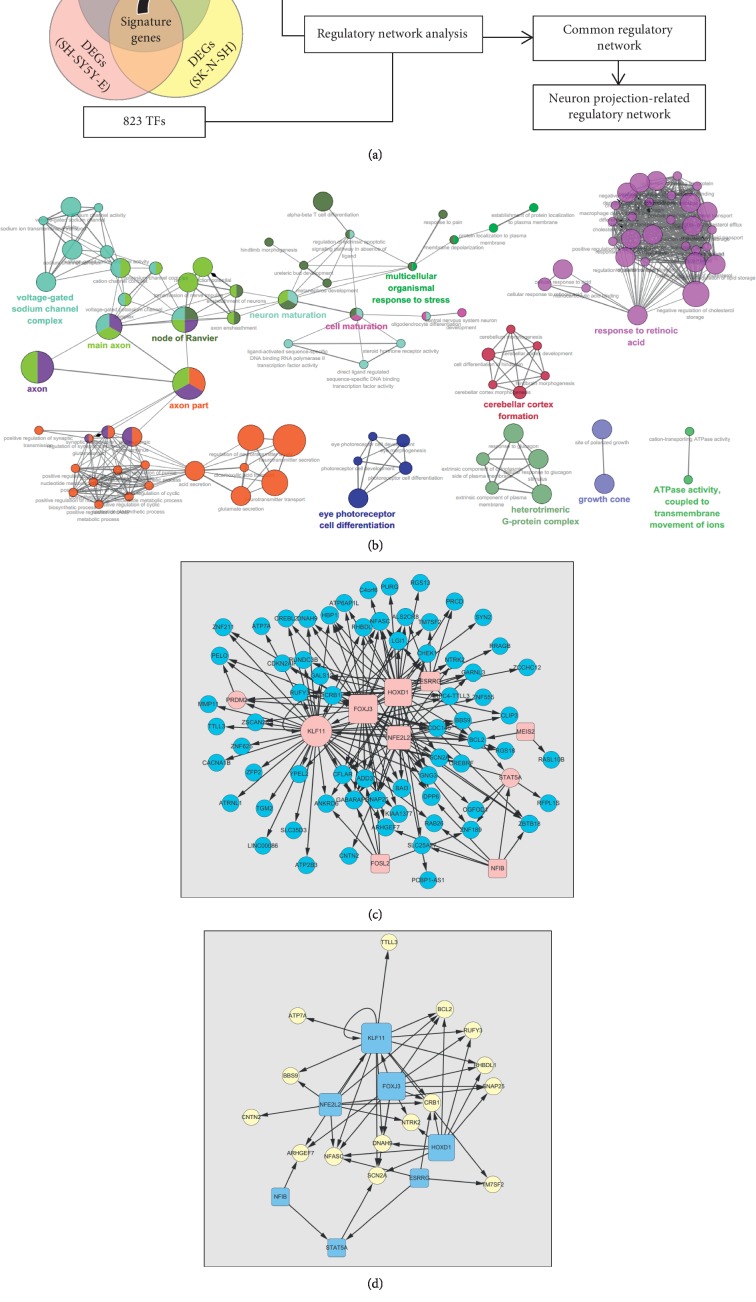
The affected molecular processes and the potential gene regulatory network during neuroblastoma differentiation. (a) A flowchart of the system biology analysis. The expression profiling of three cell lines (SK-N-SH, SH-SY5Y-A, and SH-SY5Y-E) was used to analyse the signature genes and core expression regulatory network of neuroblastoma differentiation. The differentially expressed genes (DEGs) between differentiated and undifferentiated cells were compared. The overlapped DEGs in three cell lines were defined as signatures of neuroblastoma differentiation, and the biological function pathways in that the signatures are involved were analysed. A regulatory network surrounding these signatures was constructed by using the software ARACNe. 823 TFs were treated as candidate regulators. A subnetwork that includes genes involved in neuron project function was then extracted from the regulatory network. (b) The network presents the most significant biological functions of the neuroblastoma differentiation signatures. Size of the nodes: statistical significance of the terms. Edges: degrees of connectivity between terms calculated using kappa statistics. The names of functional pathways included in the same cluster were labeled with the same color. (c) Potential gene regulatory network of neuroblastoma differentiation inferred by the ARACNe. Red nodes: master regulators. Blue nodes: regulated genes. Round nodes: differentiated DEGs. Rectangle nodes: nondifferentially expressed transcription factors. (d) Neuron projection-related genes and their regulators in the gene regulatory network. Round nodes: neuron projection-related genes. Rectangle nodes: regulators.

Nishida et al. detected the expression profiling during the ATRA-triggered differentiation of three neuroblastoma cell lines by microarray analysis. According to the differentiation characteristics, distinct differentiation methods were performed on different cell lines (ATRA treatment for 6 days or sequential ATRA treatment for 5 days and BDNF treatment for 3 days). We downloaded the raw data (CEL files) from the Gene Expression Omnibus (GEO) database with the accession GSE9169. The description of total samples in GSE9169 and the exact samples we used are shown in Table 1. For each cell line, the data of the two stages were compared by using the “ComparativeMarkerSelection” module in GenePattern [26]. The thresholds to identify the DEG were fold change over 2 or under 0.5 and false discovery rate (FDR)<0.05. As shown in Figure 2(a), genes that were differentially expressed during differentiation of all three cell lines are defined as neuroblastoma differentiation signatures. The systematic biological processes related to signatures were evaluated using the Cytoscape plug-in ClueGO [27], with the kappa score threshold 0.4 in the GO term enrichment (right-sided hypergeometric test), and the results were presented as the interacted GO term network.

### 2.4. Regulatory Network Construction

The information-theoretic algorithm “Algorithm for the Reconstruction of Accurate Cellular Networks (ARACNe),” which was developed for the reverse engineering of transcriptional networks from microarray data [28] and has been successfully utilized in brain tumours and other conditions' transcriptional network construction [29], was used to build the potential regulatory network in neuroblastoma differentiation. The ARACNe evaluates transcriptional interactions between gene products, estimates the activity of transcription factors (TFs) from that of their transcriptional targets or regulons, and further identities TFs that are master regulators (MRs) of specific signatures. As shown in Figure 2(a), 823 transcription factors (TFs) (see [Supplementary-material supplementary-material-1]) and 231 neuroblastoma differentiation signatures were used as candidate regulators and targets.

As recommended by Basso et al. and Margolin et al. [30,31], the regulons were inferred with the mutual information (MI) score threshold for *p* < 0.05, and the data-processing inequality (DPI) analysis tolerance was implemented at 0%. MI estimation was improved via 50 rounds of bootstrapping. Master regulator analysis (MRA) was performed using the “MRA-FET” plug-in in the tool bench geWorkbench 2.5.1 [32]. MRs were inferred at an FDR <5%. MRs that overlapped among all three cell lines were termed common MRs. The interactions between common MRs and the signatures were revalidated by TF-promoter affinity analysis. Promoter regions from the University of California Santa Cruz (UCSC) genome browser database [33] and transcription factor binding site (TFBS) (both validated and predicted) from the database SwissRegulon [34,35] of signatures were compared based on chromosome location information. Common MRs with affinity for the promoters of their targets were used to construct the common neuroblastoma differentiation regulatory network. GO pathway cluster enrichment analysis was performed on genes in regulatory networks by using the online tool DAVID [36,37], and the subnetwork that was constructed by neuronal projection-related genes was extracted.

### 2.5. RT-qPCR

**Figure 3:**
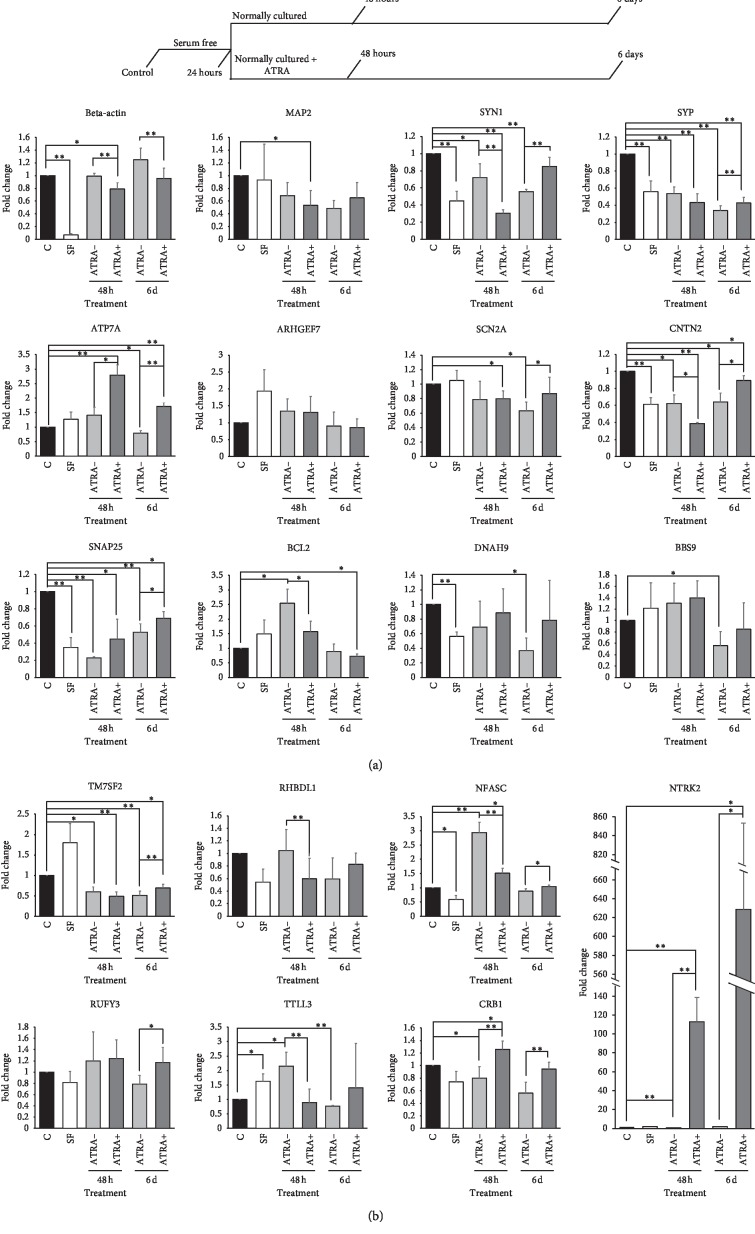
*NTRK2* dramatically expressed after ATRA treatment and continued to increase under ATRA conditions. ATRA can trigger the differentiation of all three neuroblastoma cell lines, but a further BDNF treatment in the serum-free condition is also required for the complete differentiation. Considering that the serum-free condition can also induce the neuroblastoma differentiation, we cultured the SH-SY5Y-A in the serum-free condition for 24 h and changed the medium as a complete medium with or without ATRA (as shown in the top of Figure 3). The expression profiling of beta-actin, three neuron markers, and 15 neuron projection-related genes in normally cultured cells (C), cells cultured in the serum-free condition for 24hours (SF), cells treated by ATRA after serum starvation (ATRA+, 48 h and 6 d), and cells cultured in the normal condition after serum starvation (ATRA−, 48 h and 6 d) was estimated by RT-qPCR; the expression level of the *GAPDH* gene was used as an internal reference. Among all tested genes, the expression level of the *NTRK2* gene was sharply increased by more than a hundredfold after ATRA treatment and rose continuously in the ATRA-positive condition. Data are taken from three independent cell preparations (*n* = 3 samples per group). ^*∗*^*p* value<0.05; ^*∗∗*^*p* value<0.01.

The RT-qPCR assay was performed to estimate the expression changes of differentiation-related genes in the serum-free condition and ATRA condition. The design of cell treatment and observation is shown in Figure 3. For cells at each time point, total RNA was isolated using TRIzol (cat. no. 15596026, Life Technologies, USA). Approximately 200 ng of RNA was used for cDNA synthesis using the SuperRT cDNA Synthesis Kit (cat. no. CW0741, CoWin Biosciences, China) according to the manufacturer's instructions. For qPCR analysis, 1 *μ*l of cDNA was added to the reaction mix containing 10 *μ*l of UltraSYBR Mixture (cat. no. CW0957, CoWin Biosciences, China). The primer sequences were designed using Primer-BLAST in NCBI and are shown in Table 1. Amplification was carried out with the following protocol: denaturation for 10 min at 95°C, followed by 30 s at 94°C and 1 min at 60°C for 40 cycles, finishing with 10 min at 60°C. The expression levels of all evaluated genes were calculated using the ∆∆C*t* method [38]. Sample C*t* values were normalized to the C*t* values of the *GAPDH* gene. Similarly, the RT-qPCR assay was also performed to verify the expression level of the *NTRK2* gene in *NTRK2*-transfected cells.

### 2.6. *NTRK2*-Related RAR Binding Site Analysis

**Figure 4:**
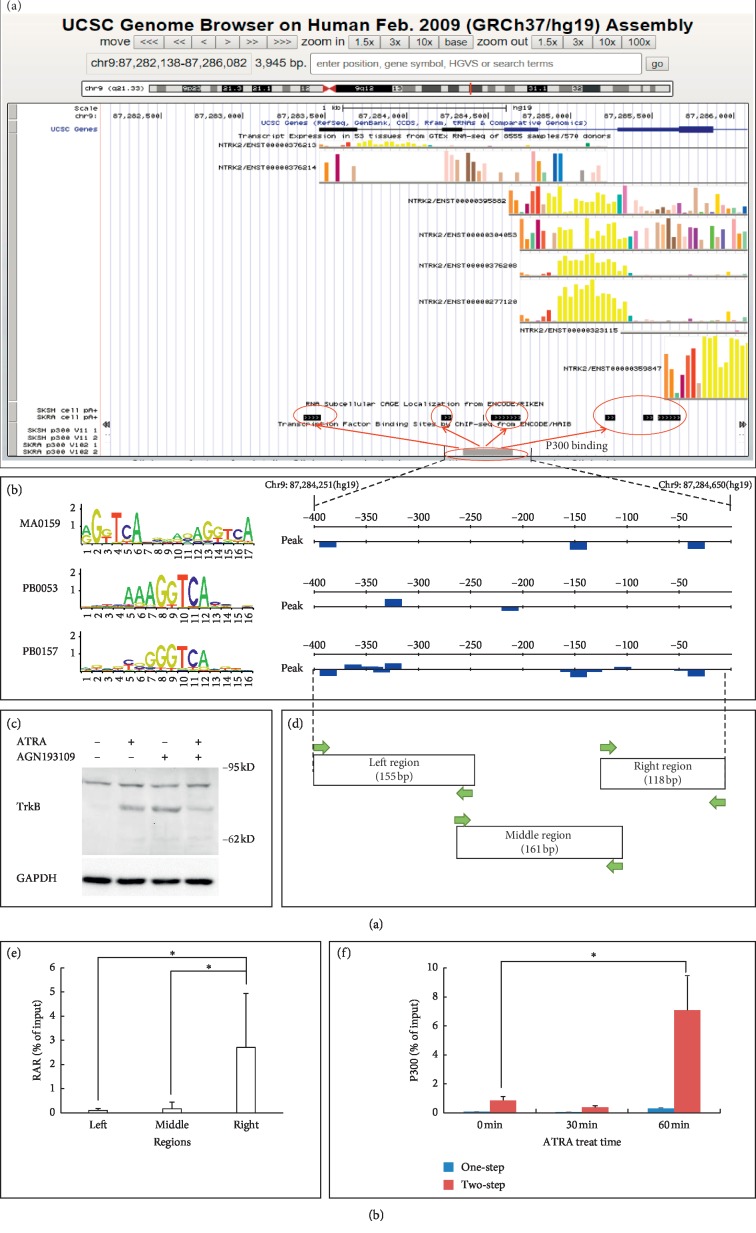
A novel RA response DNA element in the *NTRK2* gene was active after ATRA treatment. One of the main mechanisms of RA is that it influences gene expression to activate the nuclear receptor RAR binding with the RA response DNA element at or nearby the gene promoter region and then recruits P300 to trigger downstream epigenetic modifications. We hypothesized ATRA directly regulates the *NTRK2* gene via this mechanism and analysed the potential RA response DNA element in the *NTRK2* gene. (a) *NTRK2* transcripts in 53 human tissues (queried from the GTEx database), *NTRK2* transcript start sites (TSSs) in normally cultured and ATRA-treated SK-N-SH cells (the track “SKSH cell pA+”and “SKRA cell pA+,” queried from the ENCODE project), and P300 binding peaks in normally cultured and ATRA-treated SK-N-SH cells (the track “SKSH cell P300”and “SKRA cell P300,” with two repeats, queried from the ENCODE project) were aligned in the UCSC gbrowser [40]. Neither TSSs nor p300 binding peak was observed in normally cultured SK-N-SH cells. But in ATRA-treated SK-N-SH cells, several *NTRK2* TSSs were detected and a potential P300 binding peak was found near TSSs in one of the two repeat experiments. SKSH: normally cultured SK-N-SH cells. SKRA: ATRA-treated SK-N-SH cells. (b) The predicted transcript factor binding sites (TFBSs) of the RAR in the potential P300 binding region (400 bps long). The TFBS analysis was performed with the online tool RSAT [41] by using three RAR binding matrixes (MA0159, PB0053, and PB0157, obtained from the database JASPAR). (c) The influence of ATRA and the RAR antagonist AGN193109 on the expression of TrkB protein (encoded by the *NTRK2* gene) was estimated by using western blotting. Data are taken from three independent cell preparations. (d) Primers for chromatin immunoprecipitation (ChIP) were designed to cover the 400 bps long region. According to their relative positions, the three parts covered by different primer pairs were named the “left region,” “middle region,” and “right region.” (e) In normally cultured SH-SY5Y-A cells, DNA co-immunoprecipitated by RAR protein was amplified by using primers that target the left, middle, and right regions, and the quantitative result was shown as percent of the total input. Data are taken from three independent preparations (*n* = 3 samples per group). ^*∗*^*p* value<0.05. (f) In ATRA-treated SH-SY5Y-A cells, DNA co-immunoprecipitated by P300 protein in both traditional ChIP analysis (one-step) and two-step ChIP analysis was amplified by using primers targeting the right region, and the quantitative result was shown as percent of the total input. Data are taken from three independent cell preparations (*n* = 3 samples per group). ^*∗*^*p* value<0.05.

P300 binding regions in RA-treated neuroblastoma could be considered potential RA response regions. The Genotype-Tissue Expression (GTEx) project and the Encyclopedia of DNA Elements (ENCODE) project provide transcripts and protein-DNA element binding information in many tissue and cell conditions [39,40]. In the UCSC genome browser [33], the hg 19 assembly, the 5′ sequence of the *NTRK2* gene from RefSeq, transcripts of *NTRK2* in 53 human tissues from GTEx, transcript start sites (TSSs), and P300 peak regions in normally cultured and ATRA-treated SK-N-SH cells from ENCODE were queried and aligned. The TSSs were identified by using long polyA RNA sequencing (RNA-seq), and P300 peak regions were detected by using the chromatin immunoprecipitation-sequencing (ChIP-seq) assay. As shown in Figure 4(b), the 400 bps long region surrounding a P300 binding peak near the *NTRK2* TSSs was selected for RAR binding site prediction. The binding site prediction was performed by using the online analysis tool RSAT [42]. Three RAR binding matrixes from JASPAR, a database of TF binding profiles [43], were used in the prediction.

### 2.7. Western Blotting

The western blotting assay was used to test the changes of the protein TrkB (which is coded by the *NTRK2* gene) after RAR agonist and/or antagonist treatment. 50 *μ*g total proteins were subjected to SDS-PAGE (10%, w/v) and transferred onto polyvinylidene difluoride (PVDF) membranes. The membranes were blocked with bovine serum albumin (BSA) and incubated with appropriate primary antibodies at 4°C overnight. Blotting primary antibodies against TrkB (cat. no. sc-12, Santa Cruz, USA) and GAPDH (cat. no. bs-2188R, Bioss, China) were used. The secondary antibodies conjugated to horseradish peroxidase (cat. no. bs-0295G-HRP, Bioss, China) were incubated with the PVDF membrane at room temperature for 2 hours. The ECL Western Blot Kit (cat. no. CW0049, CoWin Biosciences, China) was used to detect protein bands.

### 2.8. ChIP-qPCR (One-Step and Two-Step)

One-step ChIP experiments were performed according to a method described elsewhere [44]. At each condition, the cells were fixed using 1% formaldehyde at room temperature for 10 min. The cell lysate was sonicated three times for 10 s, and supernatants were collected and followed by immunoclearing with 2 ug sheared salmon sperm DNA, 20 ul preimmune serum, and 20 ul protein A-Sepharose (cat. no. 15918-014, Invitrogen, USA) for 2 hr at 4°C. Immunoprecipitation was performed overnight at 4°C with specific antibodies. After immunoprecipitation, 45°*μ*l protein A-Sepharose and 2 ug of salmon sperm DNA were added and the incubation was continued for another 1 hr. Precipitates were washed and extracted. Eluates were pooled and heated at 65°C for 6 hr to reverse the formaldehyde cross-linking. Antibodies against the RAR (cat. no. sc-773) and P300 (cat. no. sc-585) that were used for immunoprecipitation were purchased from Santa Cruz Biotechnology, USA. For p300, an additional two-step cross-linking ChIP assay [45] was performed, which, different from the ChIP procedure described above, includes a preliminary (before the formaldehyde fix) protein-protein cross-linking step with 2 mM di(*N*-succinimidyl)glutarate (cat. no. 80424, Sigma-Aldrich, USA) for 45 min at room temperature. ChIP products were subjected to real-time quantitative PCR using primers specific to the left, middle, and right parts of the potential binding region, and detailed primer sequences are shown in Table 2. Data from qPCR were expressed as percentage over input DNA.

### 2.9. Homologous Sequence Analysis

Homologous sequence analyses were performed to estimate the conservation of P300 peak regions (400 bps long, queried form ENCODE ChIP-seq data) and potential RA response regions (118 bps long, identified by ChIP-qPCR in the current study) across different species. Homologous sequences in genomes of different species were queried from the NCBI database via genome BLAST (https://blast.ncbi.nlm.nih.gov/Blast.cgi), by using the BLAST algorithm. The original sequences in *Homo sapiens* are shown in [Supplementary-material supplementary-material-1]. Homologous sequences were aligned by using the Clustalw module in the software MEGA7 [46], and molecular phylogenetic trees were constructed by using neighbour-joining algorithms and a maximum composite likelihood model in MEGA7. The position conservation of homologs was estimated by comparing the genome relative position of homologs and *NTRK2* genes in each species.

### 2.10. Statistical Analysis

All bioinformatics analyses were performed with the recommended parameters. The quantitative RT-qPCR and ChIP-qPCR data were statistically analysed by the two-sided Student's *t*-test with R (version 3.1.0) and are expressed as the mean ± standard deviation (SD) from at least three independent cell preparations. Values of *p* < 0.05 were considered significant.

## 3. Results

### 3.1. Characterization of SH-SY5Y-A Cells after Sequential Treatment with ATRA and BDNF

The morphological and neuronal characterization of ATRA and BDNF sequential treated SH-SY5Y-A cells was demonstrated by immunofluorescence detection of cytoskeleton protein beta-actin and classical neuronal marker protein MAP2. The normally cultured cells show both substrate-adherent (S-type) and neuroblastic (N-type) morphology (Figure 1(a)). After ATRA treatment for five days, the SH-SY5Y-A cells showed a change from an epithelial-like to a neuronal-like appearance with thin and clumped cell bodies and long projections (Figure 1(b)). After a subsequent BDNF treatment for three days (in the serum-free condition), the cells were contacted by projection and constructed a network-like structure (Figure 1(c)). As shown in Figures 1(d)–1(f), after a sequential ATRA and BDNF treatment, axon-like structures extended tens and hundreds of times longer than cell bodies, and adhesion and connection between cells can be universally observed. During the differentiation process, the expression level of MAP2 did not show significant changes; however, in differentiated cells, MAP2 proteins showed more aggregation (Figures 1(d)–1(f)) and formed vesicle-like structures (Figure 1(e)).

### 3.2. The Expression Signatures and Gene Regulatory Network of ATRA-Triggered Neuroblastoma Differentiation

As shown in Figure 2(a), genes that were differentially expressed in all three neuroblastoma cell lines were defined as neuroblastoma differentiation signatures. The DEG analysis of three neuroblastoma cell lines (SK-N-SH, SH-SY5Y-A, and SH-SY5Y-E) identified 2597, 2080, and 5698 differentially expressed probes (the top 100 differential expressed probes are shown in Tables [Supplementary-material supplementary-material-1]–[Supplementary-material supplementary-material-1]). Among them, 286 probes (representing 231 genes, as shown in [Supplementary-material supplementary-material-1]) were differentially expressed in all three cell lines, and the 231 genes were used as neuroblastoma differentiation signatures. All of the 231 genes were upregulated in differentiated neuroblastoma cells. The functional analysis showed that the 231 differentiation signatures are enriched in 14 interactive functional groups ([Supplementary-material supplementary-material-1]), including 111 functional GO terms ([Supplementary-material supplementary-material-1]), as shown in Figure 2(b). The 231 signature genes were further used to build the TF-target regulatory network in the three neuroblastoma cell lines, respectively. The information on regulons and MRs of three cell lines is shown in Tables [Supplementary-material supplementary-material-1]–[Supplementary-material supplementary-material-1]. The overlapping parts between three networks were obtained to form a common regulatory network of neuroblastoma differentiation. As shown in Figure 2(c), this common regulatory network was composed of 10 regulators and 71 target genes, which are listed in Table 3. After functional enrichment analysis, 15 of the 71 target genes that are significantly enriched in neuronal projection-related GO functional clusters (as shown in [Supplementary-material supplementary-material-1]) and 7 TFs, by which 15 genes are potentially regulated, were extracted to build a neuronal projection-related subnetwork (as shown in Figure 2(d)).

### 3.3. The Expression of Neuronal Developmental Genes in Serum Starvation and ATRA-Treated SH-SY5Y-A Cells

The mRNA levels of 19 genes (including 15 neuronal projection-related genes identified via the gene regulatory network analysis, 3 neuronal marker genes, and the cytoskeletal gene beta-actin) in six conditions were validated by RT-qPCR; the design of cell treatment and observation is shown in the top of Figure 3. As shown in Figure 3, the expression levels of five neuronal projection-related genes (*CNTN2*, *SNAP25*, *DNAH9*, *NFASC*, and *TTLL2*), two neuronal marker genes (*SYN1* and *SYP*), and the beta-actin gene were significantly different in the serum-free condition compared to the standard culture condition. Twelve neuronal projection-related genes (*ATP7A*, *SCN2A*, *CNTN2*, *SNAP25*, *BCL2*, *TM7SF2*, *RHBDL1*, *NFACS*, *RUFY3*, *TTLL3*, *CRB1*, and *NTRK2*), two neuron marker genes (*SYN1* and *SYP*), and the beta-actin gene were significantly differentially expressed in ATRA-positive and ATRA-negative conditions after 48 hours and/or six days of ATRA treatment. Most of the DEGs showed a fold change less than 3, except for the gene *NTRK2*, whose expression level was sharply increased, by more than a hundredfold, after ATRA treatment and rose continuously in the ATRA-positive condition.

### 3.4. *NTRK2* Overexpression Can Trigger the Differentiation of SH-SY5Y-A Cells

**Figure 5:**
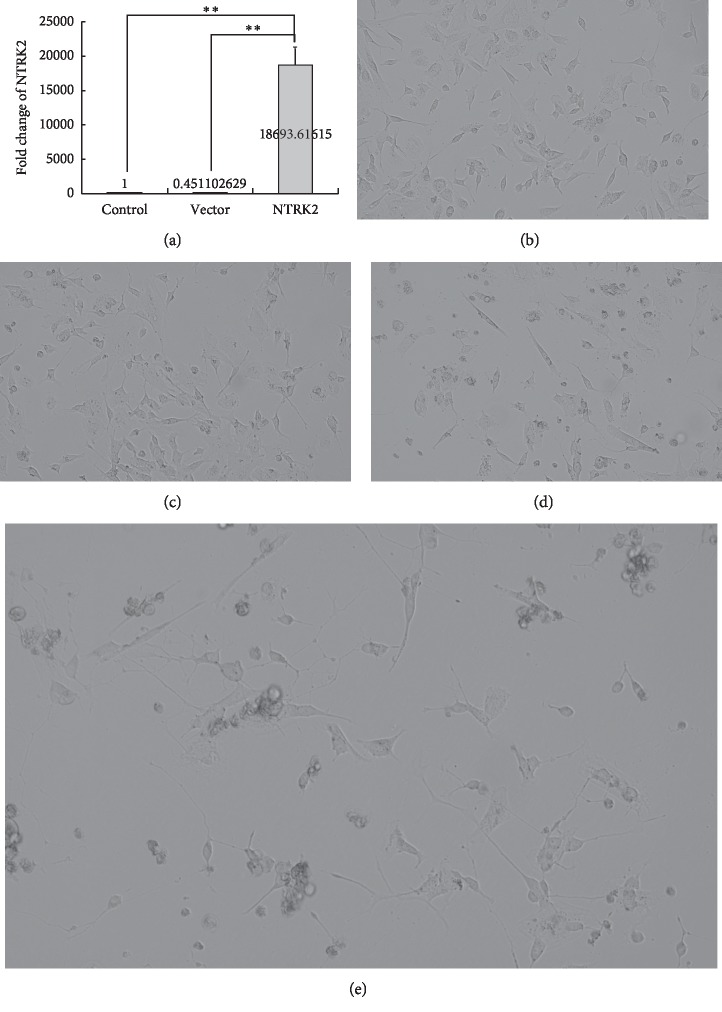
Effect of *NTRK2* overexpression on SH-SY5Y-A differentiation. The overexpression of the *NTRK2* gene caused differentiation-like changes in SH-SY5Y-A cells. (a) Expression levels of the *NTRK2* gene in different cell groups. Control: normally cultured SH-SY5Y-A cells. Vector: SH-SY5Y-A cells that were transfected with the empty pCMV6 plasmid (48hours after transfection). NTRK2: SH-SY5Y-A cells that were transfected with the full-length *NTRK2* cDNA expression plasmid (48hours after transfection). (b) Normally cultured SH-SY5Y-A cells. (c) SH-SY5Y-A cells that were transfected with the empty pCMV6 plasmid (48hours after transfection). (d) SH-SY5Y-A cells that were transfected with the full-length *NTRK2* cDNA expression plasmid (48 hours after transfection). (e) SH-SY5Y-A cells that were transfected with the full-length *NTRK2* cDNA expression plasmid for 48hours and subsequently treated with BDNF for 3 days. Phase contrast microscopy ×20. ^*∗∗*^*p* value<0.01.

The plasmids that express the full-length cDNA of the human *NTRK2* gene were transient transfected into SH-SY5Y-A cells, and the *NTRK2* gene was overexpressed in transfected cells (Figure 5(a)). As shown in Figure 5(d), two days after *NTRK2* transfection, similarly to ATRA treatment, the SH-SY5Y-A cells presented a differentiation-like morphology. The S-type cells were significantly decreased, the body of N-type cells became longer, and the length of axon-like structures was increased. After an additional BDNF treatment for 3 days, the intercellular junction structures were significantly observed (Figure 5(e)).

### 3.5. A Novel Region in the *NTRK2* Gene Responded Directly to ATRA Treatment

The expression changes of the *NTRK2* gene implied that it is regulated by ATRA directly. A transcript start site analysis and a P300 binding analysis were performed on the 5′ region of the *NTRK2* gene, as shown in Figure 4(a). There are eight *NTRK2* transcripts in 53 human tissues, according to data from GTEx. By using the long polyA RNA-seq assay, the ENCODE project identified no TSS in normally cultured SK-N-SH cells. But four TSSs regions were detected in ATRA-treated SK-N-SH cells, and their positions were corresponding to the first four exons of the *NTRK2* gene. A P300 binding region was detected in one of the two repeat ChIP-seq assays in ATRA-treated SK-N-SH cells but not in normally cultured cells. This P300 binding region locates less than 1 kb from all four TSS regions. A TFBS analysis on a 400 bps long region extending from this peak was performed by using three RAR binding matrixes (MA0159, PB0053, and PB0157, obtained from the database of JASPAR); the analysis results suggest there are three potential RAR binding site clusters (as shown in Figure 4(b)), and the potential TFBSs were predicted majorly to play a role as a negative regulator. To validate the direct regulation of the RAR on the *NTRK2* gene, normally cultured SH-SY5Y cells were treated with ATRA, the RAR agonist, and/or a well-characterized pan-RAR antagonist, AGN193109 [47]. As shown in Figure 4(c), compared with normally cultured cells, treatment with either ATRA or AGN193109 triggered the expression of TrkB protein, which is encoded by the *NTRK2* gene. To further identify the RAR binding region, three pairs of primers were designed for ChIP assays to estimate the RAR binding of left, middle, and right parts of the 400 bps long P300 peak region, as shown in Figure 4(d). By using a classical one-step ChIP assay, bindings between the RAR and all three regions were observed in normally cultured SH-SY5Y-A cells, and the binding level in the right region (118 bps long) was significantly higher than that in the other two regions (Figure 4(e)). Considering that P300 does not bind directly to DNA during RAR activation, the P300 binding on the right region was detected by both one-step and two-step ChIP assays after ATRA treatment. As shown in Figure 4(f), after 60 min of ATRA treatment, an increase of P300 binding was observed in a two-step ChIP assay.

### 3.6. Sequence Conservation and Position Specification of the Potential RA Response Region

**Figure 6:**
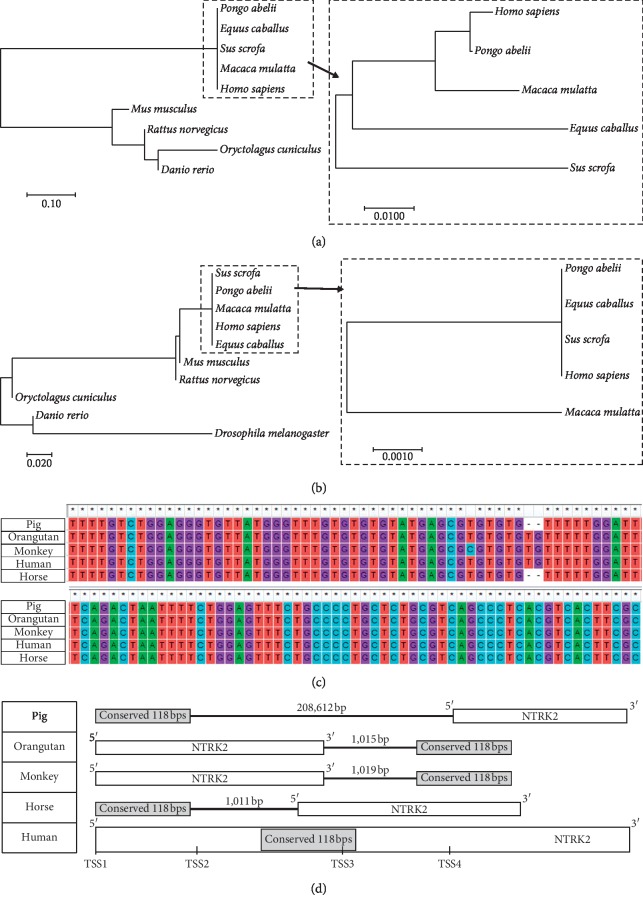
Conservation analyses of the potential RA response region. Functional elements are often sequence conserved across species. Homolog sequences of the P300 peak region (400 bps long) from ENCODE ChIP-seq data and the potential RAR binding region (118 bps long) identified by the current study across different species were analysed to construct phylogenetic trees. Besides sequence conservation, the relative position of the element and gene is a main factor in expression regulation. So the position analysis was performed on the most conserved sequence. (a) A phylogenetic tree of P300 peak region (400 bps long) homolog sequences in nine species. (b) A phylogenetic tree of the potential RAR binding region (118 bps long) homolog sequences in ten species. (c) Homolog sequences of the potential RAR binding region (118 bps long) in the five most conserved species. (d) The relative position of the conserved 118 bps long sequence and *NTRK2* gene in the five most conserved species.

Sequences homologous of the P300 binding peak region (400 bps long, queried form ENCODE ChIP-seq data) and the RA response region (118 bps long, identified by ChIP-qPCR in the current study) were queried against genomes of different species. The blast results are shown in Tables [Supplementary-material supplementary-material-1] and [Supplementary-material supplementary-material-1]. The molecular phylogenetic trees show that both the P300 binding peak region (400 bps long) and RA response region (118 bps long) sequences are conserved across species (Figures 6(a) and 6(b)), but the sequence difference of the 118 bps region is less. The consensus sequences of the 118 bps long region and its homologous sequences in the genomes of orangutan, monkey, horse, and pig are shown in Figure 6(c). Homologous sequences in the genomes of these four species are all located around the *NTRK2* gene, but their relative positions vary. As shown in Figure 6(d), the conserved sequence is located far away from the *NTRK2* gene in the pig genome; in the orangutan and monkey genomes, the conserved sequences are in the downstream region of *NTRK2*; in the horse genome, the conserved sequence is located in the upstream region of *NTRK2*; and only in the human genome, this sequence locates inside the *NTRK2* gene and is close to a TSS.

## 4. Discussion

As cell modules of neuronal differentiation, the differentiation of neuroblastoma cells triggered by ATRA has been widely reported; however, the molecular process underlying the induction is still unclear. Here, we revalidated this process and explored the underlying potential molecular processes. Our observations show that the single ATRA treatment could trigger the differentiation process of SH-SY5Y-A cells, and the subsequent BDNF treatment increased the degree of differentiation and made the differentiation more stable. These results are consistent with previous reports [48].

Nishida et al. [24], Korecka et al. [49], and Pezzini et al. [41] estimated the transcriptomic profiling of neuroblastoma using microarray or RNA-seq assays. To evaluate the common molecular processes in neuroblastoma differentiation, we remined the microarray data from Nishida et al. [24] that estimated expression profiling during the ATRA-induced differentiation of three different neuroblastoma cell lines (SK-N-SH, SH-SY5Y-A, and SH-SY5Y-E). The original study from Nishida et al. mainly focused on the PI3K-mediated signalling pathway (activated by ATRA) and TrkB-mediated signalling pathway (activated by BDNF) in SH-SY5Y cells. So the differentially expressed genes were compared between different cell lines, cells treated with the PI3K inhibitor or not, and cells treated with BDNF or not. In the current study, we recompared differentiated and undifferentiated expression profiling in each cell line and obtained the overlapping DEGs to reflect the common molecular processes in ATRA-triggered neuroblastoma differentiation. Our results show that the common changes focus on genes related to axon composition, nodes of Ranvier, transmembrane ion movement (both channel complexes and ATPase activity), and G-protein complex, in addition to genes encoding RA-responsive proteins. This suggests that ATRA influences a series of neuron differentiation-related processes, from morphological changes to ion transport and signal transduction. The affected genes were also involved in pathways associated with cerebellar cortex formation and eye photoreceptor cell differentiation, implying that RA plays an important role in organogenesis. Moreover, the pathway that is related to the multicellular organismal response to stress is also involved, supporting previous studies that reported a relationship between RA signalling and stress [50].

Besides identifying common neuroblastoma differentiation signatures, we further constructed a potential TF-target gene network focusing on these signatures. In the gene regulatory network of neuroblastoma differentiation, ten TF genes were identified as core regulators. The potential relationship between these TF genes and neuronal differentiation or neurodegenerative diseases has been reported in previous studies. The TFs encoded by *FOXJ3*, *HOXD1*, *MEIS2*, *NEF2L2*, *NFIB*, and *STAT5* have been reported to be involved in the mechanism of neuronal differentiation or contribute to maintain neuron function [50–56]. The TFs encoded by *STAT5A*, *MEIS2*, and *EFRRG* have been reported to be related to cognitive abilities [57–59]. The TF genes *FSOL2*, *MESI2*, *NEF2L2*, and *PRDM2* have been reported to be associated with neurodegenerative diseases [60–63]. Moreover, the TF genes *FSOL2*, *KLF11*, and *NEF2L2* play essential roles in the neurodegenerative diseases [64–66]. The analysis results in the current study suggested a potential cooperation manner of these TFs, which may provide evidence for further molecular mechanism research.

Subsequent to the bioinformatics analysis of public data, expression changes of neuronal differentiation-related genes were validated by RT-qPCR in the current study. Schneider et al. [67] and Encinas et al. [67] reported increases of some neuronal marker proteins (such as microtubule-associated protein 2 (MAP2)) in ATRA-treated neuroblastoma cells as a characteristic of differentiation, but in studies by Pezzini et al. [41] and Shipley et al. [22], the expression levels of these genes were not significantly changed after ATRA treatment. In our research, we cultured and treated SH-SY5Y-A cells to observe three neuronal marker genes: *MAP2* [68], *SYN1* [69], and *SYP* [70]. Considering that, in addition to ATRA treatment, serum starvation can also trigger neuroblastoma differentiation [71], we examined the influence of serum-free conditions too. Our results show that the expression levels of these three marker genes were more affected by serum than by ATRA and tended to decrease after ATRA treatment, consistent with the reports of Pezzini et al. [41] and Tong et al. [23]. Interestingly, although there was no significant change in abundance, MAP2 protein showed different intracellular localization after differentiation. The local enrichment in vesicle-like structures in differentiated neuroblastoma is consistent with that reported by Shipley et al. [22]. This implies that, beyond direct expression regulation, the ATRA treatment may also trigger a more complex molecular process that affects neuron markers.

The expression status of the 15 neuron projection-related genes in the differentiation-related regulatory network was also validated. In the original microarray data, the 15 genes were shown upregulated in the differentiation stage. Our results showed that the expression levels of most of the 15 neuronal projection genes including *BCL2* and *NFASC* that were reported from the original study by Nishida et al. were more strongly impacted by serum than by ATRA. This implies that their expression changes during differentiation may not be directly responsive to ATRA but are instead more likely to be follow-up changes.

Among the 15 genes, *NTRK2* is the only one whose expression level increased dramatically after ATRA treatment and continued to increase under ATRA conditions. This result is consistent with the study by Nishida et al. and suggests that *NTRK2* may be directly regulated by ATRA. The relationship between RA signalling and the regulation of *NTRK2* expression has been reported by many previous studies [72, 73]. Considering that *NTRK2* encodes TrkB, the BDNF receptor, this regulatory process may be essential for neuronal differentiation. The *NTRK2* gene has been reported to be associated with several psychiatric diseases, such as depression and bipolar disorder [25, 74, 75], and it has also been reported to be influenced by environmental factors such as stress [76, 77]. Therefore, the regulation of *NTRK2* may be important in neuronal differentiation and act as a key crosstalk point during disease-related gene-environment interactions. To validate the influence of *NTRK2* on neuron differentiation, we overexpressed this gene in SH-SY5Y-A cells by transfecting the full-length cDNA expression plasmid. Similarly to the effect of ATRA treatment, the overexpression of *NTRK2* triggered the cell differentiation, and this supports our hypothesis that ATRA influences neuroblastoma differentiation by regulating the expression of *NTRK2*.

The direct relationship between ATRA treatment and *NTRK2* gene expression in neuroblastoma was supported by TSS analysis in our study. Functional genomics data show that, after ATRA treatment, TSSs whose positions correspond to the first four exons of *NTKR2* genes appear. But the detailed mechanism by which ATRA regulates *NTRK2* has not been clearly reported. We hypothesized that ATRA regulates *NTRK2* in a classical manner in which ATRA binds with the RAR and then recruits P300 to trigger downstream epigenetic modifications. Functional genomics data from the ENCODE project and bioinformatics TFBS analysis suggested P300 may bind with RAR in the *NTRK2* promoter region after ATRA treatment. *NTRK2* may respond directly to ATRA after the activation of this region.

The RAR exerts active repressive functions in the absence of ligands, so the agonist which activates the RAR binds on DNA and the antagonist which inhibits the RAR's ability to bind to DNA may cause similar changes in expression of RAR-regulated genes [78]. In the current study, both the agonist and the antagonist of the RAR caused an increase of TrkB in SH-SY5Y-A cells, which confirms that the expression of the *NTRK2* gene is directly controlled by ATRA via the RAR in neuroblastoma cells. With the assumption that the RA response region may be located in the P300 binding peak mentioned above, we conducted ChIP assays in SH-SY5Y-A cells. A 118 bps long region showed significant bound with the RAR in normally cultured SH-SY5Y-A cells. So we deduced that the RA response sequence that directly binds to the RAR was inside this region. To measure the response to RA of this region, we subsequently used both one-step and two-step ChIP assays to test its binding with P300. After ATRA treatment for 60 min, the level of indirect P300 binding detected by the two-step ChIP assay was significantly increased in this region. This finding supports the hypothesis that ATRA activates the RAR in this region and recruited P300.

Functional noncoding DNA elements often maintain sequence conservation across different species [79]. The 118 bps long region we identified by ChIP-qPCR in the current study shows high sequence conservation among different species. This result further supports our hypothesis that it is a novel DNA element. Expect for sequence conservation, position conservation of the 118 bps long region was also analysed. This region is located near the *NTRK2* gene in the genomes of all five mammals we analysed, but the relative positions of the conserved region and *NTRK2* differ among these species; only in the human genome, this conserved sequence is located in the key promoter region. This implies that the DNA element may influence *NTRK2* expression in a human-specific manner.

In conclusion, we performed comprehensive analysis to explore the systematic molecular process underlying ATRA-triggered neuroblastoma differentiation. Differentiation signature genes were identified from public microarray data, and the systematic biological processes influenced by signature genes were analysed. The potential regulatory network related to differentiation signature genes was also obtained via our comprehensive bioinformatics analysis. These results provided multiple types of evidence for neuronal differentiation studies and neurodegenerative disease researches. The direct response of the *NTRK2* gene to ATRA treatment in SH-SY5Y-A cells we revealed in the current study suggested the important roles of this gene in ATRA-induced neuronal differentiation, and the novel RA response DNA element we identified in the *NTRK2* gene may be involved in the RA-related gene regulation in a human-specific manner.

## Figures and Tables

**Table 1:** Sequences of oligonucleotides used as primers for RT-qPCR.

Gene	Sequence	
*BCL2*	Forward	5′-TTGGCCCCCGTTGCTTTTCCTC-3′
	Reverse	5′-TCCCACTCGTAGCCCCTCTGCGAC-3′

*DNAH9*	Forward	5′-GAACTTGGAGAGGTGTGCCT-3′
	Reverse	5′-GGCCCTTGTGGTTTGTTTCTC-3′

*BBS9*	Forward	5′-ACCGATGTTGAGGTGGGAAC-3′
	Reverse	5′-GGATAACTCGCGGAATGCCA-3′

*TTLL3*	Forward	5′-TGATCCACGCACTTCAGACC-3′
	Reverse	5′-GAGCCGGATGCCCACATATT-3′

*CRB1*	Forward	5′-CCAGCAACACACCAGAGGAT-3′
	Reverse	5′-TTGAGAGGCACCTGGTGTTG-3′

*TM7SF2*	Forward	5′-CTGCCTCATCAATGGGCTTG-3′
	Reverse	5′-GATAGTTGGGATGGCGGACC-3′

*RHBDL1*	Forward	5′-CTCATCCAGGAGCAGCAG-3′
	Reverse	5′-TAGCAATGGCCCGCTTGAA-3′

*SCN2A*	Forward	5′-GCTTCCGCTTCTTTACCAGG-3′
	Reverse	5′-GCGTTCCTGTTTGGGTCTCT-3′

*NTRK2*	Forward	5′-CAATTGTGGTTTGCCATCTG-3′
	Reverse	5′-TGCAAAATGCACAGTGAGGT-3′

*NFASC*	Forward	5′-CGAGCCCTTGGAGGTTGATT-3′
	Reverse	5′-AGACTGAGGAGGCAGAGGAG-3′

*CNTN2*	Forward	5′-CACACCTCACCATCCTTCAGTC-3′
	Reverse	5′-CACATTTATCCTCTGCCCTTCC-3′

*SNAP25*	Forward	5′-GAACACAACCCTCCCGAGAA-3′
	Reverse	5′-GTTCATGCCTTCTTCGACACG-3′

*RUFY3*	Forward	5′-GAAAGTTTCGGTTCTGCCCG-3′
	Reverse	5′-TATGCAAGCTGGTGCTGTCA-3′

*MAP2*	Forward	5′-CAGTTTGGCTGAAGGTAGCTGAA-3′
	Reverse	5′-CACATCTGTGTGAGTGTGTGTGTGGA-3′

*SYN1*	Forward	5′-TGACCAATGCCTTCAACCTTC-3′
	Reverse	5′-AGTGGGGTATCAGTCGGAGAA-3′

*SYP*	Forward	5′-AGGTGCTGCAATGGGACTTT-3′
	Reverse	5′-GTTGAGTCCCGAGGTCACAG-3′

*ATP7A*	Forward	5′-TGGCAAGGCAGAAGTAAGGTATAA-3′
	Reverse	5′-ACGTCATTCCCCTCACAACAAG-3

*ARHGEF7*	Forward	5′-GCCTGGATAAATACCCTACGC-3′
	Reverse	5′-GGATGGCTTCCGTCAGGAT-3′

*Beta-actin*	Forward	5′-AGGCCAACCGCGAGAAGATGACC-3′
	Reverse	5′-GAAGTCCAGGGCGACGTAGCAC-3′

*GAPDH*	Forward	5′-TTCTTTTGCGTCGCCAGCCGA-3′
	Reverse	5′-GTGACCAGGCGCCCAATACGA-3′

**Table 2:** Sequences of oligonucleotides used as primers for ChIP-qPCR.

Region	Sequence	
Left	Forward	5′-CAGCCTCTACCGCGATTGT-3′
	Reverse	5′-CCTGGCCGTGTAGACATGC-3′

Middle	Forward	5′-ATGTCTACACGGCCAGGA-3′
	Reverse	5′-CCATAACACCCTCCAGACAAAAG-3′

Right	Forward	5′-TTTTGTCTGGAGGGTGTTATGGG-3′
	Reverse	5′-GCGAAGTGACGTGAGGGC-3′

**Table 3:** MRs and target genes in the regulatory network of neuroblastoma differentiation.

MRs	*ESRRG*, *FOSL2*, *FOXJ3*, *HOXD1*, *MEIS2*, *KLF11*, *NFE2L2*, *NFIB*, *PRDM2*, *STAT5A*
Target genes	*NFASC*, *LGALS12*, *TM7SF2*, *CRB1*, *PRCD*, *STAT5A*, *LGI1*, *ALS2CR8*, *SNAP25*, *ZNF189*, *CFLAR*, *GABARAPL1*, *ANKRD6*, *ADD3*, *CREBRF*, *ZBTB18*, *L3MBTL1*, *HBP1*, *DNAH9*, *GARNL3*, *RGS18*, *CREBL2*, *YPEL2*, *OGFOD1*, *DPP6*, *NTRK2*, *SCN2A*, *ZSCAN26*, *CDKN2AIP*, *ARHGEF7*, *CHEK1*, *RUFY3*, *BCL2*, *KLF11*, *ATP6AP1L*, *RRAGB*, *PRDM2*, *BAI3*, *CCDC146*, *SYN2*, *PELO*, *KIAA1377*, *RHBDL1*, *GNG3*, *ZNF211*, *ARPC4-TTLL3*, *ZCCHC12*, *SLC25A27*, *RGS13*, *CLIP3*, *RASL10B*, *C4orf6*, *RUNDC3B*, *PURG*, *MMP11*, *TGM2*, *ATP2B3*, *SLC35D3*, *TTLL3*, *RAB26*, *ZNF555*, *CACNA1B*, *LINC00086*, *ZFP2*, *ATP7A*, *ATRNL1*, *ZNF625*, *BBS9*, *PCBP1-AS1*, *CNTN2*, *RFPL1S*

## Data Availability

The data used to support the findings of this study are available from the corresponding author upon request.

## Supplementary Materials

Supplementary Materials Table S1: samples in dataset GSE9169. Table S2: 823 TFs and their probes in the Affy_HG-U133_plus_2 microarray platform. Table S3: original sequences in *Homo sapiens* for homologous sequence analysis. Table S4: top 100 DEGs between differentiated and undifferentiated SH-SY5Y-A cells. Table S5: top 100 DEGs between differentiated and undifferentiated SH-SY5Y-E cells. Table S6: top 100 DEGs between differentiated and undifferentiated SK-N-SH cells. Table S7: overlapped DEGs in three neuroblastoma cell lines. Table S8: functional groups enriched by neuroblastoma differentiation signature genes. Table S9: GO terms enriched by neuroblastoma differentiation signature genes. Table S10: numbers of regulons, signature regulons, and master regulators in three neuroblastoma cell lines. Table S11: master regulators that controlled more than 100 neuroblastoma differentiation signatures. Table S12: the core MRs and their controlled signatures during neuroblastoma differentiation. Table S13: GO clusters enriched by genes in the regulatory network. Table S14: homologous sequences of the 400 bps long region in 8 species. Table S15: homologous sequences of the 118 bps long region in 9 species.
