# Supplementary material for: A Systematic Analysis Revealed the Potential Gene Regulatory Processes of ATRA-Triggered Neuroblastoma Differentiation and Identified a Novel RA Response Sequence in the NTRK2 Gene
Source: Biomed Res Int. 2020 Feb 19;2020:6734048. doi: 10.1155/2020/6734048 (PMC7053487; doi:10.1155/2020/6734048)
Supplement: Supplementary Materials — Table S1: samples in dataset GSE9169. Table S2: 823 TFs and their probes in the Affy_HG-U133_plus_2 microarray platform. Table S3: original sequences in Homo sapiens for homologous sequence analysis. Table S4: top 100 DEGs between differentiated and undifferentiated SH-SY5Y-A cells. Table S5: top 100 DEGs between differentiated and undifferentiated SH-SY5Y-E cells. Table S6: top 100 DEGs between differentiated and undifferentiated SK-N-SH cells. Table S7: overlapped DEGs in three neuroblastoma cell lines. Table S8: functional groups enriched by neuroblastoma differentiation signature genes. Table S9: GO terms enriched by neuroblastoma differentiation signature genes. Table S10: numbers of regulons, signature regulons, and master regulators in three neuroblastoma cell lines. Table S11: master regulators that controlled more than 100 neuroblastoma differentiation signatures. Table S12: the core MRs and their controlled signatures during neuroblastoma differentiation. Table S13: GO clusters enriched by genes in the regulatory network. Table S14: homologous sequences of the 400 bps long region in 8 species. Table S15: homologous sequences of the 118 bps long region in 9 species. [file 6734048.f1.pdf]

Title: A systematic analysis revealed the potential gene regulatory processes of ATRA triggered neuroblastoma differentiation and identified a novel RA response sequence in NTRK2 gene

Liyuan Guo[1, 2], Wei Lin[1, 2], Yidan Zhang[1, 2], and Jing Wang[1, 2‡]

[1]CAS Key Laboratory of Mental Health, Institute of Psychology, Chinese Academy of Sciences, Beijing 100101, China and [2]Department of Psychology, University of Chinese Academy of Sciences, Beijing 100049, China

[‡]Correspondence:

1. Jing Wang, Ph.D., CAS Key Laboratory of Mental Health, Institute of Psychology, Chinese Academy of Sciences, 16 Lincui Road, Chaoyang District, Beijing, 100101, China.

Email: wangjing@psych.ac.cn

Phone: 86-10-64855841

Fax: 86-10-64855841

Supplementary Tables

Table S1 Samples in dataset GSE9169.

Table S2 823 TFs and their probes in Affy_HG-U133_plus_2 microarray platform

Table S3 Original sequences in Homo sapiens for Homologous sequence analysis

Table S4 Top 100 DEGs between differentiated and un-differentiated SH-SY5Y-A cells

Table S5 Top 100 DEGs between differentiated and un-differentiated SH-SY5Y-E cells

Table S6 Top 100 DEGs between differentiated and un-differentiated SK-N-SH cells

Table S7 Overlapped DEGs in three neuroblastoma cell lines

Table S8 Functional groups enriched by neuroblastoma differentiation signature genes.

Table S9 GO terms enriched by neuroblastoma differentiation signature genes.

Table S10 Numbers of regulons, signature-regulons, and master regulators in three neuroblastoma cell lines.

Table S11 Master regulators that controlled more than 100 Neuroblastoma differentiation signatures.

Table S12 The core MRs and their controlled signatures during neuroblastoma differentiation

Table S13 GO clusters that enriched by genes in regulatory network

Table S14 Homologous sequences of the 400bps long region in 8 species

Table S15 Homologous sequences of the 118 bps long region in 9 species

Table S1 Samples in dataset GSE9169.

No.	Sample ID	Treatment	Type in current study
1	GSM231608	SH-SY5Y(ATCC)_RA_inhibiter_LY294002_0h_rep1	Un-differentiated SH-SY5Y-A
2	GSM231609	SH-SY5Y(ATCC)_RA_inhibiter_LY294002_6h_rep1	Not used
3	GSM231610	SH-SY5Y(ATCC)_RA_inhibiter_LY294002_1d_rep1	Not used
4	GSM231611	SH-SY5Y(ATCC)_RA_inhibiter_LY294002_2d_rep1	Not used
5	GSM231612	SH-SY5Y(ATCC)_RA_inhibiter_LY294002_3d_rep1	Not used
6	GSM231613	SH-SY5Y(ATCC)_RA_inhibiter_LY294002_5d_rep1	Not used
7	GSM231614	SH-SY5Y(ATCC)_RA_inhibiter_LY294002_0h_rep2	Un-differentiated SH-SY5Y-A
8	GSM231615	SH-SY5Y(ATCC)_RA_inhibiter_LY294002_6h_rep2	Not used
9	GSM231616	SH-SY5Y(ATCC)_RA_inhibiter_LY294002_1d_rep2	Not used
10	GSM231617	SH-SY5Y(ATCC)_RA_inhibiter_LY294002_2d_rep2	Not used
11	GSM231618	SH-SY5Y(ATCC)_RA_inhibiter_LY294002_3d_rep2	Not used
12	GSM231619	SH-SY5Y(ATCC)_RA_inhibiter_LY294002_5d_rep2	Not used
13	GSM231620	SH-SY5Y(ATCC)_RA_0h_rep1	Un-differentiated SH-SY5Y-A
14	GSM231621	SH-SY5Y(ATCC)_RA_6h_rep1	Differentiated SH-SY5Y-A
15	GSM231622	SH-SY5Y(ATCC)_RA_1d_rep1	Not used
16	GSM231623	SH-SY5Y(ATCC)_RA_2d_rep1	Not used
17	GSM231624	SH-SY5Y(ATCC)_RA_3d_rep1	Not used
18	GSM231625	SH-SY5Y(ATCC)_RA_5d_rep1	Not used
19	GSM231626	SH-SY5Y(ATCC)_RA_0h_rep2	Un-differentiated SH-SY5Y-A
20	GSM231627	SH-SY5Y(ATCC)_RA_6h_rep2	Differentiated SH-SY5Y-A
21	GSM231628	SH-SY5Y(ATCC)_RA_1d_rep2	Not used
22	GSM231629	SH-SY5Y(ATCC)_RA_2d_rep2	Not used
23	GSM231630	SH-SY5Y(ATCC)_RA_3d_rep2	Not used
24	GSM231631	SH-SY5Y(ATCC)_RA_5d_rep2	Not used
25	GSM231632	SH-SY5Y(ECACC)_RA_inhibiter_LY294002_0h_rep1	Un-differentiated SH-SY5Y-E
26	GSM231633	SH-SY5Y(ECACC)_RA_inhibiter_LY294002_6h_rep1	Not used
27	GSM231634	SH-SY5Y(ECACC)_RA_inhibiter_LY294002_1d_rep1	Not used
28	GSM231635	SH-SY5Y(ECACC)_RA_inhibiter_LY294002_2d_rep1	Not used
29	GSM231636	SH-SY5Y(ECACC)_RA_inhibiter_LY294002_3d_rep1	Not used
30	GSM231637	SH-SY5Y(ECACC)_RA_inhibiter_LY294002_5d_rep1	Not used
31	GSM231638	SH-SY5Y(ECACC)_RA_inhibiter_LY294002_0h_rep2	Un-differentiated SH-SY5Y-E
32	GSM231639	SH-SY5Y(ECACC)_RA_inhibiter_LY294002_6h_rep2	Not used
33	GSM231640	SH-SY5Y(ECACC)_RA_inhibiter_LY294002_1d_rep2	Not used
34	GSM231641	SH-SY5Y(ECACC)_RA_inhibiter_LY294002_2d_rep2	Not used
35	GSM231642	SH-SY5Y(ECACC)_RA_inhibiter_LY294002_3d_rep2	Not used
36	GSM231643	SH-SY5Y(ECACC)_RA_inhibiter_LY294002_5d_rep2	Not used
37	GSM231644	SH-SY5Y(ECACC)_RA_0h_rep1	Un-differentiated SH-SY5Y-E
38	GSM231645	SH-SY5Y(ECACC)_RA_6h_rep1	Not used
39	GSM231646	SH-SY5Y(ECACC)_RA_1d_rep1	Not used
40	GSM231647	SH-SY5Y(ECACC)_RA_2d_rep1	Not used
41	GSM231648	SH-SY5Y(ECACC)_RA_3d_rep1	Not used
42	GSM231649	SH-SY5Y(ECACC)_RA_5d_rep1	Not used
43	GSM231650	SH-SY5Y(ECACC)_BDNF_6h_rep1	Not used
44	GSM231651	SH-SY5Y(ECACC)_BDNF_1d_rep1	Not used
45	GSM231652	SH-SY5Y(ECACC)_BDNF_2d_rep1	Not used
46	GSM231653	SH-SY5Y(ECACC)_BDNF_3d_rep1	Differentiated SH-SY5Y-E
47	GSM231654	SH-SY5Y(ECACC)_RA_0h_rep2	Un-differentiated SH-SY5Y-E
48	GSM231655	SH-SY5Y(ECACC)_RA_6h_rep2	Not used

(Continued)

No.	Sample ID	Treatment	Type in current study
49	GSM231656	SH-SY5Y(ECACC)_RA_1d_rep2	Not used
50	GSM231657	SH-SY5Y(ECACC)_RA_2d_rep2	Not used
51	GSM231658	SH-SY5Y(ECACC)_RA_3d_rep2	Not used
52	GSM231659	SH-SY5Y(ECACC)_RA_5d_rep2	Not used
53	GSM231660	SH-SY5Y(ECACC)_BDNF_6h_rep2	Not used
54	GSM231661	SH-SY5Y(ECACC)_BDNF_1d_rep2	Not used
55	GSM231662	SH-SY5Y(ECACC)_BDNF_2d_rep2	Not used
56	GSM231663	SH-SY5Y(ECACC)_BDNF_3d_rep2	Differentiated SH-SY5Y-E
57	GSM231664	SH-SY5Y(ECACC)_RA_0h_rep3	Un-differentiated SH-SY5Y-E
58	GSM231665	SH-SY5Y(ECACC)_RA_6h_rep3	Not used
59	GSM231666	SH-SY5Y(ECACC)_RA_1d_rep3	Not used
60	GSM231667	SH-SY5Y(ECACC)_RA_2d_rep3	Not used
61	GSM231668	SH-SY5Y(ECACC)_RA_3d_rep3	Not used
62	GSM231669	SH-SY5Y(ECACC)_RA_5d_rep3	Not used
63	GSM231670	SH-SY5Y(ECACC)_BDNF_6h_rep3	Not used
64	GSM231671	SH-SY5Y(ECACC)_BDNF_1d_rep3	Not used
65	GSM231672	SH-SY5Y(ECACC)_BDNF_2d_rep3	Not used
66	GSM231673	SH-SY5Y(ECACC)_BDNF_3d_rep3	Differentiated SH-SY5Y-E
67	GSM231674	SK-N-SH_RA_0h_rep1	Un-differentiated SK-N-SH
68	GSM231675	SK-N-SH_RA_6h_rep1	Not used
69	GSM231676	SK-N-SH_RA_1d_rep1	Not used
70	GSM231677	SK-N-SH_RA_2d_rep1	Not used
71	GSM231678	SK-N-SH_RA_3d_rep1	Not used
72	GSM231679	SK-N-SH_RA_5d_rep1	Not used
73	GSM231680	SK-N-SH_BDNF_6h_rep1	Not used
74	GSM231681	SK-N-SH_BDNF_1d_rep1	Not used
75	GSM231682	SK-N-SH_BDNF_2d_rep1	Not used
76	GSM231683	SK-N-SH_BDNF_3d_rep1	Differentiated SK-N-SH
77	GSM231684	SK-N-SH_RA_0h_rep2	Un-differentiated SK-N-SH
78	GSM231685	SK-N-SH_RA_6h_rep2	Not used
79	GSM231686	SK-N-SH_RA_1d_rep2	Not used
80	GSM231687	SK-N-SH_RA_2d_rep2	Not used
81	GSM231688	SK-N-SH_RA_3d_rep2	Not used
82	GSM231689	SK-N-SH_RA_5d_rep2	Not used
83	GSM231690	SK-N-SH_BDNF_6h_rep2	Not used
84	GSM231691	SK-N-SH_BDNF_1d_rep2	Not used
85	GSM231692	SK-N-SH_BDNF_2d_rep2	Not used
86	GSM231693	SK-N-SH_BDNF_3d_rep2	Differentiated SK-N-SH

Table S2~~1~~ 823 TFs and their probes in Affy_HG-U133_plus_2 microarray platform.

TF_name	Probes	TF_name	Probes	TF_name	Probes	TF_name	Probes	TF_name	Probes	TF_name	Probes
AATF	209165_at	ASCL1	209988_s_at	BLZF1	32088_at	CLOCK	204980_at	CTBP2	201218_at	EDF1	209058_at
ADNP	201773_at	ASCL1	213768_s_at	BNC1	1552487_a_at	CLOCK	227531_at	CTBP2	201219_at	EDF1	209059_s_at
ADNP	226426_at	ASCL2	207607_at	BNC1	206581_at	CLOCK	217563_at	CTBP2	201220_x_at	EGR1	201693_s_at
AEBP1	201792_at	ASCL2	229215_at	BRD8	202227_s_at	CLOCK	225856_at	CTBP2	210554_s_at	EGR1	201694_s_at
AFF1	201924_at	ATF1	1558233_s_at	BRD8	210352_at	CNBP	206158_s_at	CTBP2	210835_s_at	EGR1	227404_s_at
AFF1	211826_s_at	ATF1	1565269_s_at	BRD8	242265_at	CNBP	227731_at	CTBP2	215377_at	EGR2	205249_at
AFF1	215451_s_at	ATF1	222103_at	BRF1	1552399_a_at	CNOT7	1552344_s_at	CTCF	202521_at	EGR3	206115_at
AFF3	1565034_s_at	ATF2	1555146_at	BRF1	203754_s_at	CNOT7	218250_s_at	CTNNB1	1554411_at	EGR4	207767_s_at
AFF3	205734_s_at	ATF2	205446_s_at	BRF1	215676_at	CNOT7	225053_at	CTNNB1	201533_at	EGR4	207768_at
AFF3	205735_s_at	ATF2	212984_at	BRF1	215677_s_at	CNOT7	233019_at	CTNNB1	223679_at	EGR4	231575_at
AFF3	227198_at	ATF3	1554420_at	BRPF1	204481_at	CNOT8	202162_s_at	DAXX	201763_s_at	ELF1	212418_at
AFF3	242078_at	ATF3	1554980_a_at	BTAF1	209430_at	CNOT8	202163_s_at	DAXX	216038_x_at	ELF1	212420_at
AFF3	243967_at	ATF3	202672_s_at	BUD31	205690_s_at	CNOT8	202164_s_at	DBP	209782_s_at	ELF2	203822_s_at
AFF4	1555435_at	ATF4	200779_at	BUD31	215815_at	CREB1	204312_x_at	DBP	209783_at	ELF2	210361_s_at
AFF4	1555436_a_at	ATF5	204998_s_at	CBFA2T2	1554637_a_at	CREB1	204313_s_at	DDIT3	209383_at	ELF2	242735_x_at
AFF4	219199_at	ATF5	204999_s_at	CBFA2T2	207625_s_at	CREB1	204314_s_at	DEK	200934_at	ELF3	201003_x_at
AFF4	225229_at	ATF5	230938_x_at	CBFA2T2	209144_s_at	CREB1	214513_s_at	DENND4A	1554352_s_at	ELF3	201510_at
AFF4	232864_s_at	ATF5	217389_s_at	CBFA2T2	209145_s_at	CREB1	225565_at	DENND4A	214787_at	ELF3	208270_s_at
AFF4	232865_at	ATF6	203952_at	CBFA2T2	233243_at	CREB1	225572_at	DLX2	207147_at	ELF3	210827_s_at
AFF4	239439_at	ATF6	217550_at	CBFA2T2	238549_at	CREB1	237289_at	DLX2	215685_s_at	ELF3	229842_at
AFF4	243487_at	ATF6	226941_at	CBFA2T3	208056_s_at	CREB3	209432_s_at	DLX4	208216_at	ELF4	203490_at
AFF4	244628_at	ATF6	231927_at	CBFB	202370_s_at	CREB3L1	213059_at	DLX4	210259_s_at	ELF4	31845_at
AHCTF1	1560224_at	ATF7	206402_s_at	CBFB	206788_s_at	CREB3L1	213498_at	DLX5	213707_s_at	ELF5	220624_s_at
AHCTF1	214766_s_at	ATF7	206684_s_at	CBL	1568191_at	CREB3L2	212345_s_at	DLX6	221289_at	ELF5	220625_s_at
AHCTF1	226115_at	ATF7	214184_at	CBL	206607_at	CREB3L2	228759_at	DLX6	239309_at	ELK1	203617_x_at
AHR	202820_at	ATF7	228829_at	CBL	225231_at	CREB3L2	237819_at	DLX6	242940_x_at	ELK1	210376_x_at
ALX4	208330_at	ATF7	228830_s_at	CBL	225234_at	CREB5	205931_s_at	DMTF1	203301_s_at	ELK1	210850_s_at
AR	211110_s_at	ATF7	235160_at	CBL	229010_at	CREB5	229228_at	DR1	1558236_at	ELK3	206127_at
AR	226192_at	ATF7	244587_at	CBL	243475_at	CREB5	232555_at	DR1	1558237_x_at	ELK3	221773_at
AR	226197_at	ATOH1	221336_at	CCRN4L	1554283_at	CREB5	242329_at	DR1	207654_x_at	ELK4	205994_at
AR	211621_at	BACH1	1552401_a_at	CCRN4L	220671_at	CREBBP	202160_at	DR1	209187_at	ELK4	206919_at
ARID3A	205865_at	BACH1	1553608_a_at	CDX1	206430_at	CREBBP	211808_s_at	DR1	209188_x_at	ELK4	214831_at
ARID3A	228008_at	BACH1	204194_at	CDX2	206387_at	CREBBP	228177_at	DR1	216652_s_at	ELK4	225159_s_at
ARID4A	205062_x_at	BACH1	210818_s_at	CDX2	231606_at	CREBBP	235858_at	DR1	216653_at	ELK4	230549_at
ARID4A	230141_at	BACH1	234315_at	CDX4	221340_at	CREBL2	201988_s_at	DR1	235585_at	ELK4	238761_at
ARNT	210828_s_at	BACH1	234938_at	CEBPA	204039_at	CREBL2	201989_s_at	DRAP1	203258_at	EMX1	215264_at
ARNT	218221_at	BACH2	221234_s_at	CEBPB	212501_at	CREBL2	201990_s_at	DUX1	208176_at	EMX1	215265_at
ARNT	218222_x_at	BACH2	227173_s_at	CEBPD	203973_s_at	CREG1	201200_at	E2F1	2028_s_at	EMX2	221950_at
ARNT	230619_at	BARX2	210419_at	CEBPD	213006_at	CREM	207630_s_at	E2F1	204947_at	EN1	220559_at
ARNT	231016_s_at	BATF	205965_at	CEBPE	214523_at	CREM	209967_s_at	E2F2	207042_at	EN2	207060_at
ARNT	233724_at	BAZ1B	208445_s_at	CEBPG	204203_at	CREM	210171_s_at	E2F2	228361_at	ENO1	201231_s_at
ARNT2	202986_at	BAZ1B	213336_at	CEBPG	225527_at	CREM	214508_x_at	E2F2	235582_at	ENO1	217294_s_at
ARNTL	209824_s_at	BAZ1B	211313_s_at	CEBPZ	203341_at	CREM	228092_at	E2F3	203692_s_at	ENO1	240258_at
ARNTL	210971_s_at	BAZ1B	229658_at	CIITA	205101_at	CREM	230511_at	E2F3	203693_s_at	ENO1	216554_s_at
ARNTL2	220658_s_at	BCL6	203140_at	CIITA	210925_at	CRX	217510_at	E2F4	202248_at	EP300	202221_s_at
ARNTL2	223586_at	BCL6	215990_s_at	CIITA	211884_s_at	CRX	231742_at	E2F4	38707_r_at	EP300	213579_s_at
ARNTL2	224204_x_at	BCL6	228758_at	CITED1	207144_s_at	CTBP1	1557714_at	E2F5	221586_s_at	EPAS1	200878_at
ARNTL2	1563101_at	BLZF1	1558560_s_at	CITED2	207980_s_at	CTBP1	203392_s_at	E2F6	203957_at	EPAS1	200879_s_at
ASCL1	209985_s_at	BLZF1	203840_at	CITED2	209357_at	CTBP1	212863_x_at	E2F8	219990_at	ERCC6	207347_at
ASCL1	209987_s_at	BLZF1	210462_at	CITED2	227287_at	CTBP1	213980_s_at	E4F1	218524_at	ERCC6	230108_at

(Continued)

TF_name	Probes	TF_name	Probes	TF_name	Probes	TF_name	Probes	TF_name	Probes	TF_name	Probes
ERF	203643_at	ETV6	235056_at	FOXJ2	1555305_at	GATAD1	208503_s_at	HIF3A	1556069_s_at	HNF4A	230914_at
ERF	230368_at	ETV6	239740_at	FOXJ2	203734_at	GATAD1	213018_at	HIF3A	219319_at	HNF4A	214851_at
ERG	211626_x_at	ETV7	221680_s_at	FOXJ3	206015_s_at	GATAD1	214718_at	HIF3A	222123_s_at	HNF4G	207456_at
ERG	213541_s_at	ETV7	224225_s_at	FOXJ3	217310_s_at	GATAD1	242634_at	HIF3A	222124_at	HNF4G	232271_at
ERG	222079_at	EVX1	207914_x_at	FOXK2	203064_s_at	GATAD2A	218131_s_at	HIF3A	233517_s_at	HOXA1	214639_s_at
ERG	241926_s_at	EVX1	231706_s_at	FOXK2	226224_at	GATAD2A	222526_at	HIF3A	232669_at	HOXA10	213147_at
ESR1	205225_at	EWSR1	209214_s_at	FOXK2	242938_s_at	GATAD2A	234294_x_at	HIRA	217427_s_at	HOXA10	213150_at
ESR1	211233_x_at	EWSR1	210011_s_at	FOXK2	242937_at	GATAD2A	238324_at	HIRA	1569560_at	HOXA11	208493_at
ESR1	211234_x_at	EWSR1	210012_s_at	FOXL1	216572_at	GATAD2A	229166_s_at	HIVEP1	1560485_at	HOXA11	213823_at
ESR1	211235_s_at	EWSR1	229966_at	FOXL1	243409_at	GATAD2A	236932_s_at	HIVEP1	204512_at	HOXA2	214457_at
ESR1	215551_at	FEV	207260_at	FOXM1	202580_x_at	GBX2	210560_at	HIVEP2	212641_at	HOXA3	208604_s_at
ESR1	215552_s_at	FEZF2	221086_s_at	FOXN1	1558687_a_at	GLI2	207034_s_at	HIVEP2	212642_s_at	HOXA3	235521_at
ESR1	217163_at	FEZF2	233972_s_at	FOXN1	207683_at	GLI2	208057_s_at	HIVEP2	243254_at	HOXA4	206289_at
ESR1	217190_x_at	FEZF2	233897_at	FOXP1	223287_s_at	GLI2	228537_at	HIVEP3	220042_x_at	HOXA5	213844_at
ESR1	211627_x_at	FLI1	204236_at	FOXP1	223936_s_at	GLI3	1569342_at	HIVEP3	232581_x_at	HOXA6	208557_at
ESR2	210780_at	FLI1	210786_s_at	FOXP1	223937_at	GLI3	205201_at	HIVEP3	235122_at	HOXA7	206847_s_at
ESR2	211117_x_at	FLI1	211825_s_at	FOXP1	224837_at	GLI3	227376_at	HIVEP3	244764_at	HOXA7	235753_at
ESR2	211118_x_at	FLI1	234614_at	FOXP1	224838_at	GMEB1	220938_s_at	HIVEP3	233884_at	HOXA9	209905_at
ESR2	211119_at	FLI1	237722_at	FOXP1	229844_at	GMEB1	230613_at	HLF	204753_s_at	HOXA9	214651_s_at
ESR2	211120_x_at	FMNL2	226184_at	FOXP1	1558996_at	GMEB1	235232_at	HLF	204754_at	HOXB13	209844_at
ESRRA	1487_at	FMNL2	242665_at	FOXP1	235444_at	GMEB1	235233_s_at	HLF	204755_x_at	HOXB13	230105_at
ESRRA	203193_at	FMNL2	235881_at	FOXP3	221333_at	GTF2IRD1	218412_s_at	HMBOX1	219269_at	HOXB2	205453_at
ESRRB	1556156_at	FOS	209189_at	FOXP3	221334_s_at	HAND1	220138_at	HMBOX1	225504_at	HOXB5	205600_x_at
ESRRB	223858_at	FOSB	202768_at	FOXP3	224211_at	HAND2	220480_at	HMG20A	218152_at	HOXB5	205601_s_at
ESRRB	207726_at	FOSL1	204420_at	FUBP1	203091_at	HBP1	207361_at	HMG20B	209113_s_at	HOXB6	205366_s_at
ESRRG	207981_s_at	FOSL2	205409_at	FUBP1	214093_s_at	HBP1	209102_s_at	HMG20B	210719_s_at	HOXB6	205365_at
ESRRG	209966_x_at	FOSL2	218880_at	FUBP1	214094_at	HBP1	236645_at	HMG20B	213966_at	HOXB7	204778_x_at
ETS1	1555355_a_at	FOSL2	218881_s_at	FUBP3	212824_at	HCFC1	202473_x_at	HMGA1	206074_s_at	HOXB7	204779_s_at
ETS1	214447_at	FOSL2	225262_at	FUBP3	239193_at	HCFC1	202474_s_at	HMGA1	210457_x_at	HOXB7	216973_s_at
ETS1	224833_at	FOSL2	228188_at	GABPB2	228466_at	HCFC1	231177_at	HMGA2	1558682_at	HOXB7	231250_at
ETS2	201328_at	FOXA1	237086_at	GAS7	202191_s_at	HCLS1	202957_at	HMGA2	1558683_a_at	HOXB7	243441_at
ETS2	201329_s_at	FOXA1	204667_at	GAS7	202192_s_at	HES1	203393_at	HMGA2	1561633_at	HOXB8	221278_at
ETS2	241193_at	FOXA2	210103_s_at	GAS7	207704_s_at	HES1	203394_s_at	HMGA2	1567224_at	HOXB8	229667_s_at
ETV1	206501_x_at	FOXA2	40284_at	GAS7	210872_x_at	HES1	203395_s_at	HMGA2	1568287_at	HOXB9	216417_x_at
ETV1	217053_x_at	FOXA2	214312_at	GAS7	211067_s_at	HES2	214521_at	HMGA2	208025_s_at	HOXB9	226461_at
ETV1	217061_s_at	FOXB1	208513_at	GATA1	1555590_a_at	HES2	216674_at	HMGA2	1559891_at	HOXC10	218959_at
ETV1	221910_at	FOXD1	206307_s_at	GATA1	210446_at	HES2	231928_at	HMGA2	1567223_at	HOXC11	206745_at
ETV1	221911_at	FOXD3	208500_x_at	GATA2	209710_at	HESX1	211267_at	HMGA2	1568286_at	HOXC4	206194_at
ETV3	1552423_at	FOXD3	241612_at	GATA2	210358_x_at	HEY1	218839_at	HMGB1	200679_x_at	HOXC5	206739_at
ETV3	214480_at	FOXD3	241609_at	GATA2	207954_at	HEY1	44783_s_at	HMGB1	200680_x_at	HOXC6	206858_s_at
ETV3	227200_at	FOXE1	206912_at	GATA3	209602_s_at	HEY2	219743_at	HMGB1	214938_x_at	HOXD1	205975_s_at
ETV4	1554576_a_at	FOXE1	231331_at	GATA3	209603_at	HEY2	222921_s_at	HMGB1	224731_at	HOXD1	205974_at
ETV4	211603_s_at	FOXE1	208239_at	GATA3	209604_s_at	HEYL	220662_s_at	HMGB1	224734_at	HOXD10	207373_at
ETV5	203348_s_at	FOXE3	220621_at	GATA4	1553131_a_at	HEYL	226828_s_at	HMGB2	208808_s_at	HOXD10	229400_at
ETV5	203349_s_at	FOXF1	205935_at	GATA4	1570276_a_at	HHEX	204689_at	HMGB2	236091_at	HOXD10	238847_at
ETV5	216375_s_at	FOXF2	206377_at	GATA4	205517_at	HHEX	215933_s_at	HMX1	207353_s_at	HOXD11	214604_at
ETV5	230102_at	FOXH1	207644_at	GATA4	230855_at	HIC1	208461_at	HNF4A	208429_x_at	HOXD12	221411_at
ETV5	231083_at	FOXH1	231407_s_at	GATA4	243692_at	HIC1	230218_at	HNF4A	214832_at	HOXD13	207397_s_at
ETV6	205585_at	FOXI1	208006_at	GATA6	210002_at	HIF1A	200989_at	HNF4A	216889_s_at	HOXD13	207398_at
ETV6	225764_at	FOXJ1	205906_at	GATA6	229282_at	HIF3A	1555318_at	HNF4A	230772_at	HOXD13	236681_at

(Continued)

TF_name	Probes	TF_name	Probes	TF_name	Probes	TF_name	Probes	TF_name	Probes	TF_name	Probes
HOXD3	1552337_s_at	IRX5	210239_at	KLF9	203541_s_at	MAX	209331_s_at	MLX	217909_s_at	MYT1	210341_at
HOXD3	206601_s_at	ISL1	206104_at	KLF9	203542_s_at	MAX	209332_s_at	MLX	217910_x_at	MYT1	215822_x_at
HOXD3	206602_s_at	JUN	201464_x_at	KLF9	203543_s_at	MAX	210734_x_at	MLXIP	1554886_a_at	MYT1	1556269_at
HOXD3	217076_s_at	JUN	201465_s_at	KLF9	228474_s_at	MAX	214108_at	MLXIP	202519_at	MYT1L	1554633_a_at
HOXD4	1552337_s_at	JUN	201466_s_at	KLF9	230636_s_at	MAZ	207824_s_at	MLXIP	211789_s_at	MYT1L	210016_at
HOXD4	205522_at	JUN	213281_at	KNTC1	206316_s_at	MAZ	212064_x_at	MLXIP	225157_at	MYT1L	216672_s_at
HOXD9	205604_at	JUNB	201473_at	LBX1	208380_at	MBD1	1555611_s_at	MLXIPL	221163_s_at	MYT1L	241485_at
HOXD9	205605_at	JUND	203751_x_at	LHX2	206140_at	MBD1	203353_s_at	MNT	204206_at	MZF1	204138_s_at
HR	210086_at	JUND	203752_s_at	LHX2	211219_s_at	MBD1	208595_s_at	MNT	236749_at	MZF1	204139_x_at
HR	220163_s_at	JUND	214326_x_at	LHX3	221670_s_at	MBD1	226862_at	MSC	209928_s_at	MZF1	210336_x_at
HR	241355_at	JUND	229117_s_at	LHX5	208333_at	MBD1	241813_at	MSRB2	218773_s_at	MZF1	40569_at
HSF1	202344_at	KLF1	210504_at	LHX6	219884_at	MECP2	202616_s_at	MSRB2	219451_at	NANOG	220184_at
HSF1	213756_s_at	KLF10	202393_s_at	LHX6	224556_s_at	MECP2	202617_s_at	MSRB2	230575_at	NCOR1	200854_at
HSF2	209657_s_at	KLF11	1553137_s_at	LMO1	206718_at	MECP2	202618_s_at	MSX1	205932_s_at	NCOR1	200855_at
HSF2	211220_s_at	KLF11	218486_at	LMO4	209204_at	MECP2	241924_at	MSX1	228473_at	NCOR1	200856_x_at
HSF4	210977_s_at	KLF12	206965_at	LMO4	209205_s_at	MEF2A	208328_s_at	MSX2	205555_s_at	NCOR1	200857_s_at
HSF4	220080_at	KLF12	206966_s_at	LMO4	227155_at	MEF2A	212535_at	MSX2	210319_x_at	NCOR1	234313_at
IKZF1	1565817_at	KLF12	208467_at	LMO4	229537_at	MEF2A	214684_at	MSX2	205556_at	NCOR1	240314_at
IKZF1	1565818_s_at	KLF12	214276_at	LMO4	241922_at	MEF2A	242176_at	MTA1	202247_s_at	NEUROD1	1556057_s_at
IKZF1	205039_s_at	KLF12	227261_at	LMX1B	208487_at	MEF2B	205124_at	MTA1	211783_s_at	NEUROD1	206282_at
IKZF1	216901_s_at	KLF12	229881_at	LYL1	210044_s_at	MEF2C	207968_s_at	MTA2	203444_s_at	NEUROD2	1552953_a_at
IKZF1	220704_at	KLF12	238940_at	LZTFL1	218437_s_at	MEF2C	209199_s_at	MTF1	205322_s_at	NEUROD2	210271_at
IKZF1	227344_at	KLF12	239019_at	LZTFL1	222632_s_at	MEF2C	209200_at	MTF1	205323_s_at	NEUROG1	208497_x_at
IKZF1	227346_at	KLF13	1564463_at	LZTFL1	232418_at	MEF2D	203003_at	MTF1	227150_at	NEUROG3	207965_at
IKZF1	205038_at	KLF13	219878_s_at	LZTFL1	237553_at	MEF2D	203004_s_at	MXD1	206877_at	NFAT5	208003_s_at
IKZF4	208472_at	KLF13	225390_s_at	LZTR1	203412_at	MEF2D	225641_at	MXD1	212438_at	NFAT5	215092_s_at
IKZF4	226759_at	KLF13	230094_at	LZTS1	219042_at	MEIS1	1559477_s_at	MXD1	226275_at	NFAT5	224984_at
IKZF4	226761_at	KLF15	221302_at	LZTS1	221719_s_at	MEIS1	204069_at	MXD1	228846_at	NFATC1	208196_x_at
IKZF4	229752_at	KLF15	231015_at	LZTS1	221721_s_at	MEIS2	207480_s_at	MYB	204798_at	NFATC1	209664_x_at
IKZF5	220086_at	KLF2	219371_s_at	LZTS1	221722_x_at	MEIS3P1	214077_x_at	MYB	215152_at	NFATC1	210162_s_at
IKZF5	226680_at	KLF2	226646_at	LZTS1	222107_x_at	MEOX1	205619_s_at	MYBL1	213906_at	NFATC1	211105_s_at
ILF2	200052_s_at	KLF2	226645_at	LZTS1	47550_at	MEOX2	206201_s_at	MYBL1	231268_at	NFATC3	207416_s_at
INSM1	206502_s_at	KLF3	219657_s_at	LZTS1	1569159_at	MEOX2	206202_at	MYBL1	237014_at	NFATC3	210555_s_at
IRF1	202531_at	KLF3	222913_at	MAF	1566324_a_at	MGA	212945_s_at	MYBL2	201710_at	NFATC3	210556_at
IRF1	238725_at	KLF3	225133_at	MAF	206363_at	MGA	235409_at	MYC	202431_s_at	NFATC3	225137_at
IRF2	203275_at	KLF3	225140_at	MAF	209347_s_at	MGA	1564640_at	MYCL1	214058_at	NFATC3	225139_at
IRF3	202621_at	KLF4	220266_s_at	MAF	209348_s_at	MITF	1554874_at	MYCL1	215491_at	NFATC3	225141_at
IRF4	204562_at	KLF4	221841_s_at	MAF	1566323_at	MITF	207233_s_at	MYCN	209756_s_at	NFATC4	205897_at
IRF4	216986_s_at	KLF5	209211_at	MAFB	218559_s_at	MITF	226066_at	MYCN	209757_s_at	NFATC4	213345_at
IRF4	216987_at	KLF5	209212_s_at	MAFB	222670_s_at	MLLT10	1563321_s_at	MYCN	211377_x_at	NFATC4	236270_at
IRF5	205468_s_at	KLF6	1555832_s_at	MAFF	205193_at	MLLT10	205408_at	MYCN	234376_at	NFE2	209930_s_at
IRF5	205469_s_at	KLF6	208960_s_at	MAFF	36711_at	MLLT10	216503_s_at	MYCN	242026_at	NFE2L1	200758_s_at
IRF5	239412_at	KLF6	208961_s_at	MAFG	204970_s_at	MLLT10	216506_x_at	MYF6	206372_at	NFE2L1	200759_x_at
IRF6	1552477_a_at	KLF6	211610_at	MAFG	224466_s_at	MLLT10	225992_at	MYNN	218926_at	NFE2L1	214179_s_at
IRF6	1552478_a_at	KLF6	224606_at	MAFK	206750_at	MLLT10	230122_at	MYNN	224206_x_at	NFE2L2	1567013_at
IRF6	202597_at	KLF7	1555420_a_at	MAFK	226206_at	MLLT10	216480_x_at	MYNN	241730_at	NFE2L2	201146_at
IRF7	208436_s_at	KLF7	204334_at	MAML3	207946_at	MLLT10	216509_x_at	MYNN	1566109_at	NFE2L2	1567015_at
IRF7	220074_at	KLF7	238482_at	MAML3	228508_at	MLX	1570347_at	MYNN	237510_at	NFE2L3	204702_s_at
IRF8	204057_at	KLF7	238517_at	MAML3	242794_at	MLX	210752_s_at	MYOD1	206657_s_at	NFE2L3	236471_at
IRX4	220225_at	KLF7	240432_x_at	MAX	208403_x_at	MLX	213708_s_at	MYOG	207282_s_at	NFIB	209289_at

(Continued)

TF_name	Probes	TF_name	Probes	TF_name	Probes	TF_name	Probes	TF_name	Probes	TF_name	Probes
NFIB	209290_s_at	NKX2-2	206915_at	NR2E3	208385_at	ONECUT1	210745_at	PHTF1	210191_s_at	POU2F3	215355_at
NFIB	211466_at	NKX2-5	206578_at	NR2E3	208388_at	OVOL1	206604_at	PHTF1	215285_s_at	POU2F3	243383_at
NFIB	211467_s_at	NKX2-8	207451_at	NR2F1	209505_at	OVOL1	229396_at	PHTF1	235844_at	POU3F1	208345_s_at
NFIB	213029_at	NKX3-1	209706_at	NR2F1	209506_s_at	PAX1	1553492_a_at	PHTF2	1554780_a_at	POU3F1	210475_at
NFIB	213032_at	NKX3-1	211497_x_at	NR2F2	209119_x_at	PAX1	214401_at	PHTF2	1554822_at	POU3F2	207084_at
NFIB	213033_s_at	NKX3-1	211498_s_at	NR2F2	209120_at	PAX1	231445_at	PHTF2	209780_at	POU3F2	242455_at
NFIB	233304_at	NKX6-1	221366_at	NR2F2	209121_x_at	PAX2	206228_at	PHTF2	215286_s_at	POU3F3	208563_x_at
NFIB	233394_at	NOTCH2	202443_x_at	NR2F2	215073_s_at	PAX2	206229_x_at	PHTF2	217097_s_at	POU3F3	228780_at
NFIC	206929_s_at	NOTCH2	202445_s_at	NR2F2	229092_at	PAX3	207679_at	PITX1	1569561_at	POU3F3	237008_at
NFIC	213298_at	NOTCH2	210756_s_at	NR2F2	242396_at	PAX3	207680_x_at	PITX1	208502_s_at	POU3F4	207694_at
NFIC	226377_at	NOTCH2	212377_s_at	NR2F6	209261_s_at	PAX3	216059_at	PITX1	209587_at	POU4F1	206940_s_at
NFIC	226895_at	NOTCH2	227067_x_at	NR2F6	209262_s_at	PAX3	231666_at	PITX3	208277_at	POU4F1	211341_at
NFIL3	203574_at	NPAS2	205459_s_at	NR2F6	213354_s_at	PAX4	207867_at	PKNOX1	204195_s_at	POU4F1	239997_at
NFIX	209807_s_at	NPAS2	205460_at	NR3C1	201865_x_at	PAX4	211176_s_at	PKNOX1	216004_s_at	POU4F2	207725_at
NFIX	227400_at	NPAS2	213462_at	NR3C1	201866_s_at	PAX6	205646_s_at	PKNOX1	221883_at	POU6F1	205878_at
NFIX	228278_at	NPAS2	39548_at	NR3C1	211671_s_at	PAX6	235795_at	PKNOX1	54051_at	POU6F1	216330_s_at
NFIX	229834_at	NPAS2	39549_at	NR3C1	216321_s_at	PAX7	1556638_at	PKNOX1	204196_x_at	POU6F1	229809_at
NFKB1	209239_at	NPAS3	1554635_a_at	NR3C1	232431_at	PAX7	208060_at	PKNOX2	219046_s_at	POU6F1	216332_at
NFKB2	207535_s_at	NPAS3	220316_at	NR3C2	205259_at	PAX8	121_at	PKNOX2	222171_s_at	POU6F2	207450_s_at
NFKB2	209636_at	NPAS3	222172_at	NR4A1	202340_x_at	PAX8	207921_x_at	PKNOX2	63305_at	PPARA	1556064_at
NFKB2	211524_at	NPAS3	229281_at	NR4A1	210226_at	PAX8	207923_x_at	PKNOX2	222185_at	PPARA	1558631_at
NFRKB	206968_s_at	NPAS3	230412_at	NR4A1	211143_x_at	PAX8	207924_x_at	PLAG1	205372_at	PPARA	206870_at
NFRKB	213028_at	NPAS3	1569661_at	NR4A2	204621_s_at	PAX8	209552_at	PLAGL1	207002_s_at	PPARA	210771_at
NFRKB	237209_s_at	NPAS3	233293_at	NR4A2	204622_x_at	PAX8	213917_at	PLAGL1	207943_x_at	PPARA	223437_at
NFRKB	237210_at	NPAS3	233865_at	NR4A2	216248_s_at	PAX8	214528_s_at	PLAGL1	209318_x_at	PPARA	223438_s_at
NFX1	1553103_at	NPAS3	234014_at	NR4A2	235739_at	PAX8	221990_at	PLAGL2	202924_s_at	PPARA	226978_at
NFX1	1553348_a_at	NPAT	209798_at	NR4A3	207978_s_at	PAX9	207059_at	PLAGL2	202925_s_at	PPARA	237142_at
NFX1	202584_at	NPAT	211584_s_at	NR4A3	209959_at	PAX9	231145_at	PLAGL2	233617_at	PPARA	244689_at
NFX1	202585_s_at	NPAT	211585_at	NR4A3	216979_at	PBX1	205253_at	PML	206503_x_at	PPARA	1560981_a_at
NFX1	210268_at	NR0B1	206644_at	NR5A1	210333_at	PBX1	212148_at	PML	210362_x_at	PPARD	208044_s_at
NFYA	204107_at	NR0B1	206645_s_at	NR5A2	1560469_at	PBX1	212151_at	PML	211012_s_at	PPARD	210636_at
NFYA	204108_at	NR0B2	206410_at	NR5A2	208337_s_at	PBX2	202875_s_at	PML	211013_x_at	PPARD	37152_at
NFYA	204109_s_at	NR1D2	209750_at	NR5A2	208343_s_at	PBX2	202876_s_at	PML	211014_s_at	PPARG	208510_s_at
NFYA	215720_s_at	NR1D2	225768_at	NR5A2	210174_at	PBX2	211096_at	PML	211588_s_at	PRDM1	217192_s_at
NFYA	228433_at	NR1H2	218215_s_at	NR6A1	207742_s_at	PBX2	211097_s_at	PML	235508_at	PRDM1	228964_at
NFYA	228431_at	NR1H3	203920_at	NR6A1	210391_at	PBX3	204082_at	PML	239582_at	PRDM1	235668_at
NFYB	218127_at	NR1H4	1554375_a_at	NR6A1	210392_x_at	PCGF2	203792_x_at	PML	209640_at	PRDM16	220928_s_at
NFYB	218128_at	NR1H4	206340_at	NR6A1	211402_x_at	PCGF2	203793_x_at	PML	211589_at	PRDM16	232424_at
NFYB	218129_s_at	NR1H4	243800_at	NR6A1	227494_at	PCGF2	213551_x_at	POU2F1	206789_s_at	PRDM2	203056_s_at
NFYB	244704_at	NR1I2	207202_s_at	NR6A1	227496_at	PCGF2	214239_x_at	POU2F1	227254_at	PRDM2	203057_s_at
NFYC	1559218_s_at	NR1I2	207203_s_at	NR6A1	228894_at	PEG3	209242_at	POU2F1	229753_at	PRDM2	205277_at
NFYC	202215_s_at	NR1I3	1570188_at	NRF1	204651_at	PEG3	209243_s_at	POU2F1	244006_at	PRDM2	216433_s_at
NFYC	202216_x_at	NR1I3	207007_at	NRF1	204652_s_at	PFDN1	201507_at	POU2F2	211660_at	PRDM2	216445_at
NFYC	211251_x_at	NR2C1	204791_at	NRF1	211280_s_at	PGR	208305_at	POU2F2	211771_s_at	PREB	217861_s_at
NFYC	211797_s_at	NR2C1	210530_s_at	NRF1	243189_at	PGR	228554_at	POU2F2	227749_at	PROP1	211223_at
NFYC	238231_at	NR2C1	210531_at	NRF1	211279_at	PHF2	212726_at	POU2F2	228343_at	PRRX1	205991_s_at
NHLH1	214628_at	NR2C1	229956_at	NRL	206596_s_at	PHF2	207138_at	POU2F2	229750_at	PRRX1	226695_at
NHLH2	214497_s_at	NR2C2	206038_s_at	NRL	206597_at	PHOX2A	214609_at	POU2F2	235661_at	PRRX1	238852_at
NHLH2	215228_at	NR2C2	225477_s_at	OLIG2	213824_at	PHOX2B	207009_at	POU2F2	242866_x_at	PRRX2	219729_at
NKRF	205004_at	NR2E1	207443_at	OLIG2	213825_at	PHTF1	205702_at	POU2F3	207109_at	PTTG1	203554_x_at

(Continued)

TF_name	Probes	TF_name	Probes	TF_name	Probes	TF_name	Probes	TF_name	Probes	TF_name	Probes
PURA	204020_at	RFXANK	202758_s_at	RUNX2	236858_s_at	SLC2A4RG	227362_at	SOLH	204275_at	SP3	238035_at
PURA	204021_s_at	RFXAP	208492_at	RUNX2	236859_at	SLC30A9	202614_at	SOLH	230295_at	SP3	227537_s_at
PURA	213806_at	RFXAP	229431_at	RUNX2	221283_at	SLC30A9	229500_at	SOX1	208533_at	SP3	232529_at
PURA	229167_at	RLF	204243_at	RUNX3	204197_s_at	SMAD1	208015_at	SOX1	237472_at	SP4	206663_at
RARA	1565358_at	RNF4	212696_s_at	RUNX3	204198_s_at	SMAD1	210993_s_at	SOX1	230982_at	SP4	236265_at
RARA	203749_s_at	RORA	210426_x_at	RUNX3	234928_x_at	SMAD1	227798_at	SOX10	209842_at	SPDEF	213441_x_at
RARA	203750_s_at	RORA	210479_s_at	RXRA	202426_s_at	SMAD2	203075_at	SOX10	209843_s_at	SPDEF	214404_x_at
RARA	211605_s_at	RORA	226682_at	RXRA	202449_s_at	SMAD2	203076_s_at	SOX11	204913_s_at	SPDEF	220192_x_at
RARA	216300_x_at	RORA	235567_at	RXRB	209148_at	SMAD2	203077_s_at	SOX11	204914_s_at	SPDEF	213442_x_at
RARB	205080_at	RORA	236266_at	RXRB	215098_at	SMAD2	226563_at	SOX11	204915_s_at	SPDEF	214403_x_at
RARB	208412_s_at	RORA	240951_at	RXRB	215099_s_at	SMAD2	235598_at	SOX12	204432_at	SPI1	205312_at
RARB	208413_at	RORA	1562682_at	RXRG	205954_at	SMAD2	239271_at	SOX12	228358_at	SPIB	205861_at
RARB	208530_s_at	RORA	239550_at	SALL1	206893_at	SMAD3	205396_at	SOX13	1569111_at	SPIB	232739_at
RARB	217020_at	RORA	240163_at	SALL1	229273_at	SMAD3	205397_x_at	SOX13	209736_at	SREBF1	1558875_at
RARG	204188_s_at	RORA	241760_x_at	SALL2	213283_s_at	SMAD3	205398_s_at	SOX13	38918_at	SREBF1	202308_at
RARG	204189_at	RORB	206443_at	SATB1	203408_s_at	SMAD3	218284_at	SOX15	206122_at	SREBF2	201247_at
RARG	217178_at	RORB	231040_at	SATB1	241365_at	SMAD4	202526_at	SOX15	217040_x_at	SREBF2	201248_s_at
RAX	208242_at	RORB	242385_at	SATB1	244267_at	SMAD4	202527_s_at	SOX17	219993_at	SREBF2	242748_at
RB1	203132_at	RORC	206419_at	SATB2	213435_at	SMAD4	235725_at	SOX17	230943_at	SRF	202400_s_at
RB1	211540_s_at	RORC	228806_at	SATB2	215591_at	SMAD4	1565702_at	SOX18	219568_x_at	SRF	202401_s_at
RBL2	212331_at	RREB1	203704_s_at	SCAND1	218206_x_at	SMAD4	1565703_at	SOX2	213721_at	ST18	1557583_at
RBL2	212332_at	RREB1	217411_s_at	SCAND1	231059_x_at	SMAD5	205187_at	SOX2	213722_at	ST18	1570307_s_at
REL	206036_s_at	RREB1	242297_at	SCAND1	232652_x_at	SMAD5	205188_s_at	SOX2	228038_at	ST18	206135_at
REL	228812_at	RREB1	215032_at	SCML1	218793_s_at	SMAD5	225219_at	SOX2	214178_s_at	ST18	1570306_at
REL	235242_at	RREB1	215620_at	SCML1	222747_s_at	SMAD5	225223_at	SOX21	208468_at	STAT1	200887_s_at
REL	239486_at	RREB1	216648_s_at	SCML2	206147_x_at	SMAD5	235451_at	SOX3	1553802_a_at	STAT1	209969_s_at
REL	206035_at	RREB1	216649_at	SHOX	207570_at	SMAD6	207069_s_at	SOX3	214633_at	STAT1	AFFX-HUMISGF3A/M97935_3_at
RELA	201783_s_at	RUNX1	1563591_at	SHOX2	208443_x_at	SMAD6	209886_s_at	SOX4	201416_at	STAT1	AFFX-HUMISGF3A/M97935_5_at
RELA	209878_s_at	RUNX1	1570350_at	SHOX2	210134_x_at	SMAD6	209887_at	SOX4	201417_at	STAT1	AFFX-HUMISGF3A/M97935_MA_at
RELB	205205_at	RUNX1	208129_x_at	SHOX2	210135_s_at	SMAD6	213565_s_at	SOX4	201418_s_at	STAT1	AFFX-HUMISGF3A/M97935_MB_at
RERE	1557349_at	RUNX1	209359_x_at	SIM2	1557900_at	SMAD7	204790_at	SOX4	213668_s_at	STAT2	205170_at
RERE	200938_s_at	RUNX1	209360_s_at	SIM2	206558_at	SMAD9	206320_s_at	SOX4	1567906_at	STAT2	217199_s_at
RERE	200939_s_at	RUNX1	210365_at	SIM2	208157_at	SMAD9	227719_at	SOX4	213665_at	STAT2	225636_at
RERE	200940_s_at	RUNX1	210805_x_at	SIM2	237923_at	SMARCA4	1569073_x_at	SOX5	1569638_at	STAT3	208991_at
RERE	221643_s_at	RUNX1	211180_x_at	SIM2	241726_at	SMARCA4	208793_x_at	SOX5	207336_at	STAT3	208992_s_at
REST	204535_s_at	RUNX1	211181_x_at	SIX1	205817_at	SMARCA4	208794_s_at	SOX5	238009_at	STAT3	225289_at
REST	212920_at	RUNX1	211182_x_at	SIX1	228347_at	SMARCA4	212520_s_at	SOX5	238285_at	STAT3	243213_at
REXO4	218767_at	RUNX1	211620_x_at	SIX1	230911_at	SMARCA4	213720_s_at	SOX9	202935_s_at	STAT4	206118_at
RFX1	206321_at	RUNX1	217263_x_at	SIX2	206510_at	SMARCA4	214728_x_at	SOX9	202936_s_at	STAT5A	203010_at
RFX1	222012_at	RUNX1	237697_at	SIX2	206511_s_at	SMARCA4	215714_s_at	SP1	1553685_s_at	STAT5B	1555086_at
RFX1	226786_at	RUNX1T1	1564642_at	SIX3	206634_at	SMARCA4	213719_s_at	SP1	214732_at	STAT5B	1555088_x_at
RFX2	208031_s_at	RUNX1T1	205528_s_at	SIX3	242054_s_at	SMARCA4	214360_at	SP1	224754_at	STAT5B	205026_at
RFX2	226872_at	RUNX1T1	205529_s_at	SIX5	217661_x_at	SNAI1	219480_at	SP1	224760_at	STAT5B	212549_at
RFX2	242966_x_at	RUNX1T1	216831_s_at	SIX5	229009_at	SNAI2	213139_at	SP140	1565588_at	STAT5B	212550_at
RFX3	207234_at	RUNX1T1	216832_at	SIX6	207250_at	SNAPC2	204104_at	SP140	207777_s_at	STAT6	201331_s_at
RFX3	230403_at	RUNX1T1	228827_at	SLC26A3	206143_at	SNAPC4	205658_s_at	SP2	204367_at	STAT6	201332_s_at
RFX3	238810_at	RUNX1T1	242845_at	SLC26A3	215657_at	SNAPC4	215926_x_at	SP2	211736_at	SUPT4H1	201483_s_at
RFX3	243868_at	RUNX2	216994_s_at	SLC2A4RG	1555500_s_at	SNAPC5	1554093_a_at	SP2	237795_s_at	SUPT4H1	201484_at
RFX5	202963_at	RUNX2	221282_x_at	SLC2A4RG	218494_s_at	SNAPC5	213203_at	SP3	213168_at	SUPT6H	1554311_a_at
RFX5	202964_s_at	RUNX2	232231_at	SLC2A4RG	222650_s_at	SNAPC5	214592_s_at	SP3	229217_at	SUPT6H	208420_x_at

(Continued)

TF_name	Probes	TF_name	Probes	TF_name	Probes	TF_name	Probes	TF_name	Probes	TF_name	Probes
SUPT6H	208830_s_at	TCF25	221495_s_at	TFAP2A	204653_at	TRERF1	233324_at	VSX1	221124_s_at	ZBTB7B	235145_at
SUPT6H	208831_x_at	TCF25	214704_at	TFAP2A	204654_s_at	TRERF1	238520_at	VSX1	222972_at	ZFHX4	1559270_at
T	206524_at	TCF3	209151_x_at	TFAP2A	210669_at	TRIM22	213293_s_at	VSX1	224074_at	ZFHX4	219779_at
TAF1B	214690_at	TCF3	209152_s_at	TFAP2B	1553394_a_at	TRIM25	206911_at	VSX1	224075_s_at	ZFHX4	241700_at
TAF1B	216941_s_at	TCF3	209153_s_at	TFAP2B	214451_at	TRIM25	224806_at	WT1	206067_s_at	ZFP36L1	211962_s_at
TAF5L	1554415_at	TCF3	210776_x_at	TFAP2B	215686_x_at	TRIM28	200990_at	WT1	216953_s_at	ZFP36L1	211965_at
TAF5L	213654_at	TCF3	213730_x_at	TFAP2C	205286_at	TRIM29	202504_at	XBP1	200670_at	ZFP36L1	213277_at
TAL1	1561651_s_at	TCF3	213811_x_at	TFAP2C	205287_s_at	TRIM29	211001_at	XBP1	242021_at	ZFP36L1	229054_at
TAL1	206283_s_at	TCF3	215260_s_at	TFAP4	205688_at	TRIM29	211002_s_at	YBX1	208627_s_at	ZFP36L1	213284_at
TAL1	216925_s_at	TCF3	216645_at	TFCP2	207627_s_at	TRPS1	218502_s_at	YBX1	208628_s_at	ZFP36L2	201367_s_at
TAL1	216928_at	TCF3	202648_at	TFCP2	209338_at	TRPS1	222651_s_at	YBX1	216940_x_at	ZFP36L2	201368_at
TARDBP	200020_at	TCF3	213731_s_at	TFCP2	227637_at	TRPS1	224218_s_at	YEATS4	218911_at	ZFP36L2	201369_s_at
TARDBP	221264_s_at	TCF3	213732_at	TFCP2L1	219735_s_at	TRPS1	234351_x_at	YWHAE	210317_s_at	ZFP36L2	227681_at
TBR1	220025_at	TCF3	213809_x_at	TFCP2L1	227642_at	TSC22D1	215111_s_at	YWHAE	210996_s_at	ZFP37	207068_at
TBX1	207662_at	TCF3	216647_at	TFCP2L1	229341_at	TSC22D1	235315_at	YWHAZ	1556496_a_at	ZFX	207247_s_at
TBX1	211273_s_at	TCF3	228052_x_at	TFDP1	204147_s_at	TSC22D1	239123_at	YWHAZ	200638_s_at	ZFX	207920_x_at
TBX1	211274_at	TCF4	203753_at	TFDP1	212330_at	TSC22D1	243133_at	YWHAZ	200639_s_at	ZFX	214678_x_at
TBX1	236926_at	TCF4	212382_at	TFDP1	242939_at	TSC22D2	204094_s_at	YWHAZ	200640_at	ZFX	217176_s_at
TBX1	242941_x_at	TCF4	212385_at	TFDP2	203588_s_at	TSC22D2	210953_at	YWHAZ	200641_s_at	ZFX	229022_at
TBX10	207689_at	TCF4	212386_at	TFDP2	203589_s_at	TSC22D2	210954_s_at	YWHAZ	214848_at	ZFY	207246_at
TBX10	222377_at	TCF4	212387_at	TFDP2	226157_at	TSC22D2	238932_at	YY1	200047_s_at	ZFY	207247_s_at
TBX19	206838_at	TCF4	213891_s_at	TFDP2	244043_at	TSC22D2	240557_at	YY1	201901_s_at	ZFY	230760_at
TBX2	205993_s_at	TCF4	222146_s_at	TFDP3	207385_at	TSC22D2	215547_at	YY1	201902_s_at	ZHX2	203556_at
TBX2	213417_at	TCF4	228837_at	TFE3	1565347_s_at	TSC22D3	207001_x_at	YY1	224711_at	ZHX2	236169_at
TBX2	40560_at	TCF7	205254_x_at	TFE3	206649_s_at	TSC22D3	208763_s_at	YY1	224718_at	ZHX2	1557706_at
TBX2	221977_at	TCF7	205255_x_at	TFE3	212457_at	TSC22D3	235364_at	YY1	213494_s_at	ZHX3	212545_s_at
TBX21	220684_at	TCF7L1	221016_s_at	TFE3	1567704_at	TSC22D4	1554501_at	YY2	216531_at	ZHX3	217367_s_at
TBX3	219682_s_at	TCF7L2	212759_s_at	TFEB	221866_at	TSC22D4	208104_s_at	ZBTB16	205883_at	ZIC1	206373_at
TBX3	222917_s_at	TCF7L2	212761_at	TFEB	50221_at	TULP4	218184_at	ZBTB17	203601_s_at	ZIC1	236896_at
TBX3	225544_at	TCF7L2	212762_s_at	TFEC	206715_at	TULP4	224170_s_at	ZBTB17	203602_s_at	ZIC1	241440_at
TBX3	229576_s_at	TCF7L2	216035_x_at	TFEC	232383_at	TULP4	239742_at	ZBTB22	213081_at	ZIC1	234716_at
TBX3	228344_s_at	TCF7L2	216037_x_at	TFEC	236995_x_at	TWIST1	213943_at	ZBTB25	214482_at	ZIM2	220653_at
TBX3	229565_x_at	TCF7L2	216511_s_at	TGIF2	216262_s_at	UBN1	207253_s_at	ZBTB38	1558733_at	ZKSCAN1	1557953_at
TBX3	243234_at	TCF7L2	236094_at	TGIF2	218724_s_at	UBN1	209088_s_at	ZBTB38	219221_at	ZKSCAN1	214670_at
TBX4	220634_at	TCFL5	204849_at	THRA	1316_at	UBP1	218082_s_at	ZBTB38	225512_at	ZKSCAN1	214900_at
TBX5	207155_at	TCFL5	235694_at	THRA	204100_at	USF2	202152_x_at	ZBTB38	230754_at	ZKSCAN1	225221_at
TBX5	211886_s_at	TEAD1	1553322_s_at	THRA	214883_at	USF2	214879_x_at	ZBTB38	236557_at	ZMYM2	202778_s_at
TBX5	240715_at	TEAD1	214600_at	THRA	35846_at	USF2	215737_x_at	ZBTB43	204180_s_at	ZMYM2	210281_s_at
TBX5	1563018_at	TEAD1	224955_at	THRB	207044_at	VAV1	206219_s_at	ZBTB43	204181_s_at	ZMYM2	210282_at
TBX6	207684_at	TEAD3	209454_s_at	THRB	228716_at	VAX2	220443_s_at	ZBTB43	204182_s_at	ZMYM2	226512_at
TBX6	215122_at	TEAD4	204281_at	THRB	229657_at	VDR	204253_s_at	ZBTB43	227991_x_at	ZMYM2	240642_at
TCEAL1	204045_at	TEAD4	41037_at	TLX1	207179_at	VDR	204254_s_at	ZBTB43	231393_x_at	ZMYM2	242597_at
TCF12	208986_at	TEF	210167_s_at	TLX2	207410_s_at	VDR	204255_s_at	ZBTB6	206098_at	ZMYM3	1554171_at
TCF12	238041_at	TEF	225840_at	TLX2	211049_at	VDR	213692_s_at	ZBTB7A	213299_at	ZMYM3	1554172_a_at
TCF12	215611_at	TEF	215673_at	TP53	201746_at	VENTX	207376_at	ZBTB7A	213303_x_at	ZMYM3	207559_s_at
TCF15	207306_at	TFAM	203176_s_at	TP53	211300_s_at	VEZF1	202171_at	ZBTB7A	219186_at	ZMYM4	202049_s_at
TCF21	1559277_at	TFAM	203177_x_at	TP73	1554379_a_at	VEZF1	202172_at	ZBTB7A	226554_at	ZMYM4	202050_s_at
TCF21	204931_at	TFAM	208541_x_at	TP73	220804_s_at	VEZF1	202173_s_at	ZBTB7A	222082_at	ZMYM4	202051_s_at
TCF21	229529_at	TFAM	238443_at	TP73	232546_at	VPS72	202261_at	ZBTB7A	230709_x_at	ZNF10	216350_s_at
TCF25	213311_s_at	TFAM	238836_at	TRERF1	229016_s_at	VPS72	223762_at	ZBTB7B	205853_at	ZNF10	229848_at

(Continued)

TF_name	Probes	TF_name	Probes	TF_name	Probes	TF_name	Probes	TF_name	Probes	TF_name	Probes
ZNF10	235366_at	ZNF174	210291_s_at	ZNF227	217403_s_at	ZNF281	222619_at	ZNF384	212369_at	ZNF467	213559_s_at
ZNF117	207117_at	ZNF175	205497_at	ZNF227	227689_at	ZNF281	228785_at	ZNF384	220616_at	ZNF468	214751_at
ZNF117	207605_x_at	ZNF175	243128_at	ZNF230	1557322_at	ZNF282	212892_at	ZNF394	214714_at	ZNF471	211923_s_at
ZNF117	235408_x_at	ZNF177	207417_s_at	ZNF230	1570135_at	ZNF287	220055_at	ZNF394	243024_at	ZNF471	230578_at
ZNF117	235564_at	ZNF180	219495_s_at	ZNF230	205791_x_at	ZNF287	216453_at	ZNF395	218149_s_at	ZNF471	232117_at
ZNF12	1554021_a_at	ZNF180	230065_at	ZNF232	219123_at	ZNF287	216710_x_at	ZNF395	221123_x_at	ZNF473	213124_at
ZNF12	1559881_s_at	ZNF184	213452_at	ZNF235	210595_at	ZNF292	1562991_at	ZNF395	222536_s_at	ZNF473	213130_at
ZNF12	219571_s_at	ZNF185	203585_at	ZNF235	220350_at	ZNF292	212366_at	ZNF395	223216_x_at	ZNF480	222283_at
ZNF12	226015_at	ZNF189	207513_s_at	ZNF236	219171_s_at	ZNF292	212368_at	ZNF395	232693_s_at	ZNF484	214922_at
ZNF124	206928_at	ZNF195	204234_s_at	ZNF236	47571_at	ZNF3	212684_at	ZNF395	232694_at	ZNF493	1558486_at
ZNF124	234394_at	ZNF197	205855_at	ZNF239	206261_at	ZNF3	219604_s_at	ZNF407	220835_s_at	ZNF493	211064_at
ZNF131	1557384_at	ZNF197	233070_at	ZNF24	1554045_at	ZNF3	219605_at	ZNF407	220836_at	ZNF493	235604_x_at
ZNF131	214741_at	ZNF20	213916_at	ZNF24	203247_s_at	ZNF3	232497_at	ZNF407	227768_at	ZNF493	240155_x_at
ZNF131	221842_s_at	ZNF200	207338_s_at	ZNF24	203248_at	ZNF302	218490_s_at	ZNF407	234844_at	ZNF500	213639_s_at
ZNF131	225916_at	ZNF200	214706_at	ZNF24	212534_at	ZNF302	228392_at	ZNF408	219224_x_at	ZNF500	213641_at
ZNF132	207402_at	ZNF202	204327_s_at	ZNF24	242210_at	ZNF302	228393_s_at	ZNF410	202010_s_at	ZNF500	41113_at
ZNF133	216960_s_at	ZNF202	204329_s_at	ZNF248	1569557_at	ZNF304	207753_at	ZNF410	209944_at	ZNF506	1568720_at
ZNF133	37254_at	ZNF205	206416_at	ZNF248	213269_at	ZNF318	203520_s_at	ZNF415	205514_at	ZNF506	221626_at
ZNF134	206182_at	ZNF207	1556035_s_at	ZNF250	213858_at	ZNF318	203521_s_at	ZNF423	214761_at	ZNF506	238493_at
ZNF134	227729_at	ZNF207	200828_s_at	ZNF250	223198_x_at	ZNF318	231388_at	ZNF426	205964_at	ZNF507	206225_at
ZNF135	206142_at	ZNF207	200829_x_at	ZNF250	223819_x_at	ZNF32	209538_at	ZNF43	206695_x_at	ZNF507	226327_at
ZNF135	1567410_at	ZNF207	228157_at	ZNF250	241738_at	ZNF324	205182_s_at	ZNF43	222136_x_at	ZNF507	235618_at
ZNF136	206240_s_at	ZNF207	229765_at	ZNF253	206900_x_at	ZNF329	219765_at	ZNF430	206829_x_at	ZNF510	206053_at
ZNF14	219854_at	ZNF207	231848_x_at	ZNF253	242919_at	ZNF330	209814_at	ZNF430	238614_x_at	ZNF510	228917_at
ZNF140	204523_at	ZNF207	239937_at	ZNF259	200054_at	ZNF330	213760_s_at	ZNF432	219848_s_at	ZNF516	203604_at
ZNF141	206931_at	ZNF207	244153_at	ZNF259	217185_s_at	ZNF331	219228_at	ZNF44	206810_at	ZNF528	215019_x_at
ZNF141	235580_at	ZNF207	238772_at	ZNF26	1555325_s_at	ZNF331	227613_at	ZNF44	215359_x_at	ZNF529	215307_at
ZNF142	204474_at	ZNF211	205437_at	ZNF26	219595_at	ZNF334	220022_at	ZNF44	223968_at	ZNF529	231940_at
ZNF142	37586_at	ZNF212	203985_at	ZNF263	203707_at	ZNF334	238566_at	ZNF44	233712_at	ZNF529	237453_at
ZNF143	221873_at	ZNF215	1555510_at	ZNF263	232089_at	ZNF335	221890_at	ZNF44	228718_at	ZNF532	220617_s_at
ZNF146	1554433_a_at	ZNF215	220214_at	ZNF263	243547_at	ZNF335	222059_at	ZNF440	215892_at	ZNF532	225021_at
ZNF146	200050_at	ZNF217	203739_at	ZNF264	1558698_at	ZNF335	78330_at	ZNF440	241731_x_at	ZNF536	206403_at
ZNF148	203318_s_at	ZNF219	219314_s_at	ZNF264	205917_at	ZNF337	214760_at	ZNF440	216291_at	ZNF536	239340_at
ZNF148	203319_s_at	ZNF219	222864_s_at	ZNF264	230063_at	ZNF337	37860_at	ZNF443	205928_at	ZNF536	242715_at
ZNF148	228545_at	ZNF219	1558594_at	ZNF266	214686_at	ZNF33B	215022_x_at	ZNF444	218707_at	ZNF544	218735_s_at
ZNF148	230821_at	ZNF22	218005_at	ZNF267	219540_at	ZNF33B	1558586_at	ZNF444	50376_at	ZNF544	244466_at
ZNF148	235166_at	ZNF22	218006_s_at	ZNF268	209989_at	ZNF33B	1562743_at	ZNF446	1554160_a_at	ZNF549	215315_at
ZNF148	238711_s_at	ZNF221	220847_x_at	ZNF268	238030_at	ZNF34	219801_at	ZNF446	205182_s_at	ZNF550	215124_at
ZNF148	239024_at	ZNF221	233848_x_at	ZNF271	211009_s_at	ZNF343	207296_at	ZNF446	219900_s_at	ZNF550	228099_at
ZNF154	217242_at	ZNF222	206175_x_at	ZNF271	236231_at	ZNF343	227986_at	ZNF45	207304_at	ZNF551	211721_s_at
ZNF154	216677_at	ZNF223	207128_s_at	ZNF271	238007_at	ZNF345	207236_at	ZNF45	222028_at	ZNF551	243194_at
ZNF155	221201_s_at	ZNF224	216983_s_at	ZNF273	215239_x_at	ZNF345	240199_x_at	ZNF451	1556060_a_at	ZNF551	243195_s_at
ZNF16	219548_at	ZNF224	220019_s_at	ZNF273	243661_at	ZNF35	206096_at	ZNF451	212557_at	ZNF551	242992_at
ZNF160	1567032_s_at	ZNF224	244462_at	ZNF274	204937_s_at	ZNF350	219266_at	ZNF451	215012_at	ZNF552	219741_x_at
ZNF160	214715_x_at	ZNF224	232427_at	ZNF274	232436_at	ZNF350	233169_at	ZNF451	231885_at	ZNF552	232328_at
ZNF160	224014_at	ZNF225	207125_at	ZNF277	1555192_at	ZNF354A	205427_at	ZNF451	232029_at	ZNF556	220709_at
ZNF160	239954_at	ZNF226	219603_s_at	ZNF277	1555193_a_at	ZNF358	219379_x_at	ZNF460	216279_at	ZNF557	220444_at
ZNF160	1567031_at	ZNF226	224004_at	ZNF277	215887_at	ZNF358	226260_x_at	ZNF460	233175_at	ZNF557	239259_at
ZNF174	205252_at	ZNF226	231717_s_at	ZNF277	218645_at	ZNF365	1570121_at	ZNF460	216273_at	ZNF562	219163_at
ZNF174	210290_at	ZNF226	233461_x_at	ZNF281	218401_s_at	ZNF365	206448_at	ZNF467	214746_s_at	ZNF562	241089_at

(Continued)

TF_name	Probes	TF_name	Probes	TF_name	Probes	TF_name	Probes	TF_name	Probes	TF_name	Probes
ZNF573	217627_at	ZNF606	219635_at	ZNF652	235577_at	ZNF688	235950_at	ZNF710	235090_at	ZNF85	206572_x_at
ZNF573	1556513_at	ZNF606	229707_at	ZNF652	243495_s_at	ZNF688	235951_s_at	ZNF711	207781_s_at	ZNF91	1569277_at
ZNF574	218762_at	ZNF609	212617_at	ZNF654	219239_s_at	ZNF688	228631_s_at	ZNF711	228988_at	ZNF91	206059_at
ZNF576	219088_s_at	ZNF609	212618_at	ZNF654	222851_at	ZNF692	220661_s_at	ZNF74	205881_at	ZNF91	236128_at
ZNF576	219089_s_at	ZNF609	212620_at	ZNF654	241348_at	ZNF692	227011_at	ZNF74	235997_at	ZNF91	244259_s_at
ZNF580	220748_s_at	ZNF611	1559059_s_at	ZNF665	220760_x_at	ZNF695	208273_at	ZNF79	214138_at	ZNF93	1569240_at
ZNF586	219711_at	ZNF611	208137_x_at	ZNF667	207120_at	ZNF696	220967_s_at	ZNF79	216486_x_at	ZNF93	1569241_a_at
ZNF586	1554665_at	ZNF611	238570_at	ZNF667	236635_at	ZNF696	232162_at	ZNF79	216482_x_at	ZNF93	208119_s_at
ZNF587	1558251_a_at	ZNF614	210599_at	ZNF668	219047_s_at	ZNF7	1559980_at	ZNF8	214901_at	ZNF93	215758_x_at
ZNF587	1558253_x_at	ZNF614	220721_at	ZNF668	222813_at	ZNF7	205089_at	ZNF81	1561038_at		
ZNF587	219981_x_at	ZNF614	227045_at	ZNF669	220215_at	ZNF7	234914_at	ZNF81	1561039_a_at		
ZNF587	231820_x_at	ZNF623	206188_at	ZNF671	219849_at	ZNF7	234920_at	ZNF81	215901_at		
ZNF589	1569108_a_at	ZNF629	213196_at	ZNF672	218068_s_at	ZNF701	220242_x_at	ZNF81	234111_at		
ZNF589	210061_at	ZNF638	1554248_at	ZNF672	222509_s_at	ZNF701	236589_at	ZNF81	240079_at		
ZNF589	210062_s_at	ZNF638	1554249_a_at	ZNF672	227011_at	ZNF706	218059_at	ZNF81	242083_at		
ZNF589	219968_at	ZNF638	211257_x_at	ZNF675	217547_x_at	ZNF706	227132_at	ZNF83	221645_s_at		
ZNF592	204473_s_at	ZNF638	213775_x_at	ZNF682	216468_s_at	ZNF710	213542_at	ZNF83	236429_at		
ZNF592	213389_at	ZNF646	204876_at	ZNF682	242915_at	ZNF710	239335_at	ZNF84	204453_at		
ZNF592	227507_at	ZNF652	205594_at	ZNF688	213527_s_at	ZNF710	239700_at	ZNF84	228630_at		
ZNF593	204175_at	ZNF652	225266_at	ZNF688	213529_at	ZNF710	39891_at	ZNF85	1554445_at		

Table S3. Original sequences in Homo sapiens for Homologous sequence analysis

400 bps long sequence
>Homo_sapiens chromosome 9, 87284301-87284700, hg19<br>GAGACAGCCTCTACCGCGATTGTAGAAGAGACTGTGGTGTGAATTAGGGA<br>CCGGGAGGCGTCGAACGGAGGAACGGTTCATCTTAGAGGTACCTGGATGT<br>AAATGCACACACACACACACAGACACACACACGCACGCGCGTGCATGTCTAC<br>ACGGCCAGGATGTGTGCGTGTGTGCGCGCGTGTGTGAACTCCCACATGCT<br>GCTGCTGTCTGCTTCTGGCCAGTGGCACCGATGCCTCCCTCCTCCCTGCT<br>CGCCCCCAGATTCCCCTCCCCTCCCTGGTGCTTTTGTCTGGAGGGTGTTA<br>TGGGTTTGTGTGTGTATGAGCGTGTGTGTGTTTTTGGATTTCAGACTAAT<br>TTTCTGGAGTTTCTGCCCCTGCTCTGCGTCAGCCCTCACGTCACTTCGCC
118 bps long sequence
>Homo_sapiens chromosome 9, 87284582-87284699, hg19<br>TTTTGTCTGGAGGGTGTTATGGGTTTGTGTGTGTATGAGCGTGTGTGTGTT<br>TTTGGATTTCAGACTAATTTTCTGGAGTTTCTGCCCCTGCTCTGCGTCA<br>GCCCTCACGTCACTTCGC

Table S4 Top 100 DEGs between differentiated and un-differentiated SH-SY5Y-A cells

Probes	Feature P	FDR	Fold change	Probes	Feature P	FDR	Fold change
206424_at	2.00E-04	0.001344	5498.945	203505_at	2.00E-04	0.001344	16.88618
219825_at	2.00E-04	0.001344	2478.029	207035_at	2.00E-04	0.001344	16.73473
217561_at	2.00E-04	0.001344	260.5504	220807_at	2.00E-04	0.001344	16.66332
210258_at	2.00E-04	0.001344	246.308	206253_at	2.00E-04	0.001344	16.65793
235892_at	2.00E-04	0.001344	172.6947	204940_at	2.00E-04	0.001344	16.43247
234721_s_at	2.00E-04	0.001344	141.7612	205377_s_at	2.00E-04	0.001344	16.34786
217495_x_at	2.00E-04	0.001344	116.6897	228368_at	2.00E-04	0.001344	16.33982
202481_at	2.00E-04	0.001344	81.67856	229581_at	2.00E-04	0.001344	16.31556
233691_at	2.00E-04	0.001344	61.96367	220889_s_at	2.00E-04	0.001344	16.20698
238032_at	2.00E-04	0.001344	59.94732	240811_at	2.00E-04	0.001344	15.79937
229463_at	2.00E-04	0.001344	56.41827	229080_at	2.00E-04	0.001344	15.66408
228973_at	2.00E-04	0.001344	49.26989	204534_at	2.00E-04	0.001344	15.231
207531_at	2.00E-04	0.001344	48.7228	1568366_at	2.00E-04	0.001344	15.00619
205378_s_at	2.00E-04	0.001344	48.04883	214680_at	2.00E-04	0.001344	14.62637
1568752_s_at	2.00E-04	0.001344	45.73552	1562169_at	2.00E-04	0.001344	14.51387
236095_at	2.00E-04	0.001344	45.0862	228994_at	2.00E-04	0.001344	14.23171
1555564_a_at	2.00E-04	0.001344	41.5617	1562823_at	2.00E-04	0.001344	14.03163
221042_s_at	2.00E-04	0.001344	40.15656	205736_at	2.00E-04	0.001344	13.99536
210728_s_at	2.00E-04	0.001344	36.42928	208530_s_at	2.00E-04	0.001344	13.83247
1566948_at	2.00E-04	0.001344	36.0747	235700_at	2.00E-04	0.001344	13.71993
229290_at	2.00E-04	0.001344	35.55319	226311_at	2.00E-04	0.001344	13.44771
205890_s_at	2.00E-04	0.001344	34.90444	213839_at	2.00E-04	0.001344	13.23282
232390_at	2.00E-04	0.001344	32.14494	228375_at	2.00E-04	0.001344	13.22553
204939_s_at	2.00E-04	0.001344	31.53481	215149_at	2.00E-04	0.001344	13.17588
207241_at	2.00E-04	0.001344	31.17334	219073_s_at	2.00E-04	0.001344	12.772
206349_at	2.00E-04	0.001344	30.61114	221796_at	2.00E-04	0.001344	12.53958
1552721_a_at	2.00E-04	0.001344	30.27707	205080_at	2.00E-04	0.001344	12.49185
237056_at	2.00E-04	0.001344	27.28042	229641_at	2.00E-04	0.001344	11.90956
209392_at	2.00E-04	0.001344	26.43595	203854_at	2.00E-04	0.001344	11.90061
208510_s_at	2.00E-04	0.001344	24.63285	240509_s_at	2.00E-04	0.001344	11.7907
1556749_at	2.00E-04	0.001344	23.95865	230956_at	2.00E-04	0.001344	11.63011
211003_x_at	2.00E-04	0.001344	23.19597	226096_at	2.00E-04	0.001344	11.54168
210839_s_at	2.00E-04	0.001344	22.78349	237277_at	2.00E-04	0.001344	11.53799
202575_at	2.00E-04	0.001344	21.48805	206417_at	2.00E-04	0.001344	11.50323
215692_s_at	2.00E-04	0.001344	21.39254	215020_at	2.00E-04	0.001344	11.49844
205413_at	2.00E-04	0.001344	21.34419	239272_at	2.00E-04	0.001344	11.45193
219511_s_at	2.00E-04	0.001344	20.91752	231257_at	2.00E-04	0.001344	11.04554
214636_at	2.00E-04	0.001344	20.63945	204938_s_at	2.00E-04	0.001344	10.97231
244169_x_at	2.00E-04	0.001344	19.92679	237615_at	2.00E-04	0.001344	10.97103
206638_at	2.00E-04	0.001344	19.26709	242871_at	2.00E-04	0.001344	10.87726
217452_s_at	2.00E-04	0.001344	19.14135	209040_s_at	2.00E-04	0.001344	10.79754
238934_at	2.00E-04	0.001344	18.66337	1569453_a_at	2.00E-04	0.001344	10.64254
244887_at	2.00E-04	0.001344	18.5325	224278_at	2.00E-04	0.001344	10.55874
206828_at	2.00E-04	0.001344	18.41297	221795_at	2.00E-04	0.001344	10.48981
215571_at	2.00E-04	0.001344	18.00543	210727_at	2.00E-04	0.001344	10.37492
208552_at	2.00E-04	0.001344	17.79972	227429_at	2.00E-04	0.001344	10.3618
220333_at	2.00E-04	0.001344	17.60008	209793_at	2.00E-04	0.001344	10.15671
205669_at	2.00E-04	0.001344	17.26579	201042_at	2.00E-04	0.001344	10.03826
221755_at	2.00E-04	0.001344	17.16798	1563568_at	2.00E-04	0.001344	9.999214
223600_s_at	2.00E-04	0.001344	17.01475	204684_at	2.00E-04	0.001344	9.987734

Table S5 Top 100 DEGs between differentiated and un-differentiated SH-SY5Y-E cells

Probe	Feature P	FDR	Fold change	Probe	Feature P	FDR	Fold change
206424_at	2.00E-04	0.001822	72.65859047	237808_at	2.00E-04	0.001822	12.228676
219825_at	2.00E-04	0.001822	45.94439653	229308_at	2.00E-04	0.001822	12.16639
214680_at	2.00E-04	0.001822	44.82897543	235066_at	2.00E-04	0.001822	12.028461
229463_at	2.00E-04	0.001822	43.50667907	235885_at	2.00E-04	0.001822	11.994918
239132_at	2.00E-04	0.001822	40.03142852	1557366_at	2.00E-04	0.001822	11.919112
208396_s_at	2.00E-04	0.001822	39.21835696	212226_s_at	2.00E-04	0.001822	11.865441
226311_at	2.00E-04	0.001822	38.53546667	206349_at	2.00E-04	0.001822	11.738618
221796_at	2.00E-04	0.001822	37.91808761	231341_at	2.00E-04	0.001822	11.590542
236095_at	2.00E-04	0.001822	37.60403661	205044_at	2.00E-04	0.001822	11.55303
221795_at	2.00E-04	0.001822	36.88720831	234212_at	2.00E-04	0.001822	11.515284
242036_x_at	2.00E-04	0.001822	34.94999784	212230_at	2.00E-04	0.001822	11.396584
213438_at	2.00E-04	0.001822	31.35239407	1558680_s_at	2.00E-04	0.001822	11.126979
206002_at	2.00E-04	0.001822	28.74558004	224724_at	2.00E-04	0.001822	10.869494
223600_s_at	2.00E-04	0.001822	28.05644632	1556641_at	2.00E-04	0.001822	10.785784
225491_at	2.00E-04	0.001822	26.71949781	209988_s_at	2.00E-04	0.001822	10.564745
233547_x_at	2.00E-04	0.001822	26.58580194	231261_at	2.00E-04	0.001822	10.524531
220180_at	2.00E-04	0.001822	26.46174298	206089_at	2.00E-04	0.001822	10.188125
205413_at	2.00E-04	0.001822	25.85636246	209937_at	2.00E-04	0.001822	10.13084
213841_at	2.00E-04	0.001822	24.66041959	232074_at	2.00E-04	0.001822	10.094932
228708_at	2.00E-04	0.001822	24.05526414	1564807_at	2.00E-04	0.001822	9.9525546
229372_at	2.00E-04	0.001822	20.8074848	242516_x_at	2.00E-04	0.001822	9.9096385
235375_x_at	2.00E-04	0.001822	20.24189114	217380_s_at	2.00E-04	0.001822	9.8890194
206290_s_at	2.00E-04	0.001822	19.96346483	229942_at	2.00E-04	0.001822	9.6332808
229273_at	2.00E-04	0.001822	19.09509197	205151_s_at	2.00E-04	0.001822	9.6011266
223235_s_at	2.00E-04	0.001822	18.53942152	227782_at	2.00E-04	0.001822	9.4692443
230218_at	2.00E-04	0.001822	18.13331638	241672_at	2.00E-04	0.001822	9.4363192
205722_s_at	2.00E-04	0.001822	17.98194082	203962_s_at	2.00E-04	0.001822	9.3751318
236234_at	2.00E-04	0.001822	17.54971048	1554500_a_at	2.00E-04	0.001822	9.3726185
224102_at	2.00E-04	0.001822	16.91026091	201008_s_at	2.00E-04	0.001822	9.3592347
220889_s_at	2.00E-04	0.001822	16.61696294	235957_at	2.00E-04	0.001822	9.3286388
205721_at	2.00E-04	0.001822	16.02338405	214146_s_at	2.00E-04	0.001822	9.2713995
222072_at	2.00E-04	0.001822	16.01223584	227070_at	2.00E-04	0.001822	9.1238973
209355_s_at	2.00E-04	0.001822	16.00025818	224530_s_at	2.00E-04	0.001822	9.0742945
227654_at	2.00E-04	0.001822	15.12117106	203504_s_at	2.00E-04	0.001822	8.9896534
238178_at	2.00E-04	0.001822	14.64807386	220213_at	2.00E-04	0.001822	8.9662471
1553266_at	2.00E-04	0.001822	14.41188182	240532_at	2.00E-04	0.001822	8.9465227
244117_at	2.00E-04	0.001822	13.61716933	238332_at	2.00E-04	0.001822	8.8970457
219607_s_at	2.00E-04	0.001822	13.39962131	241235_at	2.00E-04	0.001822	8.8028268
229580_at	2.00E-04	0.001822	13.3297205	206014_at	2.00E-04	0.001822	8.7664187
219511_s_at	2.00E-04	0.001822	13.23942057	209987_s_at	2.00E-04	0.001822	8.6654481
203505_at	2.00E-04	0.001822	13.13639458	208510_s_at	2.00E-04	0.001822	8.6638807
205918_at	2.00E-04	0.001822	13.13297986	201010_s_at	2.00E-04	0.001822	8.6233238
232390_at	2.00E-04	0.001822	13.12990838	205968_at	2.00E-04	0.001822	8.6068572
237422_at	2.00E-04	0.001822	13.11010154	219697_at	2.00E-04	0.001822	8.5955844
229080_at	2.00E-04	0.001822	12.88048516	1554530_at	2.00E-04	0.001822	8.5285468
230913_at	2.00E-04	0.001822	12.68195591	227020_at	2.00E-04	0.001822	8.481471
219909_at	2.00E-04	0.001822	12.47407653	213768_s_at	2.00E-04	0.001822	8.4303653
215692_s_at	2.00E-04	0.001822	12.36558964	243901_at	2.00E-04	0.001822	8.3547075
205737_at	2.00E-04	0.001822	12.33646348	205150_s_at	2.00E-04	0.001822	8.3316811
203961_at	2.00E-04	0.001822	12.23734479	210547_x_at	2.00E-04	0.001822	8.330207

Table S6 Top 100 DEGs between differentiated and un-differentiated SK-N-SH cells

Probe	Feature P	FDR	Fold change	Probe	Feature P	FDR	Fold change
238047_at	2.00E-04	6.59E-04	3203.68978	221796_at	2.00E-04	6.59E-04	21.24929
219825_at	2.00E-04	6.59E-04	194.14807	224396_s_at	2.00E-04	6.59E-04	21.16005
229463_at	2.00E-04	6.59E-04	108.448517	220180_at	2.00E-04	6.59E-04	20.98868
219669_at	2.00E-04	6.59E-04	67.6496241	1561355_at	2.00E-04	6.59E-04	20.95431
217452_s_at	2.00E-04	6.59E-04	63.6987236	219087_at	2.00E-04	6.59E-04	20.75873
214803_at	2.00E-04	6.59E-04	59.1683489	224184_s_at	2.00E-04	6.59E-04	20.33543
244745_at	2.00E-04	6.59E-04	52.4704105	231120_x_at	2.00E-04	6.59E-04	20.11165
229130_at	2.00E-04	6.59E-04	43.6011592	1567181_x_at	2.00E-04	6.59E-04	20.00993
1558170_at	2.00E-04	6.59E-04	43.5015206	1569315_s_at	2.00E-04	6.59E-04	19.9887
210121_at	2.00E-04	6.59E-04	42.5181231	232434_at	2.00E-04	6.59E-04	19.84672
203854_at	2.00E-04	6.59E-04	38.0411968	228973_at	2.00E-04	6.59E-04	19.62481
235066_at	2.00E-04	6.59E-04	36.706945	227758_at	2.00E-04	6.59E-04	19.61863
230163_at	2.00E-04	6.59E-04	34.9441317	206336_at	2.00E-04	6.59E-04	19.39176
232753_at	2.00E-04	6.59E-04	34.2233223	202575_at	2.00E-04	6.59E-04	18.85743
204818_at	2.00E-04	6.59E-04	34.1698826	1559732_at	2.00E-04	6.59E-04	18.71021
1555564_a_at	2.00E-04	6.59E-04	33.0191493	1560109_s_at	2.00E-04	6.59E-04	18.51255
235278_at	2.00E-04	6.59E-04	32.4824662	220269_at	2.00E-04	6.59E-04	18.46873
230595_at	2.00E-04	6.59E-04	32.3364661	1552825_at	2.00E-04	6.59E-04	18.29287
239132_at	2.00E-04	6.59E-04	31.2645283	1566749_at	2.00E-04	6.59E-04	17.8875
201926_s_at	2.00E-04	6.59E-04	30.9571701	211149_at	2.00E-04	6.59E-04	17.84011
219619_at	2.00E-04	6.59E-04	30.7096447	1554384_at	2.00E-04	6.59E-04	17.76535
206253_at	2.00E-04	6.59E-04	29.0833102	235352_at	2.00E-04	6.59E-04	17.72771
243197_at	2.00E-04	6.59E-04	29.0738291	1553261_x_at	2.00E-04	6.59E-04	17.59608
233160_at	2.00E-04	6.59E-04	28.6767847	236859_at	2.00E-04	6.59E-04	17.46768
243395_at	2.00E-04	6.59E-04	28.2049483	214475_x_at	2.00E-04	6.59E-04	17.44495
244885_at	2.00E-04	6.59E-04	28.1519076	235957_at	2.00E-04	6.59E-04	17.35482
227654_at	2.00E-04	6.59E-04	27.7366497	205590_at	2.00E-04	6.59E-04	17.27926
232390_at	2.00E-04	6.59E-04	27.5342204	243991_at	2.00E-04	6.59E-04	17.25472
202481_at	2.00E-04	6.59E-04	27.43277	202599_s_at	2.00E-04	6.59E-04	17.16
239776_at	2.00E-04	6.59E-04	26.9877097	233675_s_at	2.00E-04	6.59E-04	17.09388
227550_at	2.00E-04	6.59E-04	26.6611666	206556_at	2.00E-04	6.59E-04	16.75814
1554195_a_at	2.00E-04	6.59E-04	26.4853814	236313_at	2.00E-04	6.59E-04	16.75246
225372_at	2.00E-04	6.59E-04	26.4395148	214680_at	2.00E-04	6.59E-04	16.67862
224296_x_at	2.00E-04	6.59E-04	25.5853991	211598_x_at	2.00E-04	6.59E-04	16.40388
240397_x_at	2.00E-04	6.59E-04	25.5629318	206742_at	2.00E-04	6.59E-04	16.39035
223315_at	2.00E-04	6.59E-04	25.2605431	240414_at	2.00E-04	6.59E-04	16.1594
219511_s_at	2.00E-04	6.59E-04	24.9541476	205150_s_at	2.00E-04	6.59E-04	16.10224
207838_x_at	2.00E-04	6.59E-04	24.3800366	223927_at	2.00E-04	6.59E-04	16.05146
228375_at	2.00E-04	6.59E-04	24.2933629	235046_at	2.00E-04	6.59E-04	15.69938
238426_at	2.00E-04	6.59E-04	24.2553691	202820_at	2.00E-04	6.59E-04	15.68345
239632_at	2.00E-04	6.59E-04	24.1991701	228111_s_at	2.00E-04	6.59E-04	15.67017
228335_at	2.00E-04	6.59E-04	23.8703705	229302_at	2.00E-04	6.59E-04	15.54246
1563160_at	2.00E-04	6.59E-04	23.602517	223574_x_at	2.00E-04	6.59E-04	15.32505
229580_at	2.00E-04	6.59E-04	23.5675689	232737_s_at	2.00E-04	6.59E-04	15.27707
1552439_s_at	2.00E-04	6.59E-04	23.4176568	1553834_at	2.00E-04	6.59E-04	15.23532
206392_s_at	2.00E-04	6.59E-04	22.5764057	1565917_at	2.00E-04	6.59E-04	15.21643
209498_at	2.00E-04	6.59E-04	22.5250653	212558_at	2.00E-04	6.59E-04	15.20423
202600_s_at	2.00E-04	6.59E-04	22.4518671	1561574_at	2.00E-04	6.59E-04	15.14245
202016_at	2.00E-04	6.59E-04	21.679026	205151_s_at	2.00E-04	6.59E-04	15.08977
1570208_at	2.00E-04	6.59E-04	21.3955644	1562329_at	2.00E-04	6.59E-04	15.07781

Table S7 Overlapped DEGs in three neuroblastoma cell lines

Probe	GENE SYMBOl	Probe	GENE SYMBOL	Probe	GENE SYMBOL	Probe	GENE SYMBOL	Probe	GENE SYMBOL	Probe	GENE SYMBOL
214956_at	AAK1	220889_s_at	CA10	206159_at	GDF10	227969_at	LOC400960	238085_at	PDE6B	219511_s_at	SNCAIP
238100_at	AAK1	235781_at	CACNA1B	1560227_at	GDPD1	1569453_a_at	LOC692247	1560359_at	PELO	212558_at	SPRY1
203504_s_at	ABCA1	209031_at	CADM1	203157_s_at	GLS	1558796_a_at	LOC728052	242708_at	PEX1	236838_at	SRCIN1
203505_at	ABCA1	209032_s_at	CADM1	205531_s_at	GLS2	1558795_at	LOC728052	222150_s_at	PION	1555912_at	ST7OT1
204567_s_at	ABCG1	231729_s_at	CAPS	214157_at	GNAS	1570572_at	LOC729291	240061_at	PION	221236_s_at	STMN4
213497_at	ABTB2	227091_at	CCDC146	224964_s_at	GNG2	238653_at	LRIG2	213142_x_at	PION	211106_at	SUPT3H
244484_at	ACOT8	220180_at	CCDC68	222005_s_at	GNG3	235066_at	MAP4	229245_at	PLEKHA6	229039_at	SYN2
226311_at	ADAMTS2	227775_at	CELF6	234284_at	GNG8	1553708_at	MGC16075	208510_s_at	PPARG	206162_x_at	SYT5
205882_x_at	ADD3	1555564_a_at	CFI	239533_at	GPR155	241759_at	MGC5566	228010_at	PPP2R2C	201042_at	TGM2
201752_s_at	ADD3	203854_at	CFI	231166_at	GPR155	226644_at	MIB2	214373_at	PPP4R2	213882_at	TM2D1
228215_at	ADD3	235427_at	CFLAR	207183_at	GPR19	232090_at	MIR214	230015_at	PRCD	210130_s_at	TM7SF2
201753_s_at	ADD3	229423_at	CHEK1	204793_at	GPRASP1	203878_s_at	MMP11	209815_at	PTCH1	238426_at	TMEM130
201034_at	ADD3	228335_at	CLDN11	1569154_a_at	GRAMD4	239272_at	MMP28	208131_s_at	PTGIS	239776_at	TMEM232
204499_at	AGTPBP1	212358_at	CLIP3	235957_at	GRIP1	239273_s_at	MMP28	235634_at	PURG	219005_at	TMEM59L
204500_s_at	AGTPBP1	230045_at	CNTN2	209102_s_at	HBP1	219909_at	MMP28	50965_at	RAB26	217853_at	TNS3
208212_s_at	ALK	240985_at	CNTN4	230218_at	HIC1	215692_s_at	MPPED2	219562_at	RAB26	1569154_a_at	TSC22D3
219834_at	ALS2CR8	221019_s_at	COLEC12	205975_s_at	HOXD1	205413_at	MPPED2	204199_at	RALGPS1	220623_s_at	TSGA10
228944_at	AlX3	239765_at	CPEB3	228375_at	IGSF11	219786_at	MTL5	205080_at	RARB	202242_at	TSPAN7
208498_s_at	AMy1A	244403_at	CRB1	227677_at	JAK3	1568857_a_at	NBR1	208530_s_at	RARB	210129_s_at	TTLL3
208498_s_at	AMY1B	230343_at	CST3	211006_s_at	KCNB1	232390_at	NCAM2	244656_at	RASL10B	206072_at	UCN
208498_s_at	AMY1C	206424_at	CYP26A1	205737_at	KCNQ2	205669_at	NCAM2	235488_at	RASL10B	1554141_s_at	WDR78
204671_s_at	ANKRD6	219825_at	CYP26B1	236054_at	KDSR	215571_at	NCAM2	211421_s_at	RET	229816_at	WDR78
204672_s_at	ANKRD6	221886_at	DENND2A	206478_at	KIAA0125	238697_at	NCRNA00086	214120_at	RFPL1S	243526_at	WDR86
228368_at	ARHGAP20	202481_at	DHRS3	235956_at	KIAA1377	227909_at	NCRNA00086	210258_at	RGS13	229849_at	WIPF3
226576_at	ARHGAP26	238032_at	DHRS3	232166_at	KIAA1377	238697_at	NCRNA00087	207383_s_at	RHBDL1	241600_at	WIPF3
229642_at	ARHGEF7	206253_at	DLG2	222139_at	KIAA1466	227909_at	NCRNA00087	223534_s_at	RPS6KL1	217380_s_at	XPNPEP1
242999_at	ARHGEF7	228973_at	DLG2	223600_s_at	KIAA1683	213438_at	NFASC	237081_at	RPSAP49	227020_at	YPEL2
210129_s_at	ARPC4	240857_at	DNAH9	227582_at	KLHDC9	236677_at	NGB	205540_s_at	RRAGB	235916_at	YPEL4
225283_at	ARRDC4	228546_at	DPP6	228328_at	KLHL28	221104_s_at	NIPSNAP3B	1554133_at	RUFY2	228715_at	ZCCHC12
242036_x_at	ATP2B3	232204_at	EBF1	210306_at	L3MBTL	223764_x_at	NIPSNAP3B	203724_s_at	RUFY3	207757_at	ZFP2
228816_at	ATP6AP1l	227646_at	EBF1	202202_s_at	LAMA4	215002_at	NPIPL3	215321_at	RUNDC3B	240642_at	ZMYM2
205198_s_at	ATP7A	229581_at	ELFN1	218922_s_at	LASS4	239293_at	NRSN1	241703_at	RUNDC3B	231717_s_at	ZNF226
240523_at	ATPBD4	229080_at	EMID2	206349_at	LGI1	209915_s_at	NRXN1	1566772_at	SCHIP1	228718_at	ZNF44
213745_at	ATRNL1	229916_at	ENPP6	219136_s_at	LMF1	221796_at	NTRK2	206381_at	SCN2A	1556187_at	ZNF555
205638_at	BAI3	1569554_at	ESR2	240407_at	LOC100126784	214680_at	NTRK2	229057_at	SCN2A	243747_at	ZNF599
37547_at	BBS9	236638_at	FAM123C	236838_at	LOC100128100	221795_at	NTRK2	210432_s_at	SCN3A	238466_at	ZNF91
203685_at	BCL2	222184_at	FAM66E	232090_at	LOC100128178	207152_at	NTRK2	204722_at	SCN3B		
239536_at	BNIPL	1559141_s_at	FAM87A	215002_at	LOC100132247	229463_at	NTRK2	204723_at	SCN3B		
225446_at	BRWD1	1559141_s_at	FAM87B	236838_at	LOC100132856	236095_at	NTRK2	228150_at	SEC16B		
228570_at	BTBD11	1553682_at	FBXL14	236838_at	LOC100133017	1559732_at	NUB1	226627_at	8-Sep		
238692_at	BTBD11	226096_at	FNDC5	229007_at	LOC100134091	1560109_s_at	NUB1	225123_at	SESN3		
209006_s_at	C1ORF63	229007_at	FRG1B	241484_x_at	LOC100134091	241739_at	OGFOD1	1558578_a_at	SLC13A4		
209007_s_at	C1ORF63	241484_x_at	FRG1B	1559141_s_at	LOC100134357	1554526_at	OLFM3	230624_at	SLC25A27		
224989_at	C4ORF34	211458_s_at	GABARAPL1	232794_at	LOC153682	1554524_a_at	OLFM3	1552774_a_at	SLC25A27		
227052_at	C4ORF34	208868_s_at	GABARAPL1	229007_at	LOC283788	221864_at	ORAI3	231341_at	SLC35D3		
207241_at	C4ORF6	211458_s_at	GABARAPL3	241484_x_at	LOC283788	214204_at	PACRG	228221_at	SLC44A3		
1564699_at	C5ORF4	208868_s_at	GABARAPL3	1555363_s_at	LOC284440	207838_x_at	PBXIP1	205918_at	SLC4A3		
235556_at	C5ORF41	223604_at	GARNL3	242852_at	LOC285147	223854_at	PCDHB10	1556641_at	SLC7A14		
225956_at	C5ORF41	227321_at	GATS	228564_at	LOC375295	223854_at	PCDHB9	202508_s_at	SNAP25		
223550_s_at	CA10	1553971_a_at	GATS	230815_at	LOC389765	227088_at	PDE5A	202507_s_at	SNAP25		

Table S8 Functional groups enriched by neuroblastoma differentiation signature genes.

Groups	Function
**Group0**	heterotrimeric G-protein complex
**Group1**	multicellular organismal response to stress
**Group2**	negative regulation of cholesterol storage
**Group3**	cerebellar cortex formation
**Group4**	node of Ranvier
**Group5**	growth cone
**Group6**	axon
**Group7**	main axon
**Group8**	voltage-gated sodium channel complex
**Group9**	ATPase activity, coupled to transmembrane movement of ions
**Group10**	neuron maturation
**Group11**	eye photoreceptor cell differentiation
**Group12**	cell maturation
**Group13**	axon part

Table S9 GO terms enriched by neuroblastoma differentiation signature genes.

NO.	GO ID	Ontology Source	GO Term	GO Groups
1	GO:0005834	GO_CellularComponent	heterotrimeric G-protein complex	[Group0]
2	GO:0019897	GO_CellularComponent	extrinsic component of plasma membrane	[Group0]
3	GO:0031234	GO_CellularComponent	extrinsic component of cytoplasmic side of plasma membrane	[Group0]
4	GO:0033762	GO_BiologicalProcess	response to glucagon	[Group0]
5	GO:0071377	GO_BiologicalProcess	cellular response to glucagon stimulus	[Group0]
6	GO:0033555	GO_BiologicalProcess	multicellular organismal response to stress	[Group1, Group4]
7	GO:0051899	GO_BiologicalProcess	membrane depolarization	[Group1, Group4]
8	GO:0072659	GO_BiologicalProcess	protein localization to plasma membrane	[Group1]
9	GO:0090002	GO_BiologicalProcess	establishment of protein localization to plasma membrane	[Group1]
10	GO:0005496	GO_MolecularFunction	steroid binding	[Group2]
11	GO:0010742	GO_BiologicalProcess	macrophage derived foam cell differentiation	[Group2]
12	GO:0010743	GO_BiologicalProcess	regulation of macrophage derived foam cell differentiation	[Group2]
13	GO:0010745	GO_BiologicalProcess	negative regulation of macrophage derived foam cell differentiation	[Group2]
14	GO:0010874	GO_BiologicalProcess	regulation of cholesterol efflux	[Group2]
15	GO:0010875	GO_BiologicalProcess	positive regulation of cholesterol efflux	[Group2]
16	GO:0010878	GO_BiologicalProcess	cholesterol storage	[Group2]
17	GO:0010883	GO_BiologicalProcess	regulation of lipid storage	[Group2]
18	GO:0010885	GO_BiologicalProcess	regulation of cholesterol storage	[Group2]
19	GO:0010887	GO_BiologicalProcess	negative regulation of cholesterol storage	[Group2]
20	GO:0010888	GO_BiologicalProcess	negative regulation of lipid storage	[Group2]
21	GO:0015485	GO_MolecularFunction	cholesterol binding	[Group2]
22	GO:0015918	GO_BiologicalProcess	sterol transport	[Group2]
23	GO:0019915	GO_BiologicalProcess	lipid storage	[Group2]
24	GO:0030301	GO_BiologicalProcess	cholesterol transport	[Group2]
25	GO:0032368	GO_BiologicalProcess	regulation of lipid transport	[Group2]
26	GO:0032370	GO_BiologicalProcess	positive regulation of lipid transport	[Group2]
27	GO:0032371	GO_BiologicalProcess	regulation of sterol transport	[Group2]
28	GO:0032373	GO_BiologicalProcess	positive regulation of sterol transport	[Group2]
29	GO:0032374	GO_BiologicalProcess	regulation of cholesterol transport	[Group2]
30	GO:0032376	GO_BiologicalProcess	positive regulation of cholesterol transport	[Group2]
31	GO:0032526	GO_BiologicalProcess	response to retinoic acid	[Group2]
32	GO:0032934	GO_MolecularFunction	sterol binding	[Group2]
33	GO:0033293	GO_MolecularFunction	monocarboxylic acid binding	[Group2]
34	GO:0033344	GO_BiologicalProcess	cholesterol efflux	[Group2]
35	GO:0055094	GO_BiologicalProcess	response to lipoprotein particle	[Group2]
36	GO:0071229	GO_BiologicalProcess	cellular response to acid	[Group2]
37	GO:0071300	GO_BiologicalProcess	cellular response to retinoic acid	[Group2]
38	GO:0090077	GO_BiologicalProcess	foam cell differentiation	[Group2]
39	GO:0097006	GO_BiologicalProcess	regulation of plasma lipoprotein particle levels	[Group2]
40	GO:0021533	GO_BiologicalProcess	cell differentiation in hindbrain	[Group3]
41	GO:0021575	GO_BiologicalProcess	hindbrain morphogenesis	[Group3]
42	GO:0021587	GO_BiologicalProcess	cerebellum morphogenesis	[Group3]
43	GO:0021695	GO_BiologicalProcess	cerebellar cortex development	[Group3]
44	GO:0021696	GO_BiologicalProcess	cerebellar cortex morphogenesis	[Group3]
45	GO:0021697	GO_BiologicalProcess	cerebellar cortex formation	[Group3]
46	GO:0033268	GO_CellularComponent	node of Ranvier	[Group4, Group6, Group7, Group8]

(Continued)

NO.	GO ID	Ontology Source	GO Term	GO Groups
47	GO:0007272	GO_BiologicalProcess	ensheathment of neurons	[Group4, Group7]
48	GO:0008366	GO_BiologicalProcess	axon ensheathment	[Group4, Group7]
49	GO:0001656	GO_BiologicalProcess	metanephros development	[Group4]
50	GO:0001657	GO_BiologicalProcess	ureteric bud development	[Group4]
51	GO:0035137	GO_BiologicalProcess	hindlimb morphogenesis	[Group4]
52	GO:0046632	GO_BiologicalProcess	alpha-beta T cell differentiation	[Group4]
53	GO:0048265	GO_BiologicalProcess	response to pain	[Group4]
54	GO:0030426	GO_CellularComponent	growth cone	[Group5]
55	GO:0030427	GO_CellularComponent	site of polarized growth	[Group5]
56	GO:0044304	GO_CellularComponent	main axon	[Group6, Group7, Group8]
57	GO:0030424	GO_CellularComponent	axon	[Group6, Group7]
58	GO:0008076	GO_CellularComponent	voltage-gated potassium channel complex	[Group7, Group8]
59	GO:0034703	GO_CellularComponent	cation channel complex	[Group7, Group8]
60	GO:0034705	GO_CellularComponent	potassium channel complex	[Group7, Group8]
61	GO:0019226	GO_BiologicalProcess	transmission of nerve impulse	[Group7]
62	GO:0019228	GO_BiologicalProcess	neuronal action potential	[Group7]
63	GO:0001518	GO_CellularComponent	voltage-gated sodium channel complex	[Group8]
64	GO:0005248	GO_MolecularFunction	voltage-gated sodium channel activity	[Group8]
65	GO:0005272	GO_MolecularFunction	sodium channel activity	[Group8]
66	GO:0022843	GO_MolecularFunction	voltage-gated cation channel activity	[Group8]
67	GO:0034706	GO_CellularComponent	sodium channel complex	[Group8]
68	GO:0035725	GO_BiologicalProcess	sodium ion transmembrane transport	[Group8]
69	GO:0019829	GO_MolecularFunction	cation-transporting ATPase activity	[Group9]
70	GO:0042625	GO_MolecularFunction	ATPase activity, coupled to transmembrane movement of ions	[Group9]
71	GO:0048469	GO_BiologicalProcess	cell maturation	[Group10, Group12, Group4]
72	GO:0048709	GO_BiologicalProcess	oligodendrocyte differentiation	[Group10, Group12]
73	GO:0042551	GO_BiologicalProcess	neuron maturation	[Group10, Group4, Group7]
74	GO:2001239	GO_BiologicalProcess	regulation of extrinsic apoptotic signaling pathway in absence of ligand	[Group10, Group4]
75	GO:0003707	GO_MolecularFunction	steroid hormone receptor activity	[Group10]
76	GO:0004879	GO_MolecularFunction	ligand-activated sequence-specific DNA binding RNA polymerase II transcription factor activity	[Group10]
77	GO:0098531	GO_MolecularFunction	direct ligand regulated sequence-specific DNA binding transcription factor activity	[Group10]
78	GO:0001754	GO_BiologicalProcess	eye photoreceptor cell differentiation	[Group11]
79	GO:0042461	GO_BiologicalProcess	photoreceptor cell development	[Group11]
80	GO:0042462	GO_BiologicalProcess	eye photoreceptor cell development	[Group11]
81	GO:0046530	GO_BiologicalProcess	photoreceptor cell differentiation	[Group11]
82	GO:0048592	GO_BiologicalProcess	eye morphogenesis	[Group11]
83	GO:0021954	GO_BiologicalProcess	central nervous system neuron development	[Group12]
84	GO:0033267	GO_CellularComponent	axon part	[Group13, Group6, Group7]
85	GO:0035249	GO_BiologicalProcess	synaptic transmission, glutamatergic	[Group13, Group6]
86	GO:0043679	GO_CellularComponent	axon terminus	[Group13, Group6]
87	GO:0051966	GO_BiologicalProcess	regulation of synaptic transmission, glutamatergic	[Group13, Group6]
88	GO:0001505	GO_BiologicalProcess	regulation of neurotransmitter levels	[Group13]
89	GO:0006835	GO_BiologicalProcess	dicarboxylic acid transport	[Group13]
90	GO:0006836	GO_BiologicalProcess	neurotransmitter transport	[Group13]
91	GO:0007269	GO_BiologicalProcess	neurotransmitter secretion	[Group13]
92	GO:0014047	GO_BiologicalProcess	glutamate secretion	[Group13]

(Continued)

NO.	GO ID	Ontology Source	GO Term	GO Groups
93	GO:0030801	GO_BiologicalProcess	positive regulation of cyclic nucleotide metabolic process	[Group13]
94	GO:0030804	GO_BiologicalProcess	positive regulation of cyclic nucleotide biosynthetic process	[Group13]
95	GO:0030810	GO_BiologicalProcess	positive regulation of nucleotide biosynthetic process	[Group13]
96	GO:0030816	GO_BiologicalProcess	positive regulation of cAMP metabolic process	[Group13]
97	GO:0030819	GO_BiologicalProcess	positive regulation of cAMP biosynthetic process	[Group13]
98	GO:0045981	GO_BiologicalProcess	positive regulation of nucleotide metabolic process	[Group13]
99	GO:0046717	GO_BiologicalProcess	acid secretion	[Group13]
100	GO:0050806	GO_BiologicalProcess	positive regulation of synaptic transmission	[Group13]
101	GO:1900373	GO_BiologicalProcess	positive regulation of purine nucleotide biosynthetic process	[Group13]
102	GO:1900544	GO_BiologicalProcess	positive regulation of purine nucleotide metabolic process	[Group13]
103	GO:0000149	GO_MolecularFunction	SNARE binding	[None]
104	GO:0004714	GO_MolecularFunction	transmembrane receptor protein tyrosine kinase activity	[None]
105	GO:0007158	GO_BiologicalProcess	neuron cell-cell adhesion	[None]
106	GO:0010923	GO_BiologicalProcess	negative regulation of phosphatase activity	[None]
107	GO:0030199	GO_BiologicalProcess	collagen fibril organization	[None]
108	GO:0050808	GO_BiologicalProcess	synapse organization	[None]
109	GO:0050873	GO_BiologicalProcess	brown fat cell differentiation	[None]
110	GO:0055037	GO_CellularComponent	recycling endosome	[None]
111	GO:0055117	GO_BiologicalProcess	regulation of cardiac muscle contraction	[None]

Table S10. Number of regulons, signature-regulons, and master regulators in three neuroblastoma cell lines.

**Cell line**	**SH-SY5Y-A**	**SH-SY5Y-E**	**SK-N-SH**
Number of regulons	679	832	832
Number of signature-regulons	323	650	639
Number of master regulators(MRs)	87	85	83
Number of core MRs	30	36	35

Table S11. Master regulators that controlled more than 100 Neuroblastoma differentiation signatures.

Master regulators
ZBTB18, LZTS1, ILF2, HOXD1, ZNF232, SOLH, MYCN, ENO1, DEK, CEBPZ, MYBL1, TCF3, NFIL3, SMARCA4, BAZ1B, ARNTL, STAT3, HIVEP2, MEIS2, FBXO16///ZNF395, ZNF211, ZSCAN18, SLC26A3, TBX19, NFATC3, ZNF20///ZNF625-ZNF20, RORB, KDM5B, ZNF189, MYBL2, EWSR1, PTTG1, MNT, ASCL1, ZNF83, PRDM2, TARDBP, SATB1, NFIB, STAT5A, MEF2A, ESRRG, NFKB1, BTAF1, KLF11, FUBP1, SOX11, TGIF2, FOXJ3, ZNF334, ZSCAN5A, ZSCAN26, TEAD4, CREBL2, RBPJ, FOXM1, E2F1, TFAM, NOTCH2, HMGB1, NFE2L2, MAFB, L3MBTL1, TSC22D3, NRF1, PLAGL2, YWHAE, MYB, DAXX

Table S12. The core MRs and their controlled signatures during neuroblastoma differentiation.

Core MRs	*ZBTB18, NFIB, STAT5A, HOXD1, ESRRG, KLF11, FOXJ3, HIVEP2, ZNF334, MEIS2, ZSCAN18, ZNF211, ZSCAN26, CREBL2, ZNF20, ZNF625, NOTCH2, KDM5B, NFE2L2, L3MBTL1, TSC22D3, ZNF189, MNT, PRDM2, ZNF83, SATB1*
Controlled signatures	*TM7SF2, ADD3, NCAM2, AAK1, GLS, SNAP25, CA10, CACNA1B, MMP28, GABARAPL1, GABARAPL3, RUNDC3B, NRSN1, ZCCHC12, COLEC12, DNAH9, CLIP3, KIAA1377, ADD3-AS1, PRCD, YPEL4, OTTHUMG00000163262, CTC-241N9.1, SLC25A27, CFLAR, JAK3, PTGIS, OTTHUMG00000021298, RP11-268G12.1, GNG3, FLJ41309, ATP6AP1L, CRB1, DPH6-AS1, DLG2, RRAGB, CHEK1, ARPC4-TTLL3, TTLL3, CARF, AGTPBP1, RHBDL1, BRWD1, ZNF44, C1orf63, GRIP1, ANKRD6, DNM3OS, SCN3A, ATP7A, LGI1, TGM2, BCL2, OLFM3, ADAMTS2, CCDC146, PELO, NTRK2, LMF1, SLC7A14, COL26A1, TNS3, BAI3, ARHGAP20, IGSF11, RASL10B, ATRNL1, CYP26B1, 8-Sep, LOC389765, GNG2, GATS, RGS13, LOC100996552, ELFN1, L3MBTL1, SYN2, DPP6, ORAI3, ABCA1, ABTB2, OTTHUMG00000177465, RP11-353N14.2, ARHGEF7, ZNF555, RUFY3, DENND2A, BBS9, C4orf6, EBF1, GSAP, RFPL1S, YPEL2, CREBRF, HOXD1, OTTHUMG00000160053, RP11-774O3.3, SLC44A3, OTTHUMG00000042242, OTTHUMG00000165519, RP11-458D21.2, RP4-791M13.3, PTCH1, GPR155, ZNF599, SCN3B, RAB26, PCBP1-AS1, MAP4, LINC00086, LINC00087, ZFP2, SRCIN1, CFI, SCN2A, WIPF3, LOC100126784, ALK, LINC00965, HBP1, GARNL3, ENPP6, OTTHUMG00000175814, RP11-13L2.4, NFASC, SLC35D3, PURG, GPRASP1, LOC692247, CNTN2, OGFOD1, OTTHUMG00000175572, RP4-791K14.2, ATP2B3, TSPAN7, RUFY2, MMP11, TMEM130, OTTHUMG00000014981, RP3-525N10.2, CPEB3, LOC153682, WDR78*

Table S13 GO clusters that enriched by genes in regulatory network

Annotation Cluster 1 Enrichment Score: 2.951749687403057

Category	Term	PValue	Genes	Fold Enrichment	FDR
GOTERM_CC_FAT	GO:0043005~neuron projection	9.48E-05	ATP7A, ARHGEF7, SCN2A, NTRK2, NFASC, CNTN2, SNAP25, RUFY3	7.118908382	0.113401
GOTERM_CC_FAT	GO:0030426~growth cone	6.78E-04	ARHGEF7, NTRK2, SNAP25, RUFY3	22.54320988	0.808791
GOTERM_CC_FAT	GO:0030427~site of polarized growth	7.16E-04	ARHGEF7, NTRK2, SNAP25, RUFY3	22.13333333	0.853377
GOTERM_BP_FAT	GO:0051960~regulation of nervous system development	0.033887482	BCL2, NTRK2, SNAP25, RUFY3	5.526143791	41.55407

Annotation Cluster 2 Enrichment Score: 1.9214281410467846

Category	Term	PValue	Genes	Fold Enrichment	FDR
GOTERM_CC_FAT	GO:0042995~cell projection	5.23E-05	ATP7A, DNAH9, ARHGEF7, BBS9, SCN2A, NTRK2, NFASC, CNTN2, SNAP25, RUFY3, TTLL3	4.802965088	0.062567
GOTERM_CC_FAT	GO:0043005~neuron projection	9.48E-05	ATP7A, ARHGEF7, SCN2A, NTRK2, NFASC, CNTN2, SNAP25, RUFY3	7.118908382	0.113401
GOTERM_BP_FAT	GO:0048666~neuron development	0.001486208	ATP7A, CRB1, BCL2, NTRK2, NFASC, CNTN2, SNAP25	5.477239863	2.290374
GOTERM_BP_FAT	GO:0000902~cell morphogenesis	0.001905016	ATP7A, CRB1, BCL2, NFASC, CNTN2, SNAP25, TTLL3	5.215686275	2.926871
GOTERM_BP_FAT	GO:0048858~cell projection morphogenesis	0.002039524	ATP7A, BCL2, NFASC, CNTN2, SNAP25, TTLL3	6.496038415	3.130469
GOTERM_BP_FAT	GO:0030030~cell projection organization	0.002250827	ATP7A, DNAH9, BCL2, NFASC, CNTN2, SNAP25, TTLL3	5.045609548	3.449502
GOTERM_BP_FAT	GO:0032990~cell part morphogenesis	0.002468778	ATP7A, BCL2, NFASC, CNTN2, SNAP25, TTLL3	6.216911765	3.777542
GOTERM_BP_FAT	GO:0032989~cellular component morphogenesis	0.003282156	ATP7A, CRB1, BCL2, NFASC, CNTN2, SNAP25, TTLL3	4.677038574	4.992575
GOTERM_BP_FAT	GO:0030182~neuron differentiation	0.005298418	ATP7A, CRB1, BCL2, NTRK2, NFASC, CNTN2, SNAP25	4.239233593	7.94287
GOTERM_BP_FAT	GO:0048812~neuron projection morphogenesis	0.007815611	ATP7A, BCL2, NFASC, CNTN2, SNAP25	6.226640891	11.50585
GOTERM_CC_FAT	GO:0030424~axon	0.014250629	SCN2A, NTRK2, NFASC, CNTN2	7.656184486	15.78465
GOTERM_BP_FAT	GO:0031175~neuron projection development	0.014599588	ATP7A, BCL2, NFASC, CNTN2, SNAP25	5.180759804	20.47639
GOTERM_BP_FAT	GO:0007409~axonogenesis	0.034335022	BCL2, NFASC, CNTN2, SNAP25	5.497510921	41.97443
GOTERM_BP_FAT	GO:0048667~cell morphogenesis involved in neuron differentiation	0.041913779	BCL2, NFASC, CNTN2, SNAP25	5.076648841	48.67705
GOTERM_BP_FAT	GO:0000904~cell morphogenesis involved in differentiation	0.061162788	BCL2, NFASC, CNTN2, SNAP25	4.348441016	62.58911
GOTERM_BP_FAT	GO:0007423~sensory organ development	0.207367643	CRB1, BCL2, NTRK2	3.474955048	97.32274
GOTERM_BP_FAT	GO:0007610~behavior	0.250187741	ATP7A, BCL2, NTRK2, SNAP25	2.262301936	98.87294
GOTERM_CC_FAT	GO:0005887~integral to plasma membrane	0.33213562	TM7SF2, SCN2A, NTRK2, CNTN2, RHBDL1, SNAP25	1.537037037	99.20257
GOTERM_BP_FAT	GO:0007155~cell adhesion	0.738870074	BCL2, NFASC, CNTN2	1.136806723	100
GOTERM_BP_FAT	GO:0022610~biological adhesion	0.73960075	BCL2, NFASC, CNTN2	1.13518503	100

Table S14. Homologous sequences of the 400bp region in 8 species

**Species**	**Mouse**
Chromosome location	Mus musculus strain C57BL/6J, GRCm38.p4,chromosome 13, 58807442 to 58807832
Relative location of NTRK2 homologous gene	897 bp at 3' side: BDNF/NT-3 growth factors receptor isoform a precursor
Sequence	

```
Query  6         AGCCTCTACCGCGATTGTAGAAGAGACTGTGGTGTGAATTAGGGACCGGGAGGCGTCGAA  65
                 || || ||| ||||| || |||||||     ||  ||||||||||||           ||
Sbjct  58807442  AGTCTGTACTGCGAT-GTGGAAGAGAT----GTAAGAATTAGGGACC-----------AA  58807485

Query  66        CGGAGGAACGGTTCatcttagaggtacctggatgtaaatg---cacacacacacacagac  122
                  | || | || | ||| ||||| ||||| || | |||| |   ||||||||| ||||  |
Sbjct  58807486  GGAAGCATCGCTGCATTTTAGATGTACCGGGGTCTAAAGGGGGCACACACACGCACACGC  58807545

Query  123       acacacacgcacgcgcgtGCATGTCTACACGGCCAGGAtgtgtgcgtgtgtgcgcgcgtg  182
                  |||||| |||| | ||  ||   |||| | |||| | ||||||||||    |||||| |
Sbjct  58807546  GCACACATGCACACCCGCACAG--CTACCCCGCCAAGGTGTGTGCGTG----CGCGCGAG  58807599

Query  183       tgtgAACTCCCACATGCTGCTGCTGTCTGCTTCTGGCCAGTGGCACCGATGcctccctcc  242
                 ||||||||||||||   |||||||||||||  ||||||  ||||||| | |||| |||||
Sbjct  58807600  TGTGAACTCCCACA---TGCTGCTGTCTGCGCCTGGCCGCTGGCACC-AAGCCT-CCTCC  58807654

Query  243       tccctgctcgcccccagattcccctcccctccctGGTGCTTTTGTCTGGAGGG-----TG  297
                 |||| ||||||||||||||| |||||||||||    |||||||||||||||||     ||
Sbjct  58807655  TCCCCGCTCGCCCCCAGATT-CCCTCCCCTCCGCCTTGCTTTTGTCTGGAGGGTGCTATG  58807713

Query  298       TTATGGGTTtgtgtgtgtatgagc----------------gtgtgtgtgtTTTTGGATTT  341
                  |||| || |||| |||| || ||                | ||||||||||||||||||
Sbjct  58807714  CTATGCGTGTGTGCGTGTGTGTGCGCGCGCGTGTGTGAGCGTGTGTGTTTTTGGATTT  58807773

Query  342       CAGACTAATTTTCTGGAGTTTCTGCCCCTGCTCTGCGTCAGCCCTCACGTCACTTCGCC  400
                 || ||||||||||||||||||||||||||||||||||||||||||||||||||||||||
Sbjct  58807774  CATACTAATTTTCTGGAGTTTCTGCCCCTGCTCTGCGTCAGCCCTCACGTCACTTCGCC  58807832
```

**Species**	**Rat**
Chromosome location	Rattus norvegicus strain BN; Sprague-Dawley, alternate assembly Rn_Celera, chromosome 17, 5981705 to 5982112
Relative location of NTRK2 homologous gene	893 bp at 5' side: BDNF/NT-3 growth factors receptor isoform 2 precursor
Sequence	

```
Query  41       GAATTAGGGACCG-GGAGGCGTCGAACGGAGGAACGGTTCATCTTAGAGGTACCTGgatg  99
                ||||||| ||||  ||| || || | | | | |||| | ||| | ||||||||| || |
Sbjct  5982112  GAATTAGAGACCAAGGAAGCATCCAGCAGCGCAACGCTGCATTTCAGAGGTACCGGGGTC  5982053

Query  100      taaatgcacacacacacacagacacac------acacgcacgcgcgtgcATGTCTACACG  153
                |||| |  ||||||| | |   | | |      ||| |||| | ||  ||   |||| |
Sbjct  5982052  TAAAGGGGCACACACGCGCGCGCGCGCGCTCACATGCACACCCGCACAG--CTACCCC  5981995

Query  154      GCCaggatgtgtgcgtgtgtgcgcgcgtgtgtgaaCTCCCACATGCTGCTGCTGTCTGCT  213
                |||| | ||||||||||    |||||| ||||||||||||||||||   |||||||||
Sbjct  5981994  GCCAAGGTGTGTGCGTG----CGCGCGAGTGTGAACTCCCACATGCT---GCTGTCTGCG  5981942

Query  214      TCTGGCCAGTGGCACCgatgcctccctcctccctgctcgcccccagattcccctcccctc  273
                 ||||||  ||||||| | |||| ||||||||| |||||||||||||||| ||||||||||
Sbjct  5981941  CCTGGCCGCTGGCACC-AAGCCT-CCTCCTCCCCGCTCGCCCCCAGATT-CCCTCCCCTC  5981885

Query  274      cctggtgCTTTTGTCTGGAGGGTgttatgggtttg--tgtgtgtatgagcgtgtgtgtg-  330
                |    ||||||||||||||||||| ||| | | ||  ||||||   | ||||||| |||
Sbjct  5981884  CGCCTTGCTTTTGTCTGGAGGGTGCTAT-GCTATGCCTGTGTGCGAGTGCGTGTGCGTGC  5981826

Query  331      ---------------------------------------------------tttttggaT  339
                                                                   |||||||||
Sbjct  5981825  ATAGCTGCGTATAACGGATGCACGGGTGTCTTTGCTAGATGACCCTACCGATTTTTGGAT  5981766

Query  340      TTCAGACTAATTTTCTGGAGTTTCTGCCCCTGCTCTGCGTCAGCCCTCACGTCACTTCGC  399
                |||| |||||||||||||||||||||||||||||||||||||||||||||||||||||||
Sbjct  5981765  TTCATACTAATTTTCTGGAGTTTCTGCCCCTGCTCTGCGTCAGCCCTCACGTCACTTCGC  5981706

Query  400      C  400
                |
Sbjct  5981705  C  5981705
```

Species	Monkey
Chromosome location	Macaca mulatta isolate 17573, Mmul_8.0.1, chromosome 15, 101009820 to 101010229
Relative location of NTRK2 homologous gene	1018 bp at 3' side: BDNF/NT-3 growth factors receptor precursor

(Continued)

Sequence

```
Query  1          GAGACAGCCTCTACCGCGATTGTAGAAGAGACTGTGGTGTGAATTAGGGACCGGGAGGCG  60
                  ||| ||||||||||||||||||||||||||||||||||||||||||||||||||||||||
Sbjct  101009820  GAGCCAGCCTCTACCGCGATTGTAGAAGAGACTGTGGTGTGAATTAGGGACCGGGAGGCG  101009879

Query  61         TCGAACGGAGGAACGGTTCATCTTAGAGGTACCTGGAtgtaaatgcacacacacacacag  120
                  || |||||||||||||||||||| |||||||||||||||||||| ||||||||||||||
Sbjct  101009880  TCAAACGGAGGAACGGTTCATCTCAGAGGTACCTGGATGTAAATTCACACACACACACA-  101009938

Query  121        acacacacacgcacgcgcgtGCATGTCTACACGGCCaggatgtgtgcgtgtgtgcg----  176
                   |||||| | ||||||||| || |||||||||||||||||||||||||||||||||
Sbjct  101009939  -CACACAGAGGCACGCGCGCGCGTGTCTACACGGCCAGGATGTGTGCGTGTGTGCGTGCG  101009997

Query  177        --------cgcgtgtgtgaaCTCCCACATGCTGCTGCTGTCTGCTTCTGGCCAGTGGCAC  228
                          |||||||||| |||||||||||||||||||||||||||||||||||||||||
Sbjct  101009998  TGTGTGCGCGCGTGTGTGCACTCCCACATGCTGCTGCTGTCTGCTTCTGGCCAGTGGCAC  101010057

Query  229        CgatgcctccctcctccctgctcgcccccagattcccctcccctccctggtgCTTTTGTC  288
                   |||||||||||||||||||||||||||||||||||||||||||||||||||||||||||
Sbjct  101010058  TGATGCCTCCCTCCTCCCTGCTCGCCCCCAGATTCCCCTCCCCTCCCTGGTGCTTTTGTC  101010117

Query  289        TGGAGGGTGTTatgggtttgtgtgtgtatgagcgtgtgtgtgtttttggaTTTCAGACTA  348
                  |||||||||||||||||||||||||||||||||| |||||||||||||||||||||||||
Sbjct  101010118  TGGAGGGTGTTATGGGTTTGTGTGTGTATGAGCGCGTGTGTGTTTTTGGATTTCAGACTA  101010177

Query  349        ATTTTCTGGAGTTTCTGCCCCTGCTCTGCGTCAGCCCTCACGTCACTTCGCC  400
                  ||||||||||||||||||||||||||||||||||||||||||||||||||||
Sbjct  101010178  ATTTTCTGGAGTTTCTGCCCCTGCTCTGCGTCAGCCCTCACGTCACTTCGCC  101010229
```

**Species**	**Rabbit**
Chromosome location	Oryctolagus cuniculus breed Thorbecke inbred unplaced genomic scaffold, OryCun2.0, chr19, 24051814 to 24051857
Relative location of NTRK2 homologous gene	N/A

Sequence

```
Query  288        CTGGAGGGTGTTatgggtttgtgtgtgtatgagcgtgtgtgtgt  331
                  ||||||||||| | |||| ||||||||| ||   ||||||||||
Sbjct  24051857   CTGGAGGGTGTCAGGGGTGTGTGTGTGTGTGTGTATGTGTGTGTGT  24051814
```

**Species**	**Pig**
Chromosome location	Sus scrofa breed mixed, Sscrofa10.2, chromosome 10, 34559418 to 34559809
Relative location of NTRK2 homologous gene	208611 bp at 5' side: BDNF/NT-3 growth factors receptor

Sequence

```
Query  1          GAGACAGCCTCTACCGCGATTGTAGAAGAGACTGTGGTGTGAATTAGGGACCGGGAGGCG  60
                  ||||| ||||||| | ||||| || ||||||| | |||||||||||||||| ||||||||
Sbjct  34559809   GAGACTGCCTCTATCCCGATTTTACAAGAGACCGCGGTGTGAATTAGGGACTGGGAGGCG  34559750

Query  61         TCGAAC-GGAGGAACGGTTCATCTTAGAGGTACCTGGAtgtaaatgcacacacacacaca  119
                  ||  |  |||||||| || ||||| |||||||||||||| ||||||||| |||||||||
Sbjct  34559749   TCATAGAGGAGGAACAGTCCATCTCAGAGGTACCTGGATCTAAATGCACGCACACACACG  34559690

Query  120        gacacacacacgcacgcgcgtgCATGTCTACACGGCCaggatgtgtgcgtgtgtgcgcgc  179
                    || ||||| || |||||||      |||||||| |||| |||||||| |||||||||
Sbjct  34559689   --CAGACACAGGCGCGCGCGT------CTACACGGACAGGCTGTGTGCGGGTGTGCGCG-  34559639

Query  180        gtgtgtgaactcccacatgctgctgCTG---TCTGCTTCTGGCCAGTGGCACCgatgcct  236
                   |||||||||||||||||||||||||||   |||||||||||||||||||||||| ||||
Sbjct  34559638   -TGTGTGAACTCCCACATGCTGCTGCTGCTGTCTGCTTCTGGCCAGTGGCACCGACGCCT  34559580

Query  237        ccctcctccctgctcgcccccagattcccctcccctccctggtgcTTTTGTCTGGAGGGT  296
                  ||||||||||||| ||||||||||||||||||||||||| | ||||||||||||||||||
Sbjct  34559579   CCCTCCTCCCTGCACGCCCCCAGATTCCCCTCCCCTCCCCGTTGCTTTTGTCTGGAGGGT  34559520

Query  297        GTTatgggtttgtgtgtgtatgagcgtgtgtgtgtttttggaTTTCAGACTAATTTTCTG  356
                  |||||||||||||||||||||||||  |||||||||||||||||||||||||||||||||
Sbjct  34559519   GTTATGGGTTTGTGTGTGTATGAGC--GTGTGTGTTTTTGGATTTCAGACTAATTTTCTG  34559462

Query  357        GAGTTTCTGCCCCTGCTCTGCGTCAGCCCTCACGTCACTTCGCC  400
                  ||||||||||||||||||||||||||||||||||||||||||||
Sbjct  34559461   GAGTTTCTGCCCCTGCTCTGCGTCAGCCCTCACGTCACTTCGCC  34559418
```

Species	Pongo
Chromosome location	Pongo abelii isolate ISIS 71, P_pygmaeus_2.0.2, chromosome 9, 79817732 to 79818133
Relative location of NTRK2 homologous gene	1014 bp at 3' side: BDNF/NT-3 growth factors receptor isoform X1

(Continued)

Sequence

```
Query  1         GAGACAGCCTCTACCGCGATTGTAGAAGAGACTGTGGTGTGAATTAGGGACCGGGAGGCG  60
                 ||||||||||||||||||||||||||||||||||||||||||||||||||||||||||||
Sbjct  79817732  GAGACAGCCTCTACCGCGATTGTAGAAGAGACTGTGGTGTGAATTAGGGACCGGGAGGCG  79817791

Query  61        TCGAACGGAGGAACGGTTCATCTTAGAGGTACCTGGatgtaaatgcacacacacacacag  120
                 ||||||||||||||||||||||||||||||||||||||||||||||||||||||||||| 
Sbjct  79817792  TCGAACGGAGGAACGGTTCATCTTAGAGGTACCTGGATGTAAATGCACACACACACACAC  79817851

Query  121       acacacacac--gcacgcgcgtGCATGTCTACACGGCCaggatgtgtgcgtgtgtgcgcg  178
                 ||||||||||  |||||||||||| |||||||||||||||||||||||||||||||||||
Sbjct  79817852  ACACACACACACGCACGCGCGTGCGTGTCTACACGGCCAGGATGTGTGCGTGTGTGCGCG  79817911

Query  179       cgtgtgtgaaCTCCCACATGCTGCTGCTGTCTGCTTCTGGCCAGTGGCACCGatgcctcc  238
                 ||||||||||||||||||||||||||||||||||||||||||||||||||||||||||||
Sbjct  79817912  CGTGTGTGAACTCCCACATGCTGCTGCTGTCTGCTTCTGGCCAGTGGCACCGATGCCTCC  79817971

Query  239       ctcctccctgctcgcccccagattcccctcccctccctggtgCTTTTGTCTGGAGGGTGT  298
                 ||||||||||||||||||||||||||||||||||||||||||||||||||||||||||||
Sbjct  79817972  CTCCTCCCTGCTCGCCCCCAGATTCCCCTCCCCTCCCTGGTGCTTTTGTCTGGAGGGTGT  79818031

Query  299       TatgggtttgtgtgtgtatgagcgtgtgtgtgtttttggaTTTCAGACTAATTTTCTGGA  358
                 ||||||||||||||||||||||||||||||||||||||||||||||||||||||||||||
Sbjct  79818032  TATGGGTTTGTGTGTGTATGAGCGTGTGTGTGTTTTTGGATTTCAGACTAATTTTCTGGA  79818091

Query  359       GTTTCTGCCCCTGCTCTGCGTCAGCCCTCACGTCACTTCGCC  400
                 ||||||||||||||||||||||||||||||||||||||||||
Sbjct  79818092  GTTTCTGCCCCTGCTCTGCGTCAGCCCTCACGTCACTTCGCC  79818133
```

Species	Horse
Chromosome location	Equus caballus isolate Twilight breed thoroughbred, EquCab2.0, chromosome 23, 5442921 to 5443319
Relative location of NTRK2 homologous gene	1010 bp at 5' side: BDNF/NT-3 growth factors receptor isoform X4

Sequence

```
Query  1        GAGACAGCCTCTACCGCGATTGTAGAAGAGACTGTGGTGTGAATTAGGGACCGGGAGGCG  60
                |||||||||||||| |||| |||||||||||| | ||||||||||||||||| |||||||
Sbjct  5443319  GAGACAGCCTCTACTGCGAATGTAGAAGAGACCGAGGTGTGAATTAGGGACCAGGAGGCG  5443260

Query  61       TCGAACGGAGGAACGGTTCATCTTAGAGGTACCTGGAtgtaaatgca--cacacacacac  118
                |||| ||| |||||||| ||||| |||||||||||||| |||| |||  |||| ||||||
Sbjct  5443259  TCGAGCGGCGGAACGGTCCATCTCAGAGGTACCTGGATCTAAACGCAGTCACATACACAC  5443200

Query  119      agacacacacacgcacgc--gcgtGCATGTCTACACGGCCaggatgtgtgcgtgtgtgcg  176
                 | | | | ||| ||| |  ||| ||  ||||||||| ||||||||||||||||||||||
Sbjct  5443199  GGGCGCCCCCACACACACAGGCGCGCGCGTCTACACG-CCAGGATGTGTGCGTGTGTGCG  5443141

Query  177      cgcgtgtgtgaaCTCCCACATGCTGCTGCTGTCTGCTTCTGGCCAGTGGCACCgatgcct  236
                ||  |||||||||||||||||||||||||||||||||||||||||||||||||||| |||
Sbjct  5443140  CG--TGTGTGAACTCCCACATGCTGCTGCTGTCTGCTTCTGGCCAGTGGCACCGATACCT  5443083

Query  237      ccctcctccctgctcgcccccagattcccctcccctccctggtgcTTTTGTCTGGAGGGT  296
                |||||||||| |||||||||||| |||||||||| |||||| ||||||||||||||||||
Sbjct  5443082  CCCTCCTCCCCGCTCGCCCCCAGCTTCCCCTCCCATCCCTGTTGCTTTTGTCTGGAGGGT  5443023

Query  297      GTTatgggtttgtgtgtgtatgagcgtgtgtgtgtttttggaTTTCAGACTAATTTTCTG  356
                |||||||||||||||||||||||||  |||||||||||||||||||||||||||||||||
Sbjct  5443022  GTTATGGGTTTGTGTGTGTATGAGC--GTGTGTGTTTTTGGATTTCAGACTAATTTTCTG  5442965

Query  357      GAGTTTCTGCCCCTGCTCTGCGTCAGCCCTCACGTCACTTCGCC  400
                ||||||||||||||||||||||||||||||||||||||||||||
Sbjct  5442964  GAGTTTCTGCCCCTGCTCTGCGTCAGCCCTCACGTCACTTCGCC  5442921
```

Species	Zebrafish
Chromosome location	Danio rerio strain Tuebingen, GRCz10, chromosome 1, 12660966 to 12661036
Relative location of NTRK2 homologous gene	N/A

Sequence

```
Query  301       tgggtttgtgtgtgtatgagcgtgtgtgtgtttttggatTTCAGACTAATTT-TCTGGAG  359
                 || || ||||||||| || | |||||||||| | || ||   ||||   |||  || || 
Sbjct  12661036  TGTGTGTGTGTGTGTGTGTGTGTGTGTGTGTGTGTGCATGACAGATGGATTCCTCAGGC-  12660978

Query  360       TTTCTGCCCCTG  371
                 ||||||||||||
Sbjct  12660977  TTTCTGCCCCTG  12660966
```

Table S15. Homologous sequences of the 118 bp region in 9 species.

Species	Mouse
Chromosome location	Mus musculus strain C57BL/6J, GRCm38.p4,chromosome 13, 58807746 to 58807831
Relative location of NTRK2 homologous gene	898 bp at 3' side: BDNF/NT-3 growth factors receptor isoform a precursor
Sequence	

```
Query  31        gtgtatgagcgtgtgtgtgtTTTTGGATTTCAGACTAATTTTCTGGAGTTTCTGCCCCTG  90
                 |||| |||||||||||||  |||||||||||| |||||||||||||||||||||||||||
Sbjct  58807746  GTGTGTGAGCGTGTGTGT--TTTTGGATTTCATACTAATTTTCTGGAGTTTCTGCCCCTG  58807803

Query  91        CTCTGCGTCAGCCCTCACGTCACTTCGC  118
                 ||||||||||||||||||||||||||||
Sbjct  58807804  CTCTGCGTCAGCCCTCACGTCACTTCGC  58807831
```

Species	Rat
Chromosome location	Rattus norvegicus strain mixed, Rnor_6.0, chromosome 17, 6244472 to 6244568
Relative location of NTRK2 homologous gene	894 bp at 5' side: BDNF/NT-3 growth factors receptor isoform 2 precursor
Sequence	

```
Query  20       tgggtttgtgtgtgtatgagcgtgtgtgtttttggaTTTCAGACTAATTTTCTGGAGT  79
                || || ||||||||| |||||||||||||  ||||||||||||| ||||||||||||||||
Sbjct  6244568  TGTGTGTGTGTGTGTGAGCGTGTGTGT--TTTTGGATTTCATACTAATTTTCTGGAGT  6244511

Query  80       TTCTGCCCCTGCTCTGCGTCAGCCCTCACGTCACTTCGC  118
                |||||||||||||||||||||||||||||||||||||||
Sbjct  6244510  TTCTGCCCCTGCTCTGCGTCAGCCCTCACGTCACTTCGC  6244472
```

Species	Monkey
Chromosome location	Macaca mulatta isolate 17573, Mmul_8.0.1, chromosome 15, 101010111 to 101010228
Relative location of NTRK2 homologous gene	1019 bp at 3' side: BDNF/NT-3 growth factors receptor precursor
Sequence	

```
Query  1          TTTTGTCTGGAGGGTGTTatgggtttgtgtgtgtatgagcgtgtgtgtgtttttggaTTT  60
                  ||||||||||||||||||||||||||||||||||||||||| ||||||||||||||||||
Sbjct  101010111  TTTTGTCTGGAGGGTGTTATGGGTTTGTGTGTGTATGAGCGCGTGTGTGTTTTTGGATTT  101010170

Query  61         CAGACTAATTTTCTGGAGTTTCTGCCCCTGCTCTGCGTCAGCCCTCACGTCACTTCGC  118
                  ||||||||||||||||||||||||||||||||||||||||||||||||||||||||||
Sbjct  101010171  CAGACTAATTTTCTGGAGTTTCTGCCCCTGCTCTGCGTCAGCCCTCACGTCACTTCGC  101010228
```

Species	Rabbit
Chromosome location	Oryctolagus cuniculus breed Thorbecke inbred unplaced genomic scaffold, OryCun2.0, chrUn0030, 1681610 to 1681725
Relative location of NTRK2 homologous gene	N/A
Sequence	

```
Query  1        TTTTGTCTGGAGGGTGTTatgggtttgtgtgtgtatgagcgtgtgtgtgttttggattt  60
                ||||||||||||||||| ||||| ||||||||||||||||||||||||  ||||||||||
Sbjct  1681610  TTTTGTCTGGAGGGTGTGATGGGCTTGTGTGTGTATGAGCGTGTGTGT--TTTTGGATTT  1681667

Query  61       cagactaattttctggagtttctgccCCTGCTCTGCGTCAGCCCTCACGTCACTTCGC  118
                ||||||||||||||||||||||||||||||||||||||||||||||||||||||||||
Sbjct  1681668  CAGACTAATTTTCTGGAGTTTCTGCCCCTGCTCTGCGTCAGCCCTCACGTCACTTCGC  1681725
```

Species	Pig
Chromosome location	Sus scrofa breed mixed, Sscrofa10.2, chromosome 10, 34559419 to 34559534
Relative location of NTRK2 homologous gene	208612 bp at 5' side: BDNF/NT-3 growth factors receptor
Sequence	

```
Query  1         TTTTGTCTGGAGGGTGTTatgggtttgtgtgtgtatgagcgtgtgtgtgtttttggaTTT  60
                 ||||||||||||||||||||||||||||||||||||||||||||||||  ||||||||||
Sbjct  34559534  TTTTGTCTGGAGGGTGTTATGGGTTTGTGTGTGTATGAGCGTGTGTGT--TTTTGGATTT  34559477

Query  61        CAGACTAATTTTCTGGAGTTTCTGCCCCTGCTCTGCGTCAGCCCTCACGTCACTTCGC  118
                 ||||||||||||||||||||||||||||||||||||||||||||||||||||||||||
Sbjct  34559476  CAGACTAATTTTCTGGAGTTTCTGCCCCTGCTCTGCGTCAGCCCTCACGTCACTTCGC  34559419
```

Species	Pongo
Chromosome location	Pongo abelii isolate ISIS 71, P_pygmaeus_2.0.2, chromosome 9, 79818015 to 79818132
Relative location of NTRK2 homologous gene	1015 bp at 3' side: BDNF/NT-3 growth factors receptor isoform X1
Sequence	

```
Query  1         TTTTGTCTGGAGGGTGTTatgggtttgtgtgtgtatgagcgtgtgtgtgtttttggaTTT  60
                 ||||||||||||||||||||||||||||||||||||||||||||||||||||||||||||
Sbjct  79818015  TTTTGTCTGGAGGGTGTTATGGGTTTGTGTGTGTATGAGCGTGTGTGTGTTTTTGGATTT  79818074

Query  61        CAGACTAATTTTCTGGAGTTTCTGCCCCTGCTCTGCGTCAGCCCTCACGTCACTTCGC  118
                 ||||||||||||||||||||||||||||||||||||||||||||||||||||||||||
Sbjct  79818075  CAGACTAATTTTCTGGAGTTTCTGCCCCTGCTCTGCGTCAGCCCTCACGTCACTTCGC  79818132
```

(Continued)

Species	Horse
Chromosome location	Equus caballus isolate Twilight breed thoroughbred, EquCab2.0, chromosome 23, 5442922 to 5443037
Relative location of NTRK2 homologous gene	1011 bp at 5' side: BDNF/NT-3 growth factors receptor isoform X4
Sequence	

```
Query  1        TTTTGTCTGGAGGGTGTTatgggtttgtgtgtgtatgagcgtgtgtgtgtttttggaTTT  60
                ||||||||||||||||||||||||||||||||||||||||||||||||  ||||||||||
Sbjct  5443037  TTTTGTCTGGAGGGTGTTATGGGTTTGTGTGTGTATGAGCGTGTGTGT--TTTTGGATTT  5442980

Query  61       CAGACTAATTTTCTGGAGTTTCTGCCCCTGCTCTGCGTCAGCCCTCACGTCACTTCGC  118
                ||||||||||||||||||||||||||||||||||||||||||||||||||||||||||
Sbjct  5442979  CAGACTAATTTTCTGGAGTTTCTGCCCCTGCTCTGCGTCAGCCCTCACGTCACTTCGC  5442922
```

Species	Zebrafish
Chromosome location	Danio rerio strain Tuebingen, GRCz10, chromosome 1, 12660966 to 12661036
Relative location of NTRK2 homologous gene	N/A
Sequence	

```
Query  20        tgggtttgtgtgtgtatgagcgtgtgtgtgtttttggatTTCAGACTAATTT-TCTGGAG  78
                 || || ||||||||| || | |||||||||| | || ||  ||||   |||  || ||
Sbjct  12661036  TGTGTGTGTGTGTGTGTGTGTGTGTGTGTGTGTGTGTGCATGACAGATGGATTCCTCAGGC-  12660978

Query  79        TTTCTGCCCCTG  90
                 ||||||||||||
Sbjct  12660977  TTTCTGCCCCTG  12660966
```

Species	Fruit fly
Chromosome location	Drosophila melanogaster, complete sequence, chromosome 2R, 6962291 to 6962328
Relative location of NTRK2 homologous gene	N/A
Sequence	

```
Query  14       GTGTTATGGGTTtgtgtgtgtatgagcgtgtgtgtgtT  51
                |||| ||||||||||||| || || | |||||||||||
Sbjct  6962328  GTGTCATGGGTTTGTGTGCGTCTGTGTGTGTGTGTGTT  6962291
```
